# Quality Assessment and Model Training Impact of Synthetic Images in Medical Diagnostic Imaging: Systematic Review

**DOI:** 10.2196/81472

**Published:** 2026-10-15

**Authors:** Júlia Folguera-Profitós, Marina Ramiro-Pareta, Gerard Carot-Sans, Alba Jiménez-Rueda, Júlia Aznar-Roig, Didier Domínguez-Herrera, Maria Mercedes Nogueras, Esmaeil Nadimi, Ferran Torres, Jordi Piera-Jiménez

**Affiliations:** 1 Information Systems Department, Catalan Health Service (CatSalut), Gran Via de les Corts Catalanes, 587, Barcelona, 08007, Spain, +34 93 403 85 85; 2 Digitalization for the Sustainability of the Heathcare System (DS3) Research Group, Barcelona, Spain; 3 Fundació Tic Salut i Social (FTSS), Barcelona, Spain; 4 Process Unit, Directorate of Quality, Processes and Innovation, Vall d’Hebron University Hospital, Barcelona, Spain; 5 Faculty of Engineering, Maersk Mc-Kinney Moller Institute, University of Southern Denmark, Odense, Denmark; 6 Biostatistics Unit, Medical School, Universitat Autònoma de Barcelona, Bellaterra, Spain

**Keywords:** synthetic images, synthetic data, medical imaging, diagnostic imaging, AI, generative models, image quality assessment, data augmentation, downstream model performance, systematic review

## Abstract

**Background:**

AI has increasingly been used to generate synthetic images to overcome the scarcity of large, regulatory-compliant medical image datasets. However, their quality and actual utility for algorithm development across deep learning architectures, medical settings, and image types have been poorly assessed.

**Objective:**

This study aimed to review the use of synthetic images for algorithm development and to assess their quality and effects on downstream algorithms trained on these images, according to AI architecture, medical setting, and image type.

**Methods:**

The authors conducted a systematic review of studies published between January 2020 and September 2025. PubMed, Scopus, IEEE Xplore, and Embase were searched for peer-reviewed studies evaluating synthetic images generated from human medical imaging data. Data were extracted for imaging modalities, generative architectures, image-quality assessment methods, downstream model performance, explainability approaches, reproducibility practices, and risk of bias. Given substantial heterogeneity and incomplete statistical reporting, findings were synthesized descriptively.

**Results:**

A total of 160 studies were included. Generative adversarial network–based architectures dominated image generation (119 studies, 74.4%), although hybrid and diffusion-based approaches became increasingly represented in recent years. Image-quality evaluation was highly heterogeneous: 50 studies included human-reader evidence, and 101 reported an automated image-quality assessment. Synthetic-only training generally underperformed real-data training, including a median Dice similarity coefficient change of –10.2% for segmentation. Adding synthetic data to real training sets was associated with median improvements in classification precision (+7.8%), recall (+5.3%), *F*_1_-score (+5.5%), accuracy (+4.3%), and area under the curve (+4.0%), while the median Dice coefficient improvement was +1.2%. Explainability evidence was reported in 68 (42.5%) studies and was rarely validated against expert annotations. Reproducibility reporting was inconsistent, and only 38% (60/160) of studies were judged to have low overall risk of bias.

**Conclusions:**

Unlike previous reviews restricted to particular diseases, imaging modalities, or generative architectures, this review provides a cross-domain quantitative synthesis of both synthetic-image quality assessment and downstream model performance. Synthetic images currently appear more valuable as a complement to real training data than as a replacement, with modest improvements in classification performance, smaller gains in segmentation, and generally poorer performance with synthetic-only training. For researchers, developers, and regulators, these findings highlight the need for standardized multimethod image-quality evaluation, complete reporting, and rigorous external validation before synthetic-image pipelines are translated into clinical AI applications.

## Introduction

AI is a potential game changer in diagnostic imaging, with deep learning models already assisting radiologists in making earlier and more accurate decisions [1,2]. However, the development of robust models for image recognition requires large datasets of medical images that are often unavailable for research due to scarcity (eg, in low-prevalence conditions), strict privacy regulations, fragmented storage, and the burden of annotation.

Several strategies have been proposed to overcome data scarcity, including classical augmentation, self-supervised learning, federated learning, and other privacy-preserving techniques [3-5]. In this context, generative AI offers a powerful tool for generating synthetic images that are realistic and, at the same time, deidentified, enabling multicenter sharing without exposing patient information [6-8]. Early successes came from simple geometric or color transformations [9,10], but the field advanced rapidly with generative adversarial networks (GANs) [11,12] and is now expanding to more complex architectures, hybrid models, variational autoencoders (VAEs), and diffusion models [13].

Despite this momentum, the real-world quality of synthetic images and the downstream impact of these images on model performance have been poorly evaluated. Existing reviews primarily focus on specific diseases or imaging modalities, limiting the generalizability of their conclusions across medical imaging domains [14-22]. Others have addressed synthetic data in the broader context of medical AI, combining synthetic images with other data modalities such as electronic health records, time series, and clinical text rather than specifically evaluating synthetic imaging [23]. Some reviews have considered classical image augmentation techniques or image-to-image translation together with generative image synthesis, making it difficult to isolate the contribution of synthetic-image generation itself [24,25]. Conversely, other reviews have restricted their scope to specific generative paradigms, particularly GAN-based methods [18,26], or to publicly available imaging datasets, overlooking the broader range of data sources and evaluation strategies currently used [27]. Consequently, there is still no comprehensive synthesis spanning imaging modalities, clinical applications, generative architectures, image-quality assessment, downstream model performance, explainability, reproducibility, and methodological quality. Moreover, no review has quantitatively synthesized the reported values of image-quality metrics or the changes in downstream performance associated with synthetic-only and synthetic-augmented training. This lack of comprehensive benchmarking limits the ability of researchers, clinicians, and regulators to determine when synthetic images provide meaningful benefits, where their limitations lie, and which evaluation strategies are most appropriate across different clinical and technical settings.

In this systematic review, we comprehensively evaluate the use of synthetic images in medical imaging by characterizing the generative architectures, imaging modalities, clinical applications, and evaluation strategies reported in the literature. We provide a quantitative cross-study synthesis of image-quality metrics and downstream model performance, comparing synthetic-only and synthetic-augmented training across different tasks and imaging modalities. We also examine explainability, reproducibility, and methodological quality to identify current strengths, limitations, and priorities for the responsible development and evaluation of synthetic-image technologies in health care.

## Methods

### Information Sources, Search Strategy, and Eligibility Criteria

This review aimed to assess peer-reviewed studies evaluating de novo synthetic medical images used as training data for algorithms supporting diagnosis or clinical follow-up, irrespective of the clinical condition, imaging modality, or generative model architecture. The review addressed two primary objectives: (1) to characterize the assessment of synthetic-image quality and (2) to evaluate the impact of using synthetic images on downstream model performance. Because downstream utility was central to the review question, studies were required to report at least one quantitative measure of downstream algorithm performance to be eligible.

We searched PubMed/MEDLINE and Scopus on November 28, 2025, for peer-reviewed articles published between January 1, 2020, and September 30, 2025. IEEE Xplore was subsequently searched on December 8, 2025, and Embase was searched on July 9, 2026, using the same publication-date limits. Database-specific search strategies used title- and abstract-based free-text terms for synthetic-image generation, together with database-specific publication, language, subject-area, and content-type restrictions where applicable. The complete search strategies for all databases are provided in Multimedia Appendix 1.

Studies were eligible if they met all of the following criteria: (1) evaluated the effect of synthetic images on the training of algorithms intended to support diagnostic or follow-up decisions, (2) reported at least one quantitative measure of downstream algorithm performance, (3) used human medical imaging data, (4) generated images de novo rather than through image-to-image transformation, (5) were available as full-text articles, and (6) were published in English. The English-language restriction was applied to ensure consistent data extraction and interpretation across the review team.

We excluded the following: (1) studies using synthetic images exclusively for nondiagnostic purposes, such as education or technical simulation, because they did not report clinically relevant performance outcomes; (2) studies focused on microscopic or histopathologic imaging, because their spatial scale, image-generation pipelines, and evaluation methods differ substantially from those used in radiological imaging and would introduce considerable methodological heterogeneity; (3) studies using image-to-image transformation, defined as the generation of an output image from a corresponding source image, including cross-modality, cross-domain, or image-contrast translation, because the review focused specifically on de novo synthetic-image generation rather than the transformation of existing images; and (4) nonprimary or incompletely reported publications, including reviews, editorials, case reports, conference abstracts, preprints, letters, and guidelines, because these publication types did not provide the peer-reviewed, fully reported quantitative evidence required for data extraction and risk-of-bias assessment.

### Study Selection

Two independent reviewers conducted the selection process by screening titles, abstracts, and full texts. Any discrepancies were resolved through group discussion involving the 2 reviewers and at least 2 additional authors.

### Reporting Guidelines and Registration

The review was conducted in accordance with the PRISMA (Preferred Reporting Items for Systematic Reviews and Meta‑Analyses) 2020 guideline [28], the search strategy was reported according to PRISMA-S (Preferred Reporting Items for Systematic Reviews and Meta‑Analyses—Search Extension) guideline [29], and the synthesis without meta-analysis was reported using the Synthesis Without Meta-Analysis (SWiM) guideline [30]; the PRISMA 2020, PRISMA-S, and SWiM checklists are provided in Checklist 1. The review protocol, detailing the objectives, eligibility criteria, and planned analyses, was registered in the PROSPERO (International Prospective Register of Systematic Reviews) database (ID: CRD42023369125) [31].

### Data Extraction and Outcomes

#### Overview

General data on the selected studies included years of publication, clinical domain, imaging modality, image dimensions and resolution, and data source (open access vs in-house). Analysis outcomes included architecture and strategy for image generation, quality of the generated images (metrics used for quality assessment and results), and downstream impact on the performance of the model trained with synthetic images (model architecture, performance metrics used, and results), whenever data were available.

#### Strategy for Image Generation

The architecture for image generation was classified into GANs, variational autoencoder generative adversarial networks (VAE-GANs), diffusion models, and scientific/physical simulation models. GAN-based architectures were further categorized based on structural modifications, including progressive-growing generative adversarial network (PGGAN), style-based generative adversarial network (StyleGAN), conditional GANs, attention-based models (eg, BigGAN [large-scale generative adversarial network]-Deep and SAGAN [Self-Attention Generative Adversarial Network]), and loss-specific GANs (eg, HingeGAN and LSGAN [least squares generative adversarial network]). Hybrid approaches integrating multiple generative paradigms, including VAE-GANs, diffusion-GAN hybrids, and GANs combined with convolutional neural networks (CNNs) or domain-specific models, were documented separately. Table S1 in Multimedia Appendix 1 provides a detailed taxonomy of generative models.

#### Image Quality

Two primary approaches to the assessment of image quality were considered: human-reader assessments and automated metrics of image quality. Human-reader assessments were extracted and classified into task categories (ie, what readers were asked to evaluate) listed in Table S2 in Multimedia Appendix 1. For each category, we extracted the assessment metrics, which included Turing accuracy, synthetic authenticity rate, fooling rate, Likert realism or quality scores, clinical plausibility scores, reader diagnostic accuracy, sensitivity, specificity, area under the curve (AUC), feature-identification accuracy, text-image matching accuracy, preference or ranking metrics, and agreement statistics (Table S3 in Multimedia Appendix 1). Table S4 in Multimedia Appendix 1 provides a guide for interpretation of each of the extracted human-reader metrics for assessing image quality.

Automated metrics for image-quality assessment were grouped into predefined families, including distributional similarity or fidelity, pixel/structural similarity, diversity or coverage, automated realism/no-reference quality, intensity/texture/image-statistical descriptors, reconstruction error, perceptual similarity, privacy/memorization similarity, and other automated image-quality metrics. The complete lists of approaches and metrics included in each of these groups are summarized in Table S5 and Table S6 in Multimedia Appendix 1, respectively.

Quantitative values (means, SDs, CIs, and sample sizes, where available) were recorded for each metric.

#### Impact on Model Performance

To assess the impact of synthetic images on downstream model performance, we extracted detailed information on model architectures and performance metrics from each study. Real-to-synthetic data ratios were extracted and classified by analytical purpose. Given their heterogeneous use across studies, these ratios were not pooled. For ratio-frequency summaries, only ratios related to downstream model development or evaluation were considered. When a study tested several ratios for the same analytical use, the ratio identified in the reported results as producing the best downstream performance was selected as the primary study-level ratio. This rule limited each study to one primary ratio per analytical use and prevented studies testing many ratios from contributing repeatedly to the compact frequency summaries; all tested ratios were retained in the study-level supplementary data.

Models were broadly categorized into deep learning and classical machine learning approaches. Among deep learning models, CNNs, with common variants including segmentation networks (eg, UNet [U-shaped convolutional neural network], UNet++, and Attention UNet), residual neural networks (eg, ResNet18 and ResNet50), inception-based architectures (eg, InceptionV3), densely connected convolutional network models (eg, DenseNet121), and standard feature extractors (eg, visual geometry group network [VGG16], MobileNet, and EfficientNet). GAN-based architectures, hybrid models, and, in rare cases, recurrent neural networks were also noted.

We documented the types of tasks addressed (eg, classification and segmentation) and extracted the reported performance metrics. For classification, these included accuracy, *F*_1_-score, recall (sensitivity), precision, specificity, and area under the receiver operating characteristic curve. For segmentation tasks, common metrics were the Dice similarity coefficient and Jaccard index. For each metric, we recorded available summary statistics, including mean values, SDs, CIs, and sample sizes. Explainability methods (eg, saliency-based maps) were also included in this section.

To facilitate comparative analysis, we stratified model performance by the dataset type used during training: original, traditionally augmented, synthetic-only, or synthetic-augmented. This stratification enabled the examination of performance trends across various imaging modalities and data augmentation strategies.

#### Explainability Methods

Because synthetic images may improve performance while also altering the visual features used by a model, we extracted explainability evidence as a separate assessment domain. Eligible methods included saliency and activation maps, gradient-weighted class activation mapping (Grad-CAM)/class activation mapping (CAM)–based approaches, attention mechanisms, feature-attribution methods, perturbation analyses, counterfactual explanations, and latent-space analyses. We recorded the purpose of each analysis (eg, inspection of clinically relevant regions, comparison of real and synthetic representations, artifact detection, class separability, or interpretation of model decisions) and noted whether outputs were clinically interpreted, expert-reviewed, or quantitatively validated against reference annotations. Conventional image-quality metrics and realism assessments were not considered evidence of explainability unless they explicitly evaluated model reasoning. For method-purpose summaries, all reported method-purpose combinations within each study were counted.

### Risk-of-Bias Assessment

Due to the lack of validated tools for assessing the risk of bias in studies on synthetic-image generation, we developed a set of criteria based on established reporting guidelines. These included the Strengthening the Reporting of Observational Studies in Epidemiology (STROBE) statement, the TRIPOD+AI (Transparent Reporting of a Multivariable Prediction Model for Individual Prognosis or Diagnosis+AI) extension for prediction model reporting [32,33], and selected domains from the ROBINS-I (Risk of Bias in Non-Randomized Studies of Interventions) tool [34] for evaluating bias in nonrandomized studies. Each study was assessed using the following predefined methodological domains: sampling strategy, population description, quality control, architecture description, synthetic-image generation and validation, outcome definition and reporting, and data completeness. For each domain, studies were categorized as having low concern, some concerns, or high concern based on these criteria. Table S7 in Multimedia Appendix 1 provides detailed scoring guidelines that specify the conditions for classifying studies into different risk levels.

Risk of bias was assessed independently by 2 reviewers. Interrater reliability before consensus was evaluated at the domain level using exact percentage agreement and Cohen κ. Because ratings were ordinal, quadratically weighted Cohen kappa was used as the primary reliability statistic, with unweighted and linearly weighted kappa calculated as sensitivity analyses. Ratings of “not applicable,” when present, were excluded from ordinal kappa calculations and reviewed separately.

Disagreements were resolved by consensus. Overall risk-of-bias judgments were assigned after consensus, ignoring not-applicable domains. Studies were classified as high concern if 2 or more domains were rated high concern, or if 1 high-concern domain co-occurred with 3 or more domains rated as having some concerns. Studies were classified as some concerns if they had 1 high-concern domain with less than 3 some-concern domains, or 3 or more some-concern domains and no high-concern domains. All remaining studies were classified as low concern.

### Synthesis Without Meta-Analysis

All data processing and visualization were performed in R (version 4.4.0; R Foundation for Statistical Computing). Results were synthesized descriptively and presented graphically, stratified by imaging modality. The synthesis was organized around the 2 review objectives: synthetic-image quality and downstream model performance. Image-quality evidence was first grouped as human-reader assessment or automated assessment and then by assessment task, metric family, specific metric, and imaging modality. Downstream evidence was grouped by training comparison (synthetic-only vs original/real or synthetic-augmented vs original/real), downstream task, harmonized performance metric, and imaging modality. These groupings separated outcomes with different constructs and directions of interpretation. The protocol specified the 2 broad objectives but not all detailed analytic subgroups; the detailed grouping scheme was developed during extraction and analysis in response to the observed outcome heterogeneity, without changing the review eligibility criteria or objectives.

During data extraction, we noted, for each study, whether effect estimates included both the sample size and a measure of variance (SD, SE, or CI). These details were missing or incomplete in more than 80% of the eligible studies. Together with heterogeneity in populations, imaging modalities, generative models, outcome definitions, and analytic settings, this prevented the calculation of comparable effect estimates and inverse-variance weights; meta-analysis was therefore considered inappropriate. We used a structured synthesis without meta-analysis, based on study-level tabulation, frequencies, and unweighted distributions of reported values or within-study changes. We did not conduct vote counting based on statistical significance and did not calculate a pooled effect estimate or pooled CI.

Metrics were standardized only when their constructs and scales were compatible. Human-reader rates and proportions were expressed on a 0% to 100% scale; AUC values were retained on a 0 to 1 scale; and Likert, ranking, agreement, and other scores remained on their original scales. Automated image-quality metrics were retained in their native units and summarized separately by metric; Structural Similarity Index Measure (SSIM) or Multiscale SSIM (MS-SSIM) values reported as percentages were divided by 100, and metrics were separated according to whether higher or lower values indicated better quality. For downstream performance, strict within-study percentage change was calculated as 100 × (target-arm value – original/real-arm value)/absolute original/real-arm value. The sign was reversed for lower-is-better metrics, so that positive values consistently indicated improved performance and negative values indicated worsening.

Downstream values were paired only when the study, harmonized metric, downstream task, aggregation level, class or subgroup, operating point, comparison context, generative model, downstream algorithm, and dataset matched. When duplicate source rows represented the same strict pairing unit and dataset arm, their reported means were averaged before pairing. Precomputed percentage-change outcomes were excluded from re-pairing to avoid calculating the same contrast twice. The paper-facing contrasts were restricted to synthetic-only versus original/real and synthetic-augmented versus original/real; synthetic-augmented versus synthetic-only was excluded because it addressed a different review question. For display, automated image-quality box plots required at least 7 studies and 7 eligible values per metric, while downstream metric plots required at least 20 strict paired-comparison data points within each contrast. These thresholds affected only the graphical display; all extracted results were retained in the supplementary tables of Multimedia Appendix 1 and in the public study-level dataset.

Potential heterogeneity was examined descriptively by comparing the direction, magnitude, and dispersion of findings across imaging modalities, clinical settings, generative-model families, image-quality assessment tasks, downstream tasks, dataset arms, and harmonized metrics. Risk-of-bias patterns and reporting completeness were considered when interpreting differences. Formal subgroup meta-analysis or meta-regression was not undertaken because comparable effect estimates and adequate uncertainty data were unavailable. Findings were presented in study-characteristic tables, frequency tables, bar plots, UpSet plots, heatmaps, and box plots showing medians, IQRs, and individual study-level data points.

## Results

### Study Selection and Characteristics

Our search identified 7302 records related to the use of synthetic images in human diagnosis, across PubMed, Scopus, IEEE Xplore, and Embase. After deduplication, screening, full-text assessment, and extraction and quality-control review, 160 studies met all eligibility criteria and were included in the systematic review (Figure 1). The main characteristics of the included studies are summarized in Table S8 in Multimedia Appendix 1.

**Figure 1.**
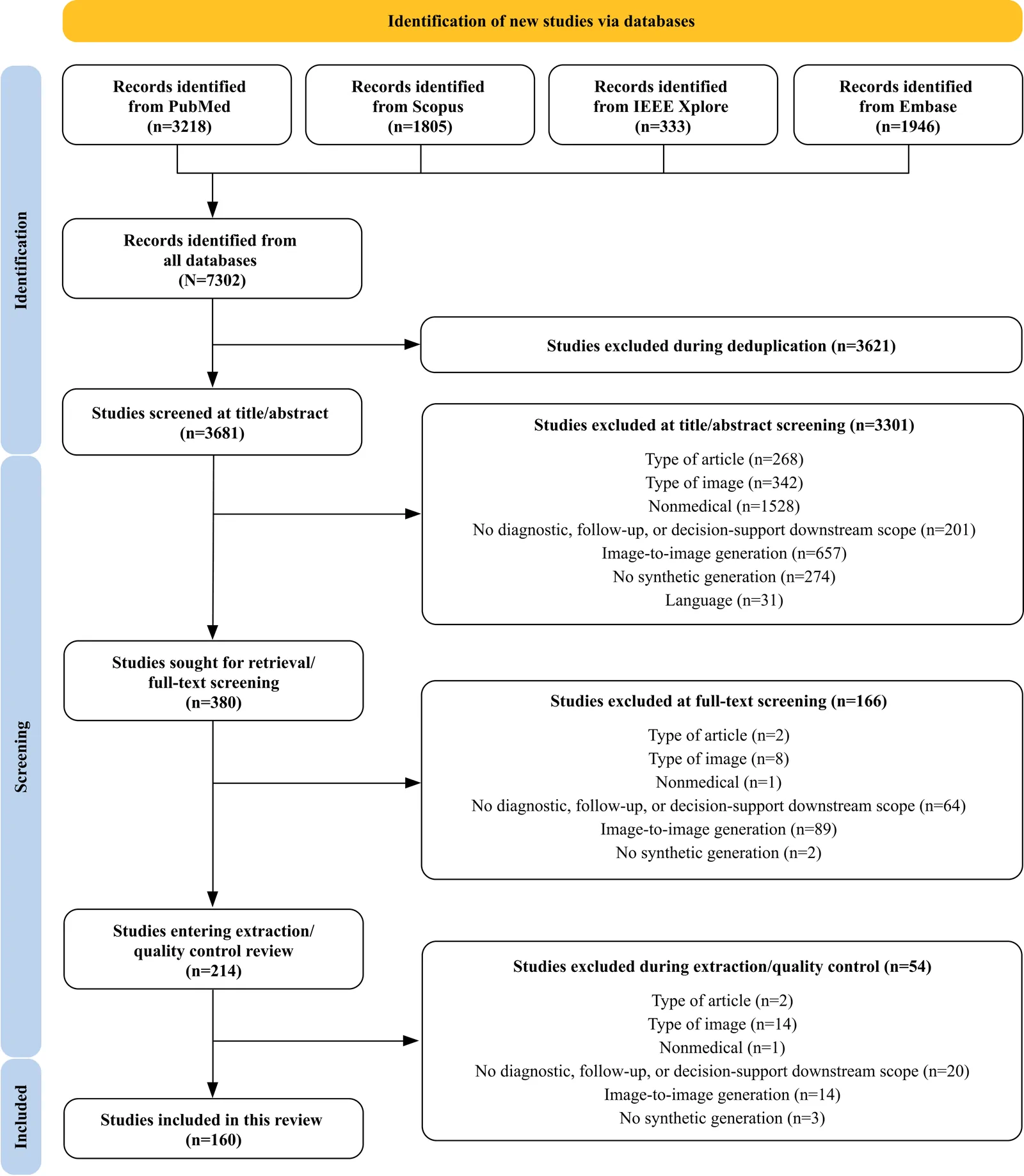
PRISMA (Preferred Reporting Items for Systematic Reviews and Meta‑Analyses) flow diagram summarizing study identification, screening, eligibility assessment, and inclusion in the systematic review.

As shown in Figure 2A, x-ray, magnetic resonance imaging (MRI), and computed tomography (CT) were the leading techniques used for synthetic-image generation. Grayscale was the most used color modality in these leading image modalities, as well as in ultrasound; it was followed by RGB (red, green, and blue), particularly used in clinical and fundus photography, dermoscopy, endoscopy, and optical coherence tomography (OCT). By contrast, positron-emission tomography (PET), coronary angioscopy, DaTscan, and intravascular ultrasound (IVUS), together accounted for only about 4% (6/160) of studies.

Figure 2B shows that the resolutions of the generated synthetic images varied considerably, with the most common size across studies clustering around 256×256 pixels, and a few ranging from as small as 32×32 to as large as 1024×1024.

Synthetic images were primarily applied in oncology, respiratory diseases, neurology, ophthalmology, and cardiovascular diseases. The bubble plot in Figure 2C further illustrates these associations, showing that MRI and CT were frequently used in oncology and neurology. In contrast, fundus imaging and optical coherence tomography images were mostly used in ophthalmology studies. Conversely, endocrinology, gynecology, nephrology, odontology, and otorhinolaryngology applications appeared in approximately 1% of studies, underscoring clinical areas in which the utility of synthetic images remains largely unexplored.

**Figure 2.**
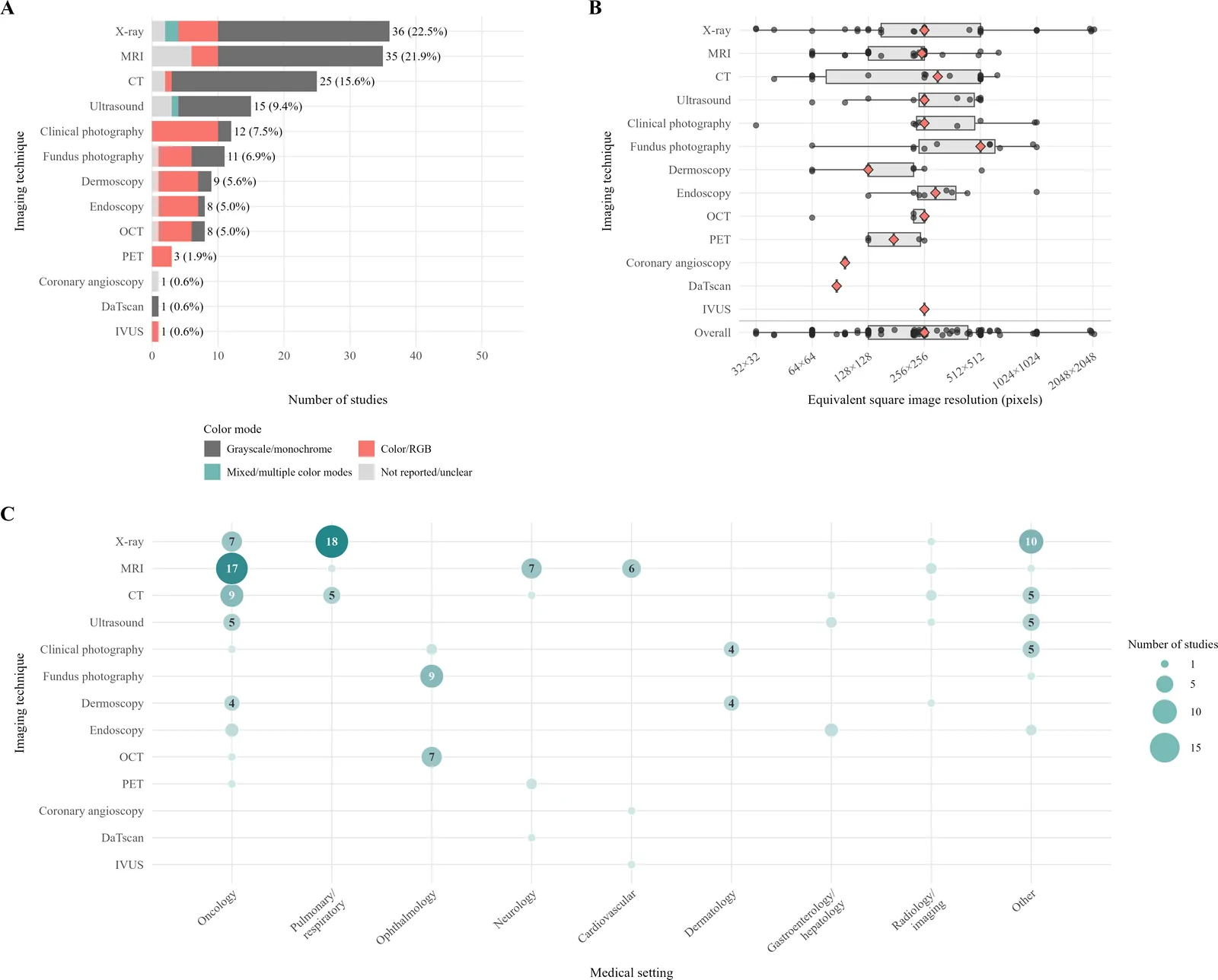
Overview of imaging techniques, color modes, image resolutions, and clinical settings in studies using synthetic images. (A) Number of studies by imaging technique and color mode; labels show n values and percentages for all 160 included studies. Studies reporting multiple imaging techniques contribute to each applicable technique. (B) Distribution of reported equivalent square image resolutions by imaging technique; points represent reported resolutions, boxes show the IQRs, and red diamonds show medians. The overall distribution is shown at the bottom. (C) Bubble plot showing imaging-technique and medical-setting co-occurrence. Bubble area represents the number of studies; medical settings with fewer than 5 studies are grouped as Other. CT: computed tomography; DaTscan: dopamine transporter scan; IVUS: intravascular ultrasound; MRI: magnetic resonance imaging; OCT: optical coherence tomography; PET: positron-emission tomography; RGB: red, green, and blue.

### Architectures for Image Generation

As shown in Figure 3A, GAN-based architectures remained the dominant generative model family and were reported in 160 (n=119, 74.4%) studies. This family included heterogeneous GAN subtypes rather than a single architecture, including deep convolutional generative adversarial network (DCGAN), StyleGAN, conditional or auxiliary-classifier GAN, PGGAN, Wasserstein GAN, CycleGAN, Pix2Pix, and other task-specific variants. Diffusion models were reported in 28 (17.5%) studies and became more visible in recent publication years. Hybrid, procedural/statistical, scientific/physical simulation, registration/deformation, VAE/autoencoder, and other neural synthesis approaches were less frequent. A detailed taxonomy and study-level architecture table are provided in Table S9 in Multimedia Appendix 1.

Across imaging modalities, GAN-based approaches were most commonly observed in MRI, CT, and x-ray studies, which were also the most frequently represented modalities overall (Figure 3C). They were also reported in less common modalities, such as PET, endoscopy, ultrasound, and photographic imaging. Less commonly used architecture families, including diffusion-based, hybrid, simulation-based, and VAE-based approaches, appeared across several imaging types, although with lower study counts.

**Figure 3.**
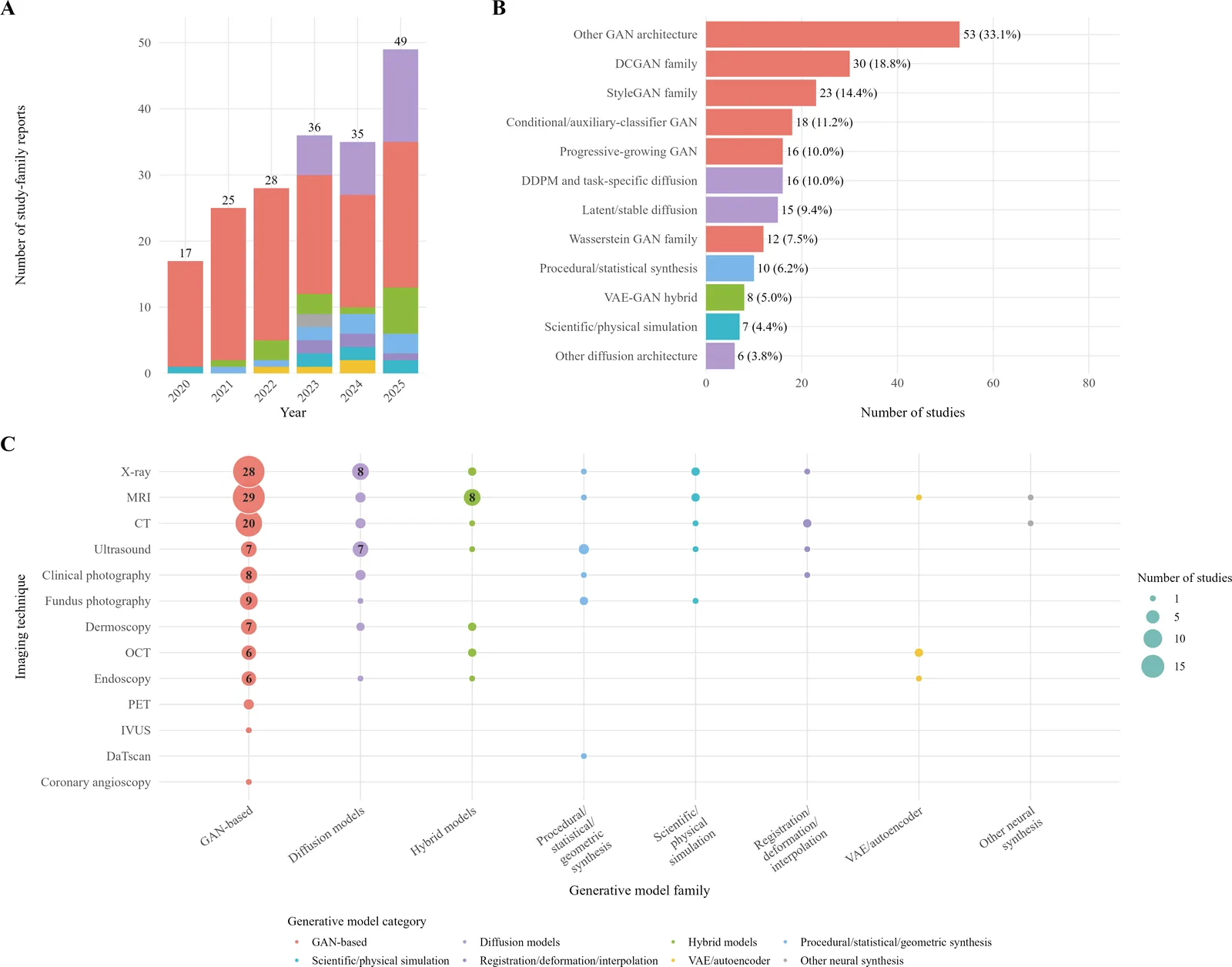
Generative model architectures used for synthetic medical image generation across the 160 included studies. (A) Study-family reports by publication year; categories are not mutually exclusive because a study can report more than one family. (B) Most frequently reported architecture subtypes, with n values and percentages for all 160 included studies. (C) Bubble plot showing co-occurrence between broad generative model families and imaging techniques. Bubble area represents the number of studies, and color identifies the broad model family. CT: computed tomography; DaTscan: dopamine transporter scan; DCGAN: deep convolutional generative adversarial network; DDPM: denoising diffusion probabilistic model; IVUS: intravascular ultrasound; GAN: generative adversarial network; MRI: magnetic resonance imaging; OCT: optical coherence tomography; PET: positron-emission tomography; StyleGAN: style-based generative adversarial network; VAE: variational autoencoder; VAE-GAN: variational autoencoder generative adversarial network.

### Image Quality

Overall, 160 (n=113, 70.6%) studies assessed synthetic-image quality using at least one human-reader or automated approach. Human-reader evidence was reported in 50 (31.2%) studies, automated image-quality assessment in 101 (63.1%) studies, and both approaches in 38 (23.8%) studies.

The most frequent task category among human-reader assessments was real-versus-synthetic discrimination or Turing-style assessment, followed by realism or image-quality ratings, qualitative expert review, diagnostic interpretation, feature identification or annotation, clinical plausibility or usefulness ratings, and preference or ranking tasks (Figure 4A). Most studies using human-reader approaches to assess image quality missed relevant methodological information (Figure 4B). At the metric level, the most frequently extracted end points were Turing-style discrimination metrics, followed by interrater agreement, authenticity rates, Likert or rating scores, and diagnostic performance metrics (Figure 4C). Quantitative rate/proportion end points that could be harmonized to a common 0% to 100% scale showed heterogeneous distributions across tasks and metrics (Figure 4D). A quantitative synthesis of tasks and metrics used for human-reader assessment of image quality is provided in Table S10 and Table S11 in Multimedia Appendix 1, respectively.

**Figure 4.**
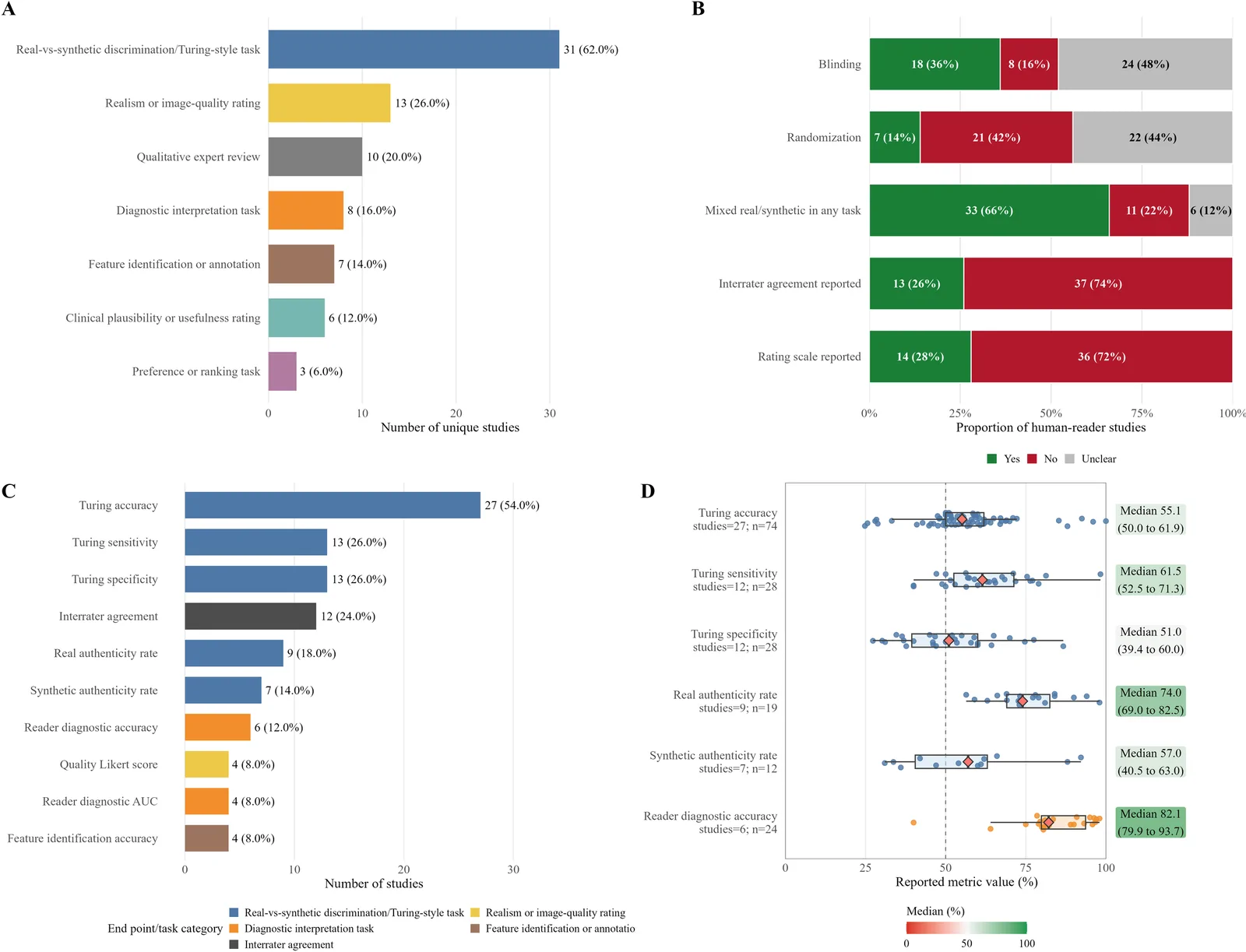
Human-reader study evidence and reporting completeness (50/160 studies). (A) Frequency of human-reader task categories, counted across unique reader studies. (B) Reporting completeness for key reader-study design items among the 50 studies with human-reader evidence. (C) Frequency of specific extracted human-reader study metrics. (D) Quantitative rate/proportion end points harmonized to a common 0% to 100% scale; studies could contribute more than one value when results were reported by reader group, image group, dataset, model, or end point. Categories are not mutually exclusive. Boxes represent the IQR (25th-75th percentile), vertical lines with orange diamonds indicate the median. Right-side labels show the median reported value and the IQR for each metric. AUC: area under the curve.

Figure 5 summarizes the use and results of automated metrics for assessing image quality. The most common trend among studies assessing image quality with automated metrics was the use of a single metric within either the distributional similarity or fidelity group (Figure 5A), primarily the Fréchet inception distance (FID) (Figure 5B). The quantitative synthesis analysis of automated metrics used for assessing image quality is summarized in Table S12 (metric groups) and Table S13 (specific metrics) in Multimedia Appendix 1.

Figure 5C-D show the distribution of reported values for the most frequently used automated image-quality metrics. Among metrics for which lower values indicate better image quality, FID showed substantial variability across studies, a pattern also observed for learned perceptual image patch similarity (LPIPS). Among metrics where higher values indicate better image quality, SSIM was the most frequently reported, followed by peak signal-to-noise ratio (PSNR) and inception score. Overall, the wide dispersion of values across metrics highlights the substantial heterogeneity of automated image-quality assessment and limits direct comparability across studies.

**Figure 5.**
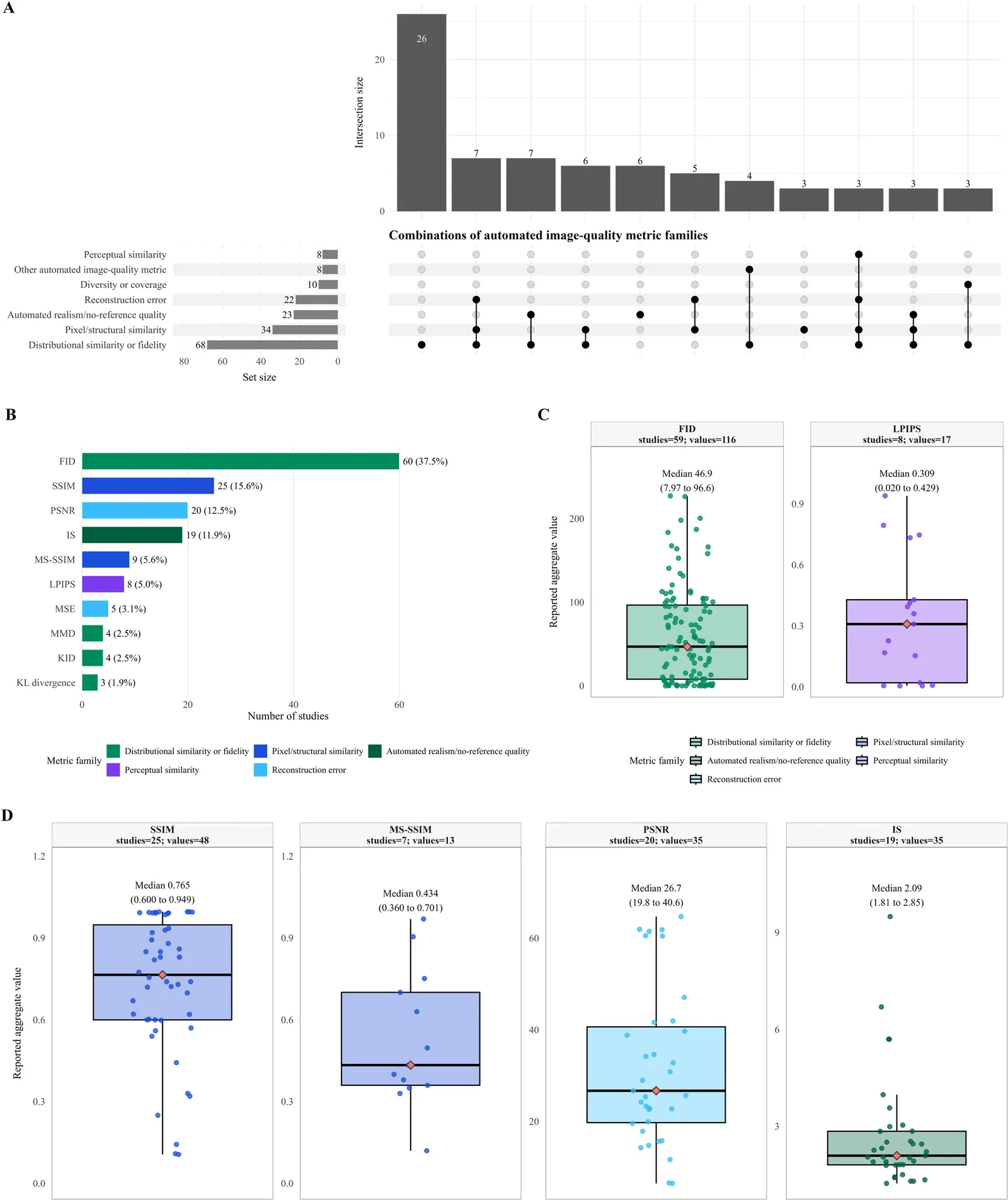
Summary of automated image-quality metrics used to assess synthetic images. (A) The UpSet plot illustrates combinations of automated quality assessment strategies reported across studies (≥3 studies). Each dot-and-line intersection indicates a specific combination of strategies used within the same study. Bar heights represent the number of studies using each combination. (B) Frequency of individual automated image-quality metrics (reported in ≥3 studies), grouped by overarching metric family. (C-D) Box plots of numeric values for higher‐count metrics (reported in ≥7 studies), separated according to whether lower values indicate better image quality (C) or higher values indicate better image quality (D). Boxes represent the IQR (25th-75th percentile), horizontal lines with orange diamonds indicate the median. FID: Fréchet inception distance; IS: inception score; KID: kernel inception distance; KL: Kullback-Leibler; LPIPS: learned perceptual image patch similarity; MMD: maximum mean discrepancy; MSE: mean squared error; MS-SSIM: Multiscale Structural Similarity Index Measure; PSNR: peak signal-to-noise ratio; SSIM: Structural Similarity Index Measure.

### Impact of Synthetic Data on Downstream Model Performance

Most studies (110/160, 68.8%) reported the real-to-synthetic data ratio; among these studies, balanced designs such as 1:1 were common, while several studies used synthetic-enriched configurations such as 1:2, 1:5, or 1:10; among studies with unbalanced designs, they tended to include more synthetic than real images (Figure S1, Multimedia Appendix 1). Detailed frequencies of ratios at the study level are provided in Figure S2 in Multimedia Appendix 1.

Figure 6 summarizes the downstream evaluation strategies and performance outcomes reported across studies. The most frequent dataset-arm pattern was the joint reporting of original/real and synthetic-augmented evidence, either alone or as part of studies that also included a synthetic-only arm. Synthetic-only evidence was less frequent and was reported either alone or in combination with original/real or with synthetic-augmented arms (Figure 6A).

**Figure 6.**
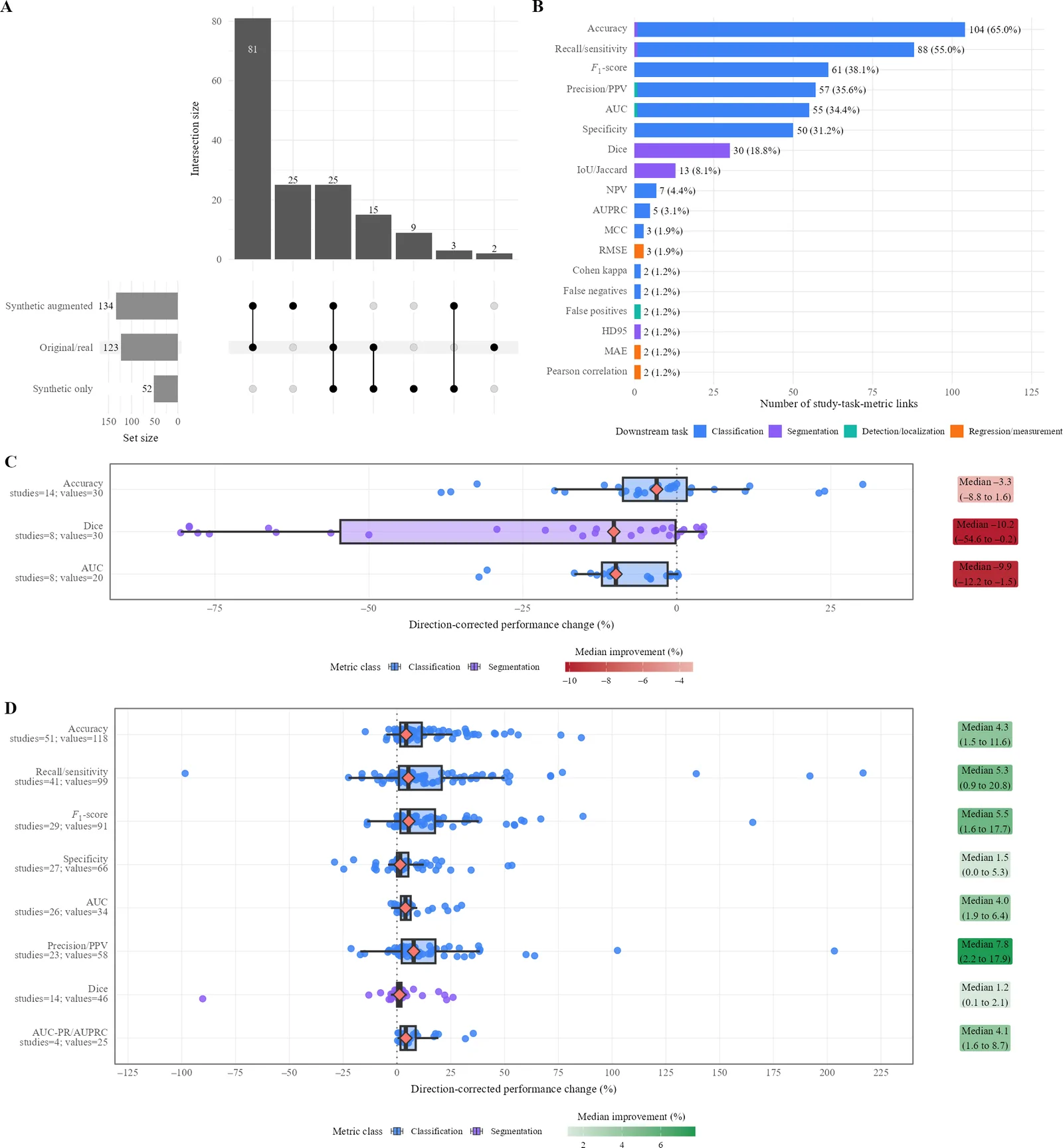
Downstream dataset-arm evidence, evaluation metrics, and performance changes associated with synthetic images. (A) The UpSet plot showing the study-level overlap of downstream dataset arms reported across studies, including original/real, synthetic-only, and synthetic-augmented arms. (B) Frequency of reported downstream evaluation metrics, counted as study-task-metric links and stacked by downstream task; metrics reported in at least 2 study-task combinations are shown. (C) Direction-corrected percentage change in downstream performance for synthetic-only vs original/real datasets. (D) Direction-corrected percentage change in downstream performance for synthetic-augmented vs original/real datasets. Positive values indicate improved performance relative to the original/real-data baseline. Plots show harmonized metrics with at least 20 strict paired comparison data points. Boxes represent the IQR (25th-75th percentile), vertical lines with orange diamonds indicate the median, and points represent individual paired comparison data points. Right-side labels show the median (Q1-Q3) improvement for each metric. AUC: area under the curve; AUC-PR/AUPRC: area under the precision-recall curve; IoU: intersection over union; MAE: mean absolute error; MCC: Matthews correlation coefficient; NPV: negative predictive value; PPV: positive predictive value; RMSE: root mean square error.

Classification was the predominant downstream task, and accuracy was by far the most frequently reported metric (104 study-task-metric links), followed by recall/sensitivity (88), *F*_1_-score (61), precision/positive predictive value (PPV) (57), area under the curve (AUC) (55), and specificity (50) (Figure 6B). Segmentation studies most frequently reported the Dice coefficient (30) and IoU/Jaccard index (13), whereas regression and localization outcomes were comparatively uncommon.

Across comparisons of synthetic-only versus original/real datasets, performance tended to decline for the plotted metrics, particularly the Dice coefficient and AUC, whereas accuracy showed a smaller median decrease (Figure 6C). In contrast, studies evaluating synthetic augmentation generally reported improved downstream performance relative to original/real datasets alone (Figure 6D). Median improvements were observed for most classification metrics, including precision/PPV (+7.8%), recall/sensitivity (+5.3%), *F*_1_-score (+5.5%), accuracy (+4.3%), area under the precision recall curve (AUPRC or AUC-PR)(+4.1%), and AUC (+4.0%), whereas specificity showed smaller gains (+1.5%). Improvements in segmentation performance were limited, with a median improvement in the Dice coefficient of only +1.2 %.

Explainability or model-interpretability evidence was reported in 68 of the 160 (42.5%) studies. The frequencies of the different methods, the validation of explanations, and their purposes are summarized in Figure S3 in Multimedia Appendix 1. Overall, latent-space or representation analyses were the most common approach, followed by Grad-CAM/CAM methods and saliency maps. Validation of explanation outputs was uncommon: only about half of the studies assessed clinical relevance, whereas quantitative validation against annotations, expert review, and explicit clinical expert involvement were rarely reported. Latent-space analyses were primarily used to examine representation structure and class separability, while Grad-CAM/CAM and saliency methods were mainly used to assess whether model attention aligned with clinically relevant regions. Overall, explainability was more often used as supportive evidence than as a formal validation of model behavior.

### Risk of Bias

Figure 7 summarizes the risk-of-bias assessment across 7 methodological domains: sampling strategy, source population description, image quality and preprocessing control, generative architecture reporting, generation-validation data separation, outcome definition and reporting, and data completeness and statistical reporting.

Before consensus, exact agreement across all risk-of-bias domain ratings was 55.1%, and the pooled, quadratically weighted Cohen κ was 0.322. Unweighted and linearly weighted κ values were 0.172 and 0.242, respectively. Domain-specific reliability estimates are provided in Table S14 in Multimedia Appendix 1.

After reaching consensus, 60 of the 160 (38%) included studies were classified as having low concern, 67 (42%) as having some concerns, and 33 (21%) as having high concern overall (Figure 7). The strongest methodological domains were generative architecture reporting (n=146, 91% low concern) and outcome definition and reporting (n=143, 89% low concern). In contrast, data completeness and statistical reporting represented the main methodological weaknesses, with only 16% (n=26) of studies rated as low concern and 49% (n=79) classified as high concern. Additional concerns were observed with source population description and generation-validation data separation. Individual study ratings and the full risk-of-bias matrix are provided in Figure S4 in Multimedia Appendix 1.

Reproducibility and openness reporting was heterogeneous across studies (Figure S5 in Multimedia Appendix 1). Evaluation protocols and training details were commonly reported, whereas source code, synthetic images, random seeds, model checkpoints, and pretrained models were rarely made available. Public datasets were more frequently accessible than generative-model artifacts. The most commonly used public/community datasets among included studies were International Skin Imaging Collaboration (ISIC) Archive, Lung Image Database Consortium—Image Database Resource Initiative (LIDC-IDRI), Alzheimer’s Disease Neuroimaging Initiative (ADNI), Brain Tumor Segmentation Challenge (BraTS), and Human Against Machine With 10,000 Training Images (HAM10000), while ImageNet was frequently used for model development or pretraining (Figure S6 in Multimedia Appendix 1). Multimedia Appendix 2 provides a complete catalog of datasets used in the reviewed studies.

We did not assign GRADE (Grading of Recommendations, Assessment, Development, and Evaluation) or another formal certainty rating because the evidence comprised heterogeneous AI development and validation studies rather than a common intervention-effect question. We therefore calibrated our interpretation according to the number and directness of contributing studies, consistency of findings, completeness of statistical reporting, availability of strict within-study comparisons, and risk of bias. Descriptive prevalence patterns supported by many studies were considered more robust than comparative performance findings, for which certainty remained limited.

**Figure 7.**
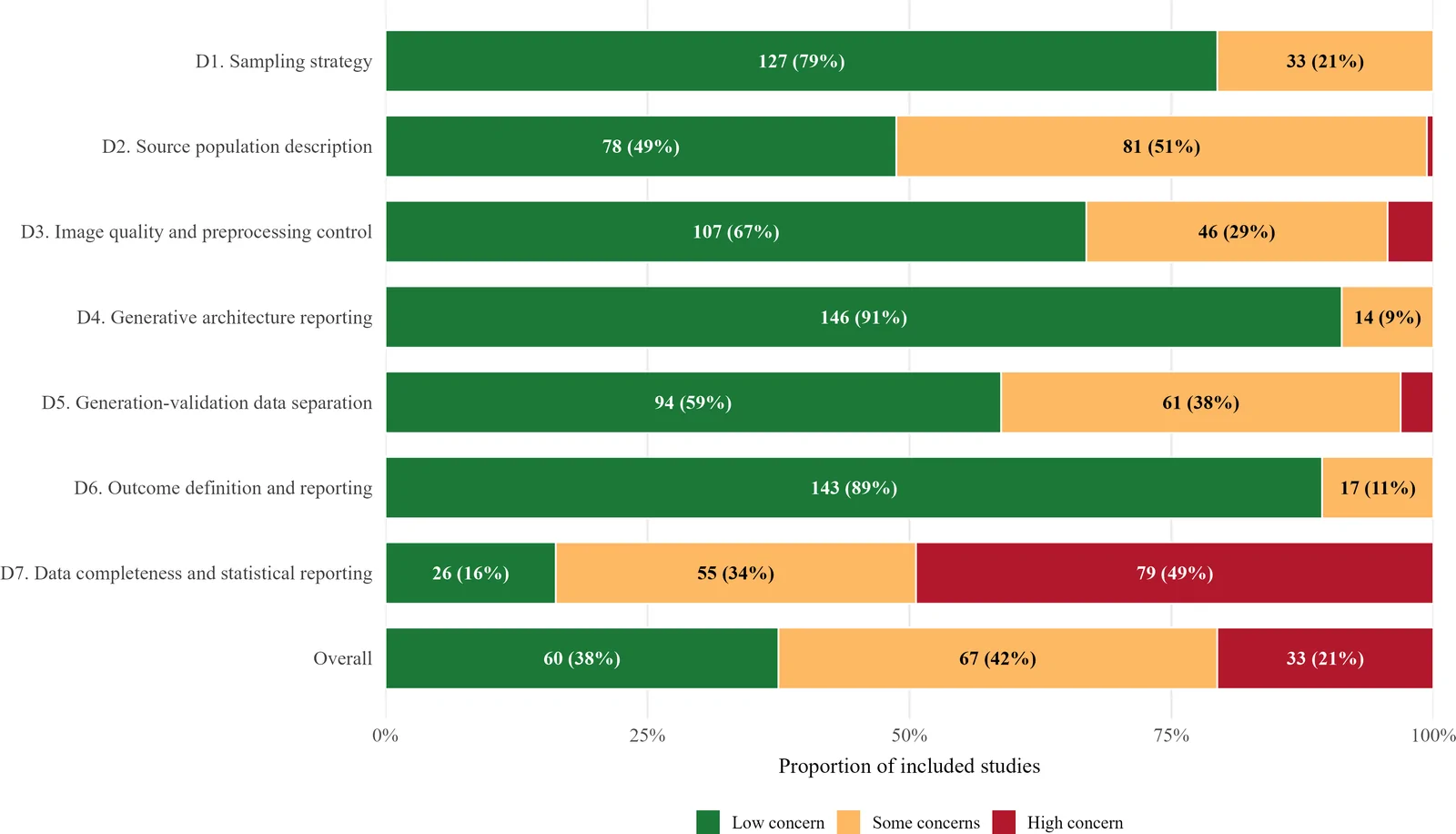
Risk-of-bias assessment across included studies on synthetic medical image generation and validation (N=160). Stacked horizontal bars show the proportion and number of studies classified as low concern, some concerns, or high concern for each of the 7 predefined methodological domains and for the overall judgment. The domains assessed included reporting of sampling strategy, source population characteristics, image quality and preprocessing control, generative architecture, separation between generation and validation data, outcome definition and reporting, and data completeness and statistical reporting. Final judgments were derived after consensus adjudication of reviewer disagreements.

## Discussion

### Principal Findings

Our systematic review of 160 studies reporting the generation of synthetic images and their adequacy or quality in the context of diagnostic imaging reveals both the promise and current limitations of this rapidly evolving technology in health care. The findings highlight significant heterogeneity in methodological approaches, evaluation strategies, and performance outcomes that collectively point to an urgent need for standardization in this field.

Grayscale-based imaging modalities, particularly MRI, x-ray, and CT, were the most frequently represented techniques for synthetic-image generation, especially in oncology, respiratory medicine, and neurology. However, our review was not designed to determine the reasons for this modality imbalance. Future studies should examine whether it reflects differences in data availability, the maturity of established AI workflows, or modality-specific challenges in image generation and validation. Photograph-based diagnostic modalities, including dermoscopy, endoscopy, fundus photography, and other clinical photography applications, were comparatively underrepresented. These modalities therefore remain important areas for future research, particularly because image color, texture, illumination, and fine surface features may require evaluation approaches that differ from those used for cross-sectional or projection imaging.

Regarding generative architecture, GAN-based methods remained dominant and were reported in 119 of the 160 included studies. However, this family comprised a heterogeneous array of architectures, including DCGANs, StyleGANs, conditional GANs, PGGANs, Wasserstein variants, CycleGANs, Pix2Pix models, and hybrid approaches. Diffusion models were less frequent overall but became increasingly represented in recent years, including applications in controllable 3D image generation, text-guided CT synthesis, pathology-conditioned generation, low-dose PET reconstruction, and fairness-aware synthesis [35-39]. These developments suggest that the field is evolving toward a more diverse ecosystem of generative approaches, although evaluation methods have not developed at the same pace.

One of the most important findings was the heterogeneity in synthetic-image quality assessment. Overall, 113 studies assessed image quality using at least one human-reader or automated approach. Human-reader evidence was reported in 50 studies, automated assessments in 101, and 38 studies combined both approaches.

Automated assessments included distributional metrics such as FID, maximum mean discrepancy, and kernel inception distance; structural metrics such as SSIM and PSNR; no-reference measures such as inception score; perceptual metrics such as LPIPS; and measures of reconstruction error, diversity, texture, and intensity. Differences in the constructs, directions, and reported values of these metrics limited direct comparisons across studies. As no single metric captures all clinically relevant dimensions of quality, evaluation should combine distributional fidelity, diversity, preservation of anatomical and pathological features, detection of artifacts or memorization, task-based utility, and structured human-reader assessment.

Recently proposed frameworks, including the Synthetic Medical Data Scorecard and the Clinical Evaluation of Medical Image Synthesis protocol, provide useful starting points for developing more consistent and clinically meaningful evaluation practices [40,41]. Our analysis also showed that many human-reader assessments lacked essential methodological information. Future studies should report reader expertise, blinding, randomized or shuffled image presentation, rating-scale definitions, and interrater agreement, in line with broader reporting recommendations for medical-imaging AI [40,42].

The downstream-performance synthesis showed even greater heterogeneity because studies differed in clinical tasks, generative and downstream architectures, dataset composition, real-to-synthetic ratio, comparators, operating points, and reported metrics. Several studies lacked a clearly matched original-data baseline, limiting assessment of the isolated contribution of synthetic data. In strict within-study comparisons, models trained exclusively on synthetic images generally performed worse than those trained on real images, particularly for AUC and the Dice coefficient. These findings suggest that synthetic images cannot currently be assumed to be an interchangeable replacement for real training data.

By contrast, adding synthetic images to real training datasets was associated with modest median improvements in most classification metrics, including precision, recall, *F*_1_-score, accuracy, AUC, and AUPRC. Improvements in specificity were smaller, and the median improvement in the Dice coefficient was only 1.2%. The smaller gains in segmentation may reflect the need to preserve precise image-label correspondence, anatomical structure, and lesion boundaries, although this interpretation should be evaluated directly in future studies. Importantly, these changes are unweighted descriptive summaries rather than pooled causal effects and should not be interpreted as evidence that synthetic augmentation will improve performance in every modality, population, or clinical setting.

Despite growing interest in explainable AI, fewer than half of the studies used explainability methods. Latent-space and representation analyses were most common, followed by Grad-CAM, CAM, and other saliency-based methods. Formal validation against reference annotations, quantitative localization measures, or expert assessment was uncommon. Previous medical-imaging research has shown that commonly used saliency methods may have limited localization utility, repeatability, and reproducibility [43]. Explainability outputs should therefore not be considered reliable evidence of clinically appropriate model behavior without quantitative or expert validation.

Beyond performance and explainability, safety, privacy, and governance should also be considered. Although synthetic data are often presented as a privacy-preserving alternative to real patient data, generative models may memorize training examples or reveal information through nearest-neighbor similarity and membership-inference attacks [44,45]. The provenance, consent basis, licensing, intended use, population representation, and limitations of the source data should therefore be clearly documented. Structured tools such as model cards, datasheets for datasets, and the STANDING Together recommendations may improve transparency regarding model performance, dataset provenance, intended use, limitations, and potential risks [46-48]. Explicit disclosure is also needed when synthetic images are used for model development, training, or evaluation.

Because no widely accepted tool is available for assessing risk of bias in synthetic-image generation studies, our criteria were pragmatically derived from relevant reporting guidelines, including STROBE and TRIPOD+AI [32,33], and should be interpreted as a structured methodological appraisal rather than a validated measurement instrument. After consensus, 60 studies were classified as having low overall concern, 67 as having some concerns, and 33 as having high concern. Generative architecture reporting and outcome definition and reporting were the strongest domains, with 91% and 89% of studies, respectively, rated as low concern. In contrast, data completeness and statistical reporting represented the main methodological weaknesses, with only 16% rated as low concern and 49% as high concern. Additional concerns were observed for source population description and generation-validation data separation. Incomplete reporting of means, variance measures, sample sizes, and pairing information prevented inverse-variance weighting and formal meta-analysis.

Consequently, the reported medians and IQRs give each eligible study-level data point equal weight, do not account for differences in precision or sample size, and should not be interpreted as pooled or causal effects. A study could also contribute more than one data point when it reported distinct eligible tasks, datasets, models, classes, or operating points. Future research should report baseline performance, uncertainty estimates, sample sizes, and sufficient pairing information to enable more robust synthesis.

The synthesis without meta-analysis has additional limitations. Detailed synthesis groupings were refined after data extraction, and alternative grouping choices might produce different patterns. Selecting the best-performing real-to-synthetic ratio for compact frequency summaries may overstate favorable performance, although all tested ratios remain available in the study-level data. Minimum-count thresholds excluded sparsely reported metrics from the main figures, formal certainty ratings were not assigned, and multiple data points from individual studies may not be statistically independent. These factors limit the strength and generalizability of the conclusions.

In summary, our findings indicate that synthetic images may help address data scarcity and imbalance, but their benefits cannot be universally assumed. Synthetic-only training generally underperformed real-data training, whereas augmentation was associated with modest classification improvements and smaller segmentation gains. Harmonized criteria and standardized methodologies are therefore needed to assess image quality, downstream utility, privacy, and reporting completeness. A consensus reporting guideline, potentially developed in alignment with the EQUATOR Network, would help improve the validity, reproducibility, and comparability of studies in this field [49].

This review has several limitations. The rapid evolution of generative AI means that new architectures and applications may have emerged after the prespecified publication period. We included only peer-reviewed articles and excluded preprints, workshops, and conference proceedings. Although this may have omitted recent developments, it reduced publication-related heterogeneity and ensured that sufficient information was available for extraction and risk-of-bias assessment. We also excluded microscopic and histopathologic imaging and image-to-image transformation studies, limiting generalizability to these areas.

Finally, the search used a focused set of title- and abstract-based free-text terms rather than a PICO (Population-Intervention-Comparator-Outcome)–structured framework. This reflected the broad methodological nature of the review, which covered multiple specialties, modalities, generative architectures, downstream applications, and outcomes. Nevertheless, this approach may have missed studies that did not explicitly describe synthetic-image generation in their titles or abstracts.

### Conclusions

Our systematic review provides a comprehensive quantitative assessment of synthetic-image quality evaluation and downstream model impact across diagnostic medical imaging studies. Unlike previous reviews that focused on specific diseases, modalities, or generative architectures, it jointly examines image quality, downstream performance, explainability, reproducibility, and methodological quality across imaging domains.

Synthetic images show promise for addressing data scarcity and imbalance, particularly when used to complement real training datasets. However, synthetic-only training generally provides limited value, whereas synthetic augmentation is associated with modest improvements in classification performance and smaller gains in segmentation. Standardized multimethod evaluation, privacy assessment, transparent reporting, and rigorous external validation are needed to support the reliable translation of synthetic-image technologies into clinical AI applications.

## Supplementary material

10.2196/81472 Multimedia Appendix 1 Methods and results (figures and tables), including search strategies, methodological definitions and taxonomies, risk-of-bias criteria, study characteristics, quantitative summaries, and supplementary figures on downstream data composition, explainability, risk of bias, reproducibility, and dataset use.

10.2196/81472 Multimedia Appendix 2 Study-level data, including human-reader assessment evidence, a catalog of datasets used across the included studies, and detailed risk-of-bias assessment and reviewer-agreement records.

10.2196/81472 Checklist 1 PRISMA 2020, PRISMA-S, and SWiM checklists.

## Abbreviations

ADNI: Alzheimer’s Disease Neuroimaging Initiative
AUPRC/AUC-PR: area under the precision-recall curve
BigGAN: large-scale generative adversarial network
BraTS: Brain Tumor Segmentation Challenge
CAM: class activation mapping
CNN: convolutional neural network
CT: computed tomography
DCGAN: deep convolutional generative adversarial network
FID: Fréchet inception distance
GAN: generative adversarial network
Grad-CAM: gradient-weighted class activation mapping
GRADE: Grading of Recommendations, Assessment, Development, and Evaluation
HAM10000: Human Against Machine With 10,000 Training Images
ISIC: International Skin Imaging Collaboration
LIDC-IDRI: Lung Image Database Consortium—Image Database Resource Initiative
LPIPS: learned perceptual image patch similarity
LSGAN: least squares generative adversarial network
MRI: magnetic resonance imaging
MS-SSIM: Multiscale Structural Similarity Index Measure
PET: positron-emission tomography
PGGAN: progressive-growing generative adversarial network
PICO: Population-Intervention-Comparator-Outcome
PPV: positive predictive value
PRISMA: Preferred Reporting Items for Systematic Reviews and Meta‑Analyses
PRISMA-S: Preferred Reporting Items for Systematic Reviews and Meta‑Analyses—Search Extension
PROSPERO: International Prospective Register of Systematic Reviews
PSNR: peak signal-to-noise ratio
RGB: red, green, and blue
ROBINS-I: Risk of Bias in Non-Randomized Studies of Interventions
SAGAN: Self-Attention Generative Adversarial Network
SSIM: structural similarity index measure
SSIM: Structural Similarity Index Measure
STROBE: Strengthening the Reporting of Observational Studies in Epidemiology
StyleGAN: style-based generative adversarial network
SWiM: Synthesis Without Meta-Analysis
TRIPOD+AI: Transparent Reporting of a Multivariable Prediction Model for Individual Prognosis or Diagnosis+AI
UNet: U-shaped convolutional neural network
VAE: variational autoencoder
VAE-GAN: variational autoencoder generative adversarial network
VGG: visual geometry group network
