# Supplementary material for: Quality Assessment and Model Training Impact of Synthetic Images in Medical Diagnostic Imaging: Systematic Review
Source: JMIR AI. 2026 Oct 15;5:e81472. doi: 10.2196/81472 (PMC13671675; doi:10.2196/81472)
Supplement: Supplementary file 1 [file ai-v5-e81472-s001.docx]

Synthetic images in diagnostic imaging: a systematic review of quality assessment and model training impact – Multimedia appendix 1

Contents

[Supplementary Methods	3](#_Toc235448320)

[PRISMA-S	3](#_Toc235448321)

[Search strategy	3](#_Toc235448322)

[Search limits, restrictions, and filters	4](#_Toc235448323)

[Search results by source	4](#_Toc235448324)

[Record management and deduplication	5](#_Toc235448325)

[Table S1. Canonical taxonomy of generation model families and subtypes used in the review.	6](#_Toc235448326)

[Table S2. Human reader task categories	9](#_Toc235448327)

[Table S3. Canonical human-reader metrics and coding definitions.	11](#_Toc235448328)

[Table S4. Interpretation and minimum reporting guidance for canonical human-reader endpoint/task categories.	14](#_Toc235448329)

[Table S5. Automated image-quality metric families.	17](#_Toc235448330)

[Table S6. Canonical definitions of automated image-quality metrics reported in the review.	19](#_Toc235448331)

[Table S7. Risk of bias criteria and assessment levels.	26](#_Toc235448332)

[Supplementary results	28](#_Toc235448333)

[Table S8. Overview of the 160 included studies on synthetic medical imaging.	29](#_Toc235448334)

[Table S9. Frequency of canonical generation model families and subtypes across the 160 included studies.	47](#_Toc235448335)

[Table S10. Frequency of canonical human-reader endpoint/task categories.	49](#_Toc235448336)

[Table S11. Frequency and quantitative summary of canonical human-reader metrics.	50](#_Toc235448337)

[Table S12. Frequency of canonical automated image-quality metric families.	52](#_Toc235448338)

[Table S13. Frequency and quantitative summary of canonical automated image-quality metrics.	53](#_Toc235448339)

[Figure S1. Study-level composition of primary downstream real-to-synthetic ratios.	57](#_Toc235448340)

[Figure S2. Downstream-relevant real-to-synthetic ratios by study and analytical use.	58](#_Toc235448341)

[Figure S3. Explainability and model-interpretability evidence.	60](#_Toc235448342)

[Table S14. Inter-rater reliability statistics before risk-of-bias consensus.	61](#_Toc235448343)

[Figure S4. Final study-level risk-of-bias assessment across included studies.	63](#_Toc235448344)

[Figure S5. Reproducibility and openness indicators across included studies.	64](#_Toc235448345)

[Figure S6. Most frequently used public or community datasets across included studies.	65](#_Toc235448346)

[References	69](#_Toc235448347)

# Supplementary Methods

## PRISMA-S

### Search strategy

The search was designed to identify studies reporting the generation, validation, or downstream use of synthetic medical images. The main concepts covered by the search strategy were: synthetic images or synthetic data generation; medical, diagnostic, or clinical imaging; and artificial intelligence or machine learning–based image generation methods.

PubMed/MEDLINE and Scopus were searched on November 28, 2025. IEE Xplore was added on December 8, 2025, and finally Embase was searched on July 9, 2026. No study registries were searched because the review focused on published studies of synthetic image generation and downstream model evaluation rather than prospective clinical trials.

Search terms were developed to capture three main concepts:

1. Synthetic image generation and synthetic data.
2. Medical or diagnostic imaging.
3. Artificial intelligence, machine learning, deep learning, or generative models.

Terms were adapted to the syntax and indexing structure of each database. Free-text terms were used to capture emerging terminology in synthetic image generation and generative artificial intelligence.

Articles not containing the search terms either in the title or abstract were expected to be non-relevant for the study purpose; therefore, we forced them to be present either in title or the abstract. Searches were conducted as follows:

#### PubMed/MEDLINE

("synthetic image*"[tiab] OR "image synthesis"[tiab] OR "image generation"[tiab] OR "generated image"[tiab] OR "artificial image"[tiab]) AND ((fft[Filter]) AND (2020/1/1:2025/9/30[pdat]) AND (english[Filter]))

#### Scopus

( TITLE-ABS ( "synthetic image*" ) OR TITLE-ABS ( "image generation" ) OR TITLE-ABS ( "image synthesis" ) OR TITLE-ABS ( "generated image" ) OR TITLE-ABS ( "artificial image" ) ) AND PUBYEAR > 2019 AND ( PUBYEAR < 2025 OR PUBDATETXT ( "January 2025 or February 2025 or March 2025 or April 2025 or May 2025 or June 2025 or July 2025 or August 2025 or September 2025" ) ) AND SUBJAREA ( MEDI OR NURS OR HEAL OR MULT )

#### IEEE explorer

( ("Document Title":"synthetic image*") OR ("Document Title":"image synthesis") OR ("Document Title":"image generation") OR ("Document Title":"generated image") OR ("Document Title":"artificial image") OR ("Abstract":"synthetic image*") OR ("Abstract":"image synthesis") OR ("Abstract":"image generation") OR ("Abstract":"generated image") OR ("Abstract":"artificial image") ) AND ("All Metadata":med*)

#### Embase

('synthetic image*':ab,ti OR 'image synthesis':ab,ti OR 'image generation':ab,ti OR 'generated image':ab,ti OR 'artificial image':ab,ti) AND english:la AND [2020-2025]/py AND ([article]/lim OR [article in press]/lim) NOT ([review]/lim OR [conference abstract]/lim OR [conference review]/lim OR [editorial]/lim OR [letter]/lim OR [note]/lim OR [erratum]/lim OR [book]/lim)

### Search limits, restrictions, and filters

| Database | Limits or restrictions applied | Rationale |
| --- | --- | --- |
| PubMed/MEDLINE | Fft[Filter] | To identify studies for which the full text could be accessed online. |
|  | English[Filter] | Due to resource limitations for translations and data extraction. |
| Scopus | SUBJAREA ( MEDI OR NURS OR HEAL OR MULT ) | To focus the research on health and medicine related disciplines and improve relevance. |
| IEEE Xplore | Content type: Journals. | To exclude conference proceedings, workshops and other non-journal publications. |
|  | Year range 2020 - 2025 | Filter by added date to capture recent developments. |
| Embase | [2020-2025]/py | Filter by published year |

### Search results by source

| Source | Date searched | Records retrieved | Records after deduplication |
| --- | --- | --- | --- |
| PubMed/MEDLINE | 28/11/2025 | 3,218 | 2,266 |
| Scopus | 28/11/2025 | 1,805 | 945 |
| IEEE Xplore | 08/12/2025 | 333 | 188 |
| Embase | 09/07/2026 | 1,946 | 282 |
| Total | — | 7,302 | 3,681 |

No validated methodological search filter was applied unless specified above. Search strings were designed to maximize sensitivity for studies involving synthetic medical image generation and downstream image-based artificial intelligence applications.

### Record management and deduplication

All records retrieved from the database searches were exported to Excel for record management and deduplication.

Deduplication was performed with DOI. After automatic deduplication, remaining potential duplicates were checked manually using combinations of title, DOI, authors, journal, and publication year.

The number of records retrieved before deduplication was 7,302. The number of duplicate records removed was 3,621. The number of records retained for screening after deduplication was 3,681.

## Table S1. Canonical taxonomy of generation model families and subtypes used in the review.

Architectures were coded using eight mutually interpretable generation model families and 20 canonical subtypes. Reported studies may contribute to more than one family or subtype when multiple models were evaluated. All percentages use the 160 included studies as the denominator. Table S9 reports the corresponding frequencies; study-specific model wording is retained in Table S8 for auditability.

| Generation model family | Canonical model subtype | Coding rule / definition | Representative models or wording |
| --- | --- | --- | --- |
| **GAN-based** | **Other GAN architecture** | GAN architectures not covered by a more specific canonical GAN subtype. | GAN; study-specific GAN variants |
|  | **DCGAN family** | Deep-convolutional GAN architectures and close variants. | DCGAN; 3D-DCGAN |
|  | **StyleGAN family** | Style-based generators controlling synthesis through a learned latent style space. | StyleGAN; StyleGAN2; StyleGAN3 |
|  | **Progressive-growing GAN** | GANs trained by progressively increasing image resolution. | PGGAN; PG-GAN |
|  | **Conditional / auxiliary-classifier GAN** | GANs conditioned on labels, masks, clinical variables, or auxiliary classification objectives. | CGAN; ACGAN |
|  | **Wasserstein GAN family** | GANs using Wasserstein objectives, including gradient-penalty variants. | WGAN; WGAN-GP |
|  | **Pix2Pix family** | Paired image-to-image translation GANs. | Pix2Pix; Pix2PixHD |
|  | **CycleGAN family** | Unpaired image-to-image translation using cycle consistency. | CycleGAN |
| **Diffusion models** | **Latent / Stable Diffusion** | Diffusion performed in a learned latent space, including Stable Diffusion variants. | Latent diffusion model; Stable Diffusion |
|  | **DDPM and task-specific diffusion** | Denoising diffusion probabilistic models and task-adapted diffusion architectures. | DDPM; conditional DDPM |
|  | **Other diffusion architecture** | Diffusion architectures not covered by the canonical latent, DDPM, or score-based subtypes. | Text-guided or study-specific diffusion |
|  | **Score-based diffusion** | Score-matching generative models using stochastic or deterministic sampling dynamics. | Score-based generative model |
| **Hybrid models** | **VAE-GAN hybrid** | Architecture combining variational autoencoding with adversarial training. | VAE-GAN; AL-VAE |
|  | **Diffusion-GAN / VQGAN hybrid** | Architectures combining diffusion with GAN or vector-quantized generative components. | VQGAN; diffusion-GAN hybrid |
|  | **Other hybrid architecture** | Hybrid generative architectures not covered by canonical hybrid subtypes. | Study-specific hybrid models |
| **Procedural / statistical / geometric synthesis** | **Procedural / statistical synthesis** | Rule-based, statistical, geometric, or analytic synthesis without a primary neural generator. | Statistical modelling; procedural synthesis |
| **Scientific / physical simulation** | **Scientific / physical simulation** | Synthesis-based on anatomical, physical, acquisition, or numerical simulation. | Numerical phantom; physics-based simulation |
| **Registration / deformation / interpolation** | **Registration / deformation / interpolation** | Generation using spatial transformation, deformation fields, or interpolation between existing images. | Registration; deformation; interpolation |
| **VAE / autoencoder** | **VAE / autoencoder** | Variational autoencoders or related autoencoder-based generative models without a primary GAN component. | VAE; autoencoder |
| **Other neural synthesis** | **Other neural synthesis** | Neural synthesis methods not assigned to the other canonical model families. | Study-specific neural synthesis |

## Table S2. Human reader task categories

| **Human reader endpoint/task category** | **Definition / coding rule** | **Examples of included tasks or wording** |
| --- | --- | --- |
| **Real-vs-synthetic discrimination / Turing-style task** | Readers were asked to distinguish real from synthetic images, classify images as real or generated, or judge whether synthetic images appeared authentic. | Turing test; visual Turing test; real-versus-synthetic classification; authenticity judgement; fooling task. |
| **Realism or image-quality rating** | Readers rated perceived realism, visual quality, image quality, artefacts, or overall appearance using a structured scale. | Likert realism score; image-quality score; visual-quality rating; artefact rating; realism rating by experts. |
| **Clinical plausibility or usefulness rating** | Readers judged whether synthetic images were clinically plausible, acceptable, useful, or representative of real clinical images. | Clinical plausibility assessment; clinical usefulness rating; acceptability judgement; realism of pathological findings. |
| **Preference or ranking task** | Readers selected, ranked, or compared images, models, or image sets according to realism, quality, preference, or suitability. | Pairwise preference; best-image choice; ranking of models; preferred image selection. |
| **Diagnostic interpretation task** | Readers performed a clinically meaningful diagnostic or classification task using real and/or synthetic images. | Diagnosis; disease classification; lesion detection; benign/malignant classification; clinical interpretation. |
| **Feature identification or annotation** | Readers identified, annotated, or assessed anatomical structures, lesions, pathological findings, or radiological features. | Feature recognition; landmark identification; lesion annotation; radiological feature assessment; pathology feature identification. |
| **Text-image matching** | Readers matched images to prompts, captions, reports, clinical descriptions, or text labels. | Matching a generated image to a text prompt; selecting the correct clinical description; image-caption matching. |
| **Qualitative expert review** | Experts provided a narrative, qualitative, or non-numeric judgement of image realism, quality, plausibility, or clinical appearance. | Expert visual review; clinician inspection; qualitative assessment; narrative judgement that images were realistic or clinically acceptable. |
| **Interrater agreement** | The study reported agreement between readers for any human reader task or endpoint. | Agreement between radiologists, clinicians, or expert reviewers; concordance across raters. |
| **Other structured reader endpoint** | Human reader endpoint that was structured but could not be mapped to the predefined categories above. | Study-specific reader score, non-standard judgement, or unclear structured endpoint. |

## Table S3. Canonical human-reader metrics and coding definitions.

| Human-reader endpoint/task category | Canonical metric | Definition / coding rule | Typical unit or scale | Interpretation note |
| --- | --- | --- | --- | --- |
| **Real-vs-synthetic discrimination / Turing-style task** | **Turing accuracy** | Accuracy with which readers correctly distinguish real from synthetic images. | % or proportion | In balanced Turing-style tasks, values closer to chance may indicate higher perceptual realism of synthetic images. |
|  | **Turing sensitivity** | Sensitivity reported in a real-versus-synthetic discrimination task. | % or proportion | Direction depends on which class was defined as the positive class. |
|  | **Turing specificity** | Specificity reported in a real-versus-synthetic discrimination task. | % or proportion | Direction depends on which class was defined as the negative class. |
|  | **Turing AUC** | Area under the ROC curve for real-versus-synthetic discrimination. | AUC | Values closer to chance may indicate higher realism in balanced discrimination tasks. |
|  | **Synthetic authenticity rate** | Proportion of synthetic images judged as real or authentic by readers. | % or proportion | Higher values indicate that synthetic images were more often perceived as real. |
|  | **Real authenticity rate** | Proportion of real images judged as real or authentic by readers. | % or proportion | Higher values indicate correct recognition of real images. |
|  | **Fooling rate** | Proportion of cases in which readers were fooled by synthetic images. | % or proportion | Higher values indicate greater perceptual realism of synthetic images. |
| **Realism or image-quality rating** | **Realism Likert score** | Reader rating of perceived image realism using an ordinal or numeric scale. | Likert or numeric scale | Usually, higher values indicate greater realism, but the original scale direction should be checked. |
|  | **Quality Likert score** | Reader rating of perceived visual or image quality. | Likert or numeric scale | Usually, higher values indicate better quality, but the original scale direction should be checked. |
|  | **Artefact Likert score** | Reader rating of artefacts or technical image flaws. | Likert or numeric scale | Direction depends on whether the scale measures artefact presence or artefact absence. |
| **Clinical plausibility or usefulness rating** | **Clinical plausibility Likert score** | Reader rating of clinical plausibility, acceptability, or realism. | Likert or numeric scale | Usually, higher values indicate greater clinical plausibility. |
|  | **Pathology realism Likert score** | Reader rating of how realistic pathological findings appear. | Likert or numeric scale | Usually, higher values indicate greater pathological realism. |
|  | **Clinical usefulness score** | Reader rating of perceived clinical usefulness or applicability. | Likert or numeric scale | Usually, higher values indicate greater perceived usefulness. |
|  | **Clinical plausibility rate** | Proportion of images judged clinically plausible, acceptable, or usable. | % or proportion | Higher values indicate greater clinical plausibility or acceptability. |
| **Preference or ranking task** | **Preference rate** | Proportion of cases in which readers preferred one image type, model, or condition. | % or proportion | Direction depends on which option was coded as preferred. |
|  | **Ranking score** | Reader ranking score for image quality, realism, preference, or suitability. | Rank or score | Direction depends on the ranking convention used in the original study. |
| **Diagnostic interpretation task** | **Reader diagnostic accuracy** | Diagnostic accuracy achieved by readers when interpreting real and/or synthetic images. | % or proportion | Higher values indicate better diagnostic performance. |
|  | **Reader diagnostic sensitivity** | Reader sensitivity for detecting the target condition or class. | % or proportion | Higher values indicate better sensitivity. |
|  | **Reader diagnostic specificity** | Reader specificity for detecting or excluding the target condition or class. | % or proportion | Higher values indicate better specificity. |
|  | **Reader diagnostic AUC** | Reader-level or reader-task AUC for diagnostic interpretation. | AUC | Higher values indicate better diagnostic discrimination. |
| **Feature identification or annotation** | **Feature identification accuracy** | Accuracy with which readers identify, annotate, or recognize relevant anatomical, pathological, or radiological features. | % or proportion | Higher values indicate better feature recognition. |
| **Text-image matching** | **Text-image matching accuracy** | Accuracy with which readers match images to prompts, captions, reports, or clinical descriptions. | % or proportion | Higher values indicate better text-image correspondence. |
| **Interrater agreement** | **Interrater agreement** | Agreement between readers, including kappa, ICC, percentage agreement, or related statistics. | Kappa, ICC, %, or other | Interpretation depends on the agreement statistic used. |
| **Other structured reader endpoint** | **Other reader metric** | Structured reader endpoint that could not be mapped to a predefined metric subtype. | Varies | Interpretation depends on the original endpoint. |
|  | **Unspecified reader metric** | Reader endpoint reported without enough detail to map to a specific metric subtype. | Varies | Interpretation is unclear or requires manual verification. |

## Table S4. Interpretation and minimum reporting guidance for canonical human-reader endpoint/task categories.

Our selection is informed by generic methodological work on evaluation metrics for generative models [1–5] and by domain-specific work in medical imaging synthetic data [6–9].

| Canonical endpoint/task category | Relevant canonical metrics | Interpretation | Main caveat | Minimum recommended reporting |
| --- | --- | --- | --- | --- |
| **Real-vs-synthetic discrimination / Turing-style task** | **Turing accuracy; Turing sensitivity; Turing specificity; Turing AUC; Synthetic authenticity rate; Real authenticity rate; Fooling rate** | Values near chance can indicate that synthetic images are difficult to distinguish from real images; direction depends on the positive class. | Class balance, positive-class definition, reader aggregation, and uncertainty materially affect interpretation. | Report the confusion matrix or class-specific rates, reader numbers, image numbers, and the positive-class definition. |
| **Realism or image-quality rating** | **Realism Likert score; Quality Likert score; Artefact Likert score** | Higher scores usually indicate better perceived realism or quality, unless the scale measures artefacts or errors. | Scales, anchors, and aggregation differ across studies and should not be pooled without harmonization. | Report the full scale, anchors, direction, reader group, and separate real/synthetic results. |
| **Clinical plausibility or usefulness rating** | **Clinical plausibility Likert score; Pathology realism Likert score; Clinical usefulness score; Clinical plausibility rate** | Higher values usually indicate greater clinical plausibility, acceptability, or usefulness. | Plausibility is context- and specialty-dependent and is not equivalent to diagnostic utility. | Report the clinical question, reader expertise, scale anchors, and acceptance threshold. |
| **Preference or ranking task** | **Preference rate; Ranking score** | Results describe relative preference or rank rather than absolute image quality. | Direction depends on the comparator and ranking convention; ties and repeated ratings can change estimates. | Report comparators, randomization, tie handling, and the ranking or preference rule. |
| **Diagnostic interpretation task** | **Reader diagnostic accuracy; Reader diagnostic sensitivity; Reader diagnostic specificity; Reader diagnostic AUC** | Higher diagnostic performance generally indicates better reader performance for the specified clinical task. | Performance depends on prevalence, case mix, thresholds, and whether real and synthetic images were analysed separately. | Report prevalence, reader-level and pooled estimates, uncertainty, thresholds, and image group. |
| **Feature identification or annotation** | **Feature identification accuracy** | Higher accuracy generally indicates better identification, localisation, or annotation of relevant features. | Metrics may combine different anatomical structures, tasks, or tolerances. | Report the target feature, tolerance, reference standard, and reader-level results. |
| **Text-image matching** | **Text-image matching accuracy** | Higher accuracy indicates stronger correspondence between images and the associated text or prompt. | Chance level and task difficulty depend on the number and similarity of response options. | Report the candidate set, matching procedure, chance level, and reader expertise. |
| **Qualitative expert review** | **Narrative / qualitative judgement** | Narrative expert review provides contextual evidence but does not yield a directly comparable pooled numeric metric. | Selective examples, unclear procedures, and absent blinding can introduce substantial bias. | Report reviewer expertise, sampling, blinding, review criteria, and representative findings. |
| **Interrater agreement** | **Interrater agreement** | Higher agreement generally indicates greater consistency between readers. | Kappa, ICC, and percentage agreement are not interchangeable and are sensitive to prevalence and scale. | Report the statistic, weighting, confidence interval, number of readers, and unit of analysis. |
| **Other structured reader endpoint** | **Other reader metric; Unspecified reader metric** | Interpretation follows the original endpoint definition. | Non-standard endpoints may not be comparable across studies. | Preserve the original wording and report the scale, direction, and analysis rule. |

## Table S5. Automated image-quality metric families.

| Automated metric family | Definition / coding rule | Examples of included metrics |
| --- | --- | --- |
| **Distributional similarity or fidelity** | Metrics comparing the statistical distribution of real and synthetic images, either directly or in a learned feature-embedding space. Metrics were coded in this family when their purpose was to quantify how closely the synthetic image distribution matched the real-image distribution. | FID; MMD; KID; KL divergence; Jensen–Shannon divergence; sliced Wasserstein distance; Wasserstein distance; SIFID; CMMD; 3D-MMD. |
| **Pixel/structural similarity** | Metrics assessing pixel-level, structural, or signal similarity between generated images and paired or reference images. Metrics were coded in this family when they quantified image similarity using spatial or structural correspondence. | SSIM; MS-SSIM; 3D-SSIM; PSNR. |
| **Diversity or coverage** | Metrics assessing whether the generated image set captured the variability, heterogeneity, or coverage of the real-data distribution. Metrics were coded in this family when they measured diversity, mode coverage, or representation of the real-image space. | Entropy; generative precision; generative recall; density and coverage; PRD-AUC; feature-relative diversity; pixel-relative diversity; texture-relative diversity. |
| **Automated realism/no-reference quality** | Metrics intended to assess the apparent realism, quality, or recognisability of synthetic images without requiring a paired reference image. This family included classifier-, discriminator-, or signal-based quality proxies when used as automated image-quality endpoints. | Inception Score; SNR; predicted class confidence; discriminator score; generator score; CNN conventional-vs-synthetic classification accuracy. |
| **Intensity, texture, and image-statistical descriptors** | Metrics comparing low-level image properties, including intensity, contrast, colour-channel statistics, texture, or morphology-related descriptors. Metrics were coded in this family when they described differences in basic image-statistical characteristics rather than global feature-distribution similarity. | Brightness drift; RMS contrast drift; mean red, green, or blue relative-intensity drift; pixel grayscale mean; pixel grayscale standard deviation; skewness; vessel amount difference. |
| **Reconstruction error** | Metrics quantifying absolute or squared reconstruction error between generated and reference images. Metrics were coded separately from structural similarity metrics because they directly measure numerical reconstruction differences. | MSE; MAE; RMSE. |
| **Perceptual similarity** | Metrics assessing perceptual similarity using learned deep-feature representations rather than direct pixel-wise comparison. | LPIPS. |
| **Privacy/memorisation similarity** | Metrics used to evaluate whether synthetic images were overly similar to training, reference, or real images, suggesting potential memorisation or privacy leakage. | Euclidean distance in InceptionV3 feature space; nearest-neighbour or feature-space similarity measures when used for privacy or memorisation assessment. |
| **Other automated image-quality metric** | Automated metrics that were author-defined, study-specific, insufficiently standardised, or could not be clearly mapped to the predefined families above. | GS; JPEG file size; t-SNE mean distance; chi-square distance; K-means correctly clustered; K-means discarded; MODE score; automated structure-detection success rate; minimum Euclidean distance. |

## Table S6. Canonical definitions of automated image-quality metrics reported in the review.

| Automated metric family | Canonical metric | Definition / coding rule | Typical unit or scale | Interpretation note |
| --- | --- | --- | --- | --- |
| **Automated realism/no-reference quality** | **IS** | Inception Score or related no-reference score combining recognizability and diversity of generated images. | Continuous score | Higher values usually indicate greater apparent realism and diversity. |
|  | **SNR** | Signal-to-noise ratio used as an automated quality or image-noise proxy. | Continuous score, often decibels | Higher values usually indicate better signal quality. |
|  | **Predicted class confidence** | Confidence assigned by a classifier to generated images. | % or probability | Higher values indicate that generated images are more confidently recognized by the classifier. |
|  | **SNR difference** | Difference in signal-to-noise ratio between real and synthetic images. | Continuous difference score | Lower values indicate greater similarity between real and synthetic image signal properties. |
|  | **Discriminator score** | Discriminator-based output or score used as an automated quality proxy. | Varies | Interpretation depends on the discriminator output and coding in the original study. |
|  | **Generator score** | Generator-based output or score used as an automated quality proxy. | Varies | Interpretation depends on the generator output and coding in the original study. |
|  | **CNN conventional-vs-synthetic classification accuracy** | Accuracy of a CNN in distinguishing conventional or real images from synthetic images. | % or proportion | Lower values may indicate greater realism if the task is balanced, and synthetic images are harder to distinguish from real images. |
| **Distributional similarity or fidelity** | **FID** | Fréchet Inception Distance, measuring the distance between real and synthetic image feature distributions. | Continuous distance score | Lower values indicate greater similarity between real and synthetic image distributions. |
|  | **KL divergence** | Kullback–Leibler divergence between real and synthetic distributions or extracted feature distributions. | Continuous divergence score | Lower values indicate greater distributional similarity; asymmetric by definition. |
|  | **MMD** | Maximum mean discrepancy between real and synthetic data or feature distributions. | Continuous distance score | Lower values indicate greater distributional similarity. |
|  | **KID** | Kernel Inception Distance, a kernel-based distance between real and synthetic image feature distributions. | Continuous distance score | Lower values indicate greater distributional similarity. |
|  | **SIFID** | Single-image Fréchet Inception Distance or related single-image feature-distribution distance. | Continuous distance score | Lower values indicate greater similarity at the single-image or local distribution level. |
|  | **CMMD** | Maximum mean discrepancy variant used to compare real and synthetic feature distributions. | Continuous distance score | Lower values indicate greater distributional similarity. |
|  | **3D-MMD** | Maximum mean discrepancy calculated for 3D images, volumes, or 3D feature representations. | Continuous distance score | Lower values indicate greater similarity, but values may not be comparable with standard 2D MMD because of different scaling. |
|  | **Jensen-Shannon divergence** | Symmetric divergence between real and synthetic data or feature distributions. | Continuous divergence score | Lower values indicate greater distributional similarity. |
|  | **KST** | Study-specific distributional test statistic comparing real and synthetic data. | Continuous statistic | Lower values were interpreted as greater similarity in the source study. |
|  | **SWD** | Sliced Wasserstein distance between real and synthetic image distributions. | Continuous distance score | Lower values indicate greater distributional similarity. |
|  | **Wasserstein distance** | Distance between real and synthetic samples or feature distributions based on optimal transport. | Continuous distance score | Lower values indicate greater distributional similarity. |
| **Diversity or coverage** | **Generative precision** | Metric estimating the fidelity of generated samples relative to the real-data distribution. | Proportion or score | Higher values usually indicate that generated samples lie closer to the real-data distribution. |
|  | **Generative recall** | Metric estimating how well generated samples cover the real-data distribution. | Proportion or score | Higher values usually indicate greater coverage of the real-data distribution. |
|  | **Entropy** | Measure of image information content, variability, or uncertainty. | Continuous score | Interpretation is target-dependent; higher values often indicate greater variability or information content. |
|  | **Density and coverage** | Metrics describing density of generated samples and coverage of the real feature distribution. | Proportion or score | Interpretation depends on the implementation; usually higher coverage indicates better representation of the real-data space. |
|  | **JPEG file size** | File-size-based proxy for image complexity or compressibility. | File size, usually KB or bytes | Interpretation is indirect and context-dependent. |
|  | **Recall for generative-model diversity proxy** | Study-specific recall-like metric used as a proxy for generative diversity or coverage. | % or proportion | Higher values generally indicate greater diversity or coverage, depending on the original definition. |
|  | **Standard deviation of extracted features** | Variability of extracted image features across generated images. | Continuous score | Interpretation is target-dependent and depends on the feature representation used. |
|  | **Feature-relative diversity** | Relative diversity measured in a feature-embedding space. | Continuous score | Interpretation depends on the original study definition. |
|  | **MODE score** | Author-defined or study-specific quality/diversity score. | Continuous score | Interpretation depends on the original study definition. |
|  | **Pixel-relative diversity** | Relative diversity measured at pixel level. | Continuous score | Interpretation depends on the original study definition. |
|  | **Texture-relative diversity** | Relative diversity measured using texture features. | Continuous score | Interpretation depends on the original study definition. |
|  | **PRD-AUC** | Area under the precision–recall distribution curve for generative-model evaluation. | AUC or continuous score | Higher values usually indicate a better balance between fidelity and diversity. |
| **Intensity, texture, and image-statistical descriptors** | **Brightness drift** | Difference in brightness between real and synthetic images. | Continuous difference score | Lower values indicate smaller brightness differences. |
|  | **Mean blue relative-intensity drift** | Difference in blue-channel intensity between real and synthetic images. | Continuous difference score | Lower values indicate smaller blue-channel intensity differences. |
|  | **Mean green relative-intensity drift** | Difference in green-channel intensity between real and synthetic images. | Continuous difference score | Lower values indicate smaller green-channel intensity differences. |
|  | **Mean red relative-intensity drift** | Difference in red-channel intensity between real and synthetic images. | Continuous difference score | Lower values indicate smaller red-channel intensity differences. |
|  | **RMS contrast drift** | Difference in root-mean-square contrast between real and synthetic images. | Continuous difference score | Lower values indicate smaller contrast differences. |
|  | **Pixel grayscale mean** | Average grayscale intensity of images. | Intensity value | Interpretation is target-dependent and depends on the image scale. |
|  | **Pixel grayscale standard deviation** | Variability in grayscale intensity. | Intensity value or continuous score | Interpretation is target-dependent and depends on the image scale. |
|  | **Skewness** | Asymmetry of the pixel-intensity distribution. | Continuous score | Interpretation is target-dependent. |
|  | **Vessel amount difference** | Difference in vessel amount between real and synthetic images. | Continuous difference score | Lower values indicate smaller differences in vessel-related image content. |
| **Other automated image-quality metric** | **GS** | Author-defined automated image-quality score. | Varies | Interpretation depends on the original study definition. |
|  | **Precision for generative-model quality proxy** | Study-specific precision-like proxy for generated-image quality or fidelity. | % or proportion | Higher values generally indicate better quality or fidelity, depending on the original definition. |
|  | **Chi-square distance** | Distance or discrepancy between distributions. | Continuous distance score | Usually lower values indicate greater similarity, but interpretation depends on the original implementation. |
|  | **Contour F1 score** | F1 score calculated for detected or reconstructed contours. | Score or proportion | Higher values indicate better contour agreement. |
|  | **Contour IoU** | Intersection over union calculated for contours. | Score or proportion | Higher values indicate greater contour overlap. |
|  | **Contour PPV** | Positive predictive value calculated for contour detection. | % or proportion | Higher values indicate greater contour precision. |
|  | **Contour TPR** | True positive rate calculated for contour detection. | % or proportion | Higher values indicate greater contour sensitivity. |
|  | **K-means correctly clustered** | Percentage or count of images correctly clustered using k-means. | % or count | Higher values indicate better clustering according to the study-defined criterion. |
|  | **K-means discarded** | Percentage or count of images discarded after k-means quality control. | % or count | Lower values usually indicate fewer images rejected by the quality-control procedure. |
|  | **Minimum Euclidean distance** | Minimum distance between samples or features. | Continuous distance score | Interpretation depends on whether distance was used as a similarity, diversity, or privacy-related proxy. |
|  | **SD** | Study-reported standard-deviation descriptor; the original article should be consulted for the exact target. | Continuous value | Interpretation is target-dependent. |
|  | **t-SNE mean distance** | Mean distance between samples in a t-SNE embedding space. | Continuous distance score | Interpretation depends on the embedding and study-specific definition. |
|  | **Automated structure-detection success rate** | Success rate of an automated structure-detection procedure applied to generated images. | % or proportion | Higher values indicate that relevant structures were more often detected successfully. |
| **Perceptual similarity** | **LPIPS** | Learned Perceptual Image Patch Similarity, measuring perceptual feature distance between images. | Continuous distance score | Lower values indicate greater perceptual similarity. |
| **Pixel/structural similarity** | **SSIM** | Structural Similarity Index Measure comparing generated and reference images. | Score, commonly 0–1 | Higher values indicate greater structural similarity. |
|  | **MS-SSIM** | Multiscale Structural Similarity Index Measure comparing images across multiple spatial scales. | Score, commonly 0–1 | Higher values indicate greater multiscale structural similarity. |
|  | **3D-SSIM** | Structural similarity calculated for 3D images or volumes. | Score, commonly 0–1 | Higher values indicate greater 3D structural similarity. |
| **Privacy/memorisation similarity** | **Euclidean distance in InceptionV3 feature space** | Feature-space distance between generated and reference images, used to assess similarity or possible memorization. | Continuous distance score | Interpretation is context-dependent; unusually small distances may suggest high similarity to reference or training images. |
| **Reconstruction error** | **PSNR** | Peak signal-to-noise ratio between generated and reference images. | Decibels or continuous score | Higher values indicate lower reconstruction distortion. |
|  | **MSE** | Mean squared error between generated and reference images. | Continuous error score | Lower values indicate smaller reconstruction error. |
|  | **MAE** | Mean absolute error between generated and reference images. | Continuous error score | Lower values indicate smaller reconstruction error. |
|  | **RMSE** | Root mean squared error between generated and reference images. | Continuous error score | Lower values indicate smaller reconstruction error. |

## Table S7. Risk of bias criteria and assessment levels.

Each study was evaluated based on predefined methodological criteria, including sampling strategy, population description, architecture description, outcome reporting, data completeness, synthetic image generation and validation methods, and poor-quality image information. Risk of bias is categorized into three levels: low-risk for full compliance, medium-risk for partial compliance or lack of clarity, and high-risk for missing or insufficient information.

| Criterion | Description | Low risk | Medium-risk | High-risk |
| --- | --- | --- | --- | --- |
| **Sampling strategy** | The sampling strategy is adequately described for assessing external validity. | A source explaining the data collection process is provided, or the process is described directly. | The sampling strategy is only partially explained. | The sampling strategy is not described at all. |
| **Population description** | The original image source population is well described in terms of severity, clinical characteristics, stage (if applicable), etc. | The population is fully described, or a reference is provided where it is well described. | A source is mentioned, but its usage is unclear. | The population is not described, nor is a reference provided. |
| **Quality control** | Information is provided about missing images or images excluded due to poor quality or inappropriateness. | Preprocessing and data exclusion criteria are clearly described. If using a well-documented dataset, it is considered green by default. | Mentions preprocessing but does not explain how it was done. | Mentions poor quality images but provides no information about them. |
| **Architecture description** | The method for generating synthetic images is clearly described or referenced. | The architecture and exact parameters are explained in detail. | The architecture is mentioned but not detailed. | No explanation of the architecture is provided. |
| **Image generation *vs.* validation** | Different image sets are used for image generation and validation. | Validation and generation set sizes (N) are reported, and they are different. | One of the two conditions is not met (either N is not reported, or the sets are not fully separate). | Neither condition is met (N is not reported, and there is overlap between sets). |
| **Outcome definition and reporting** | The outcomes used for assessing performance/validity are fully described. | All outcomes and formulas used are explained, or a reference is provided where they are described. | Some outcomes are explained, but details are missing (e.g., mentioning a neural network improving results by 5% without explaining how the NN works). | No outcomes or formulas are described. |
| **Data completeness** | The study reports summary statistics such as the mean and at least one measure of variance (e.g., confidence intervals, standard deviation, sample size). | Both mean and complete variance measures are reported. | Mean is provided, but variance measures are partially provided (e.g., standard deviation without sample size, variance is not provided for all metrics). | Only the mean is reported or there is a lot of missing information. |

# Supplementary results

Supplementary results are presented as they appear in the main text, irrespective of whether they are represented in a Table or a Figure.

## Table S8. Overview of the 160 included studies on synthetic medical imaging.

| Study | Imaging technique | Medical condition / setting | Generation model(s) | Automated image-quality metrics | Human-reader task(s) | Downstream performance | XAI / explainability |
| --- | --- | --- | --- | --- | --- | --- | --- |
| Bargsten and Schlaefer, 2020 [10] | IVUS | Cardiovascular disease [Cardiology / cardiovascular disease] | Conditional GAN with theory-guided adaptive speckle layer; Conditional GAN without speckle layer; SpeckleGAN | FID; Jensen-Shannon divergence; SSIM | Not performed | Segmentation: Dice; Hausdorff distance | Not performed |
| Cha et al., 2020 [11] | X-ray | Breast mass detection [Oncology] | Procedural analytic breast modeling and physics-based x-ray simulation; procedural analytic modeling + Monte Carlo-Based X-ray Simulation | Not performed | Not performed | Classification; Detection / localisation: Recall / sensitivity | Latent-space visualization or interpretation |
| Chedid et al., 2020 [12] | X-ray | Radiology [Orthopedics / musculoskeletal / rheumatology] | Conditional GAN / pix2pixHD image-to-image translation; pix2pixHD-based image synthesis pipeline with Gaussian process optimization; pix2pixHD-based image synthesis pipeline with optimization-based human-guided refinement; pix2pixHDGAN | Not performed | Real-vs-synthetic discrimination / Turing-style task; Preference or ranking task | Classification: Accuracy; Recall / sensitivity | Grad-CAM/CAM family |
| Cronin et al., 2020 [13] | Ultrasound | Sport and exercise science; Neuromuscular research [Orthopedics / musculoskeletal / rheumatology] | Cycle-consistent GAN / Unsupervised Data to Content Transformation (UDCT); UDCT (cycleGAN) | Entropy; Skewness | Not performed | Segmentation; Detection / localisation; Regression/measurement: IoU / Jaccard; Pennation angle | Not performed |
| Diller et al., 2020 [14] | MRI | Congenital heart disease [Cardiology / cardiovascular disease] | PG-GAN; PGGAN; Progressive generative adversarial network (PG-GAN); Progressively growing generative adversarial network | Not performed | Real-vs-synthetic discrimination / Turing-style task; Clinical plausibility or usefulness rating | Segmentation: Dice | Not performed |
| He et al., 2020 [15] | OCT | Macular retinal conditions [Ophthalmology] | Label smoothing generative adversarial network (LSGAN; DCGAN architecture with WGAN-GP loss); GAN; Label smoothing generative adversarial network (LSGAN) | Not performed | Not performed | Classification: F1-score; Precision / PPV; Recall / sensitivity; Specificity | Example-based prediction explanation; Qualitative error analysis |
| Islam and Zhang, 2020 [16] | PET | Alzheimer (3 stages) [Neurology] | DCGAN; GAN / DCGAN-like convolutional GAN | PSNR; SSIM | Not performed | Classification: Accuracy | Not performed |
| Miyoshi et al., 2020 [17] | Coronary angioscopy | Coronay artery disease [Cardiology / cardiovascular disease] | Conditional GAN; cGAN; GAN | IS | Diagnostic interpretation task; Feature identification or annotation | Classification; Regression/measurement: AUC; F1-score; MAE; Precision / PPV; Recall / sensitivity | Not performed |
| Nishio et al., 2020 [18] | CT | Lung cancer [Oncology] | GAN model without nodule size information; Proposed GAN with nodule size information; Proposed GAN with nodule size information; comparator GAN without nodule size information; Conditional GAN / pix2pix; Proposed 3D GAN / 3D pix2pix-like model with nodule size information; pix2pix | Not performed | Real-vs-synthetic discrimination / Turing-style task; Realism or image-quality rating | Classification: Accuracy | Not performed |
| Qin et al., 2020 [19] | Dermoscopy | Dermathology [Dermatology] | DCGAN; GAN; Modified StyleGAN; SL-StyleGAN; SL-StyleGAN / skin lesion style-based GAN; StyleGAN | FID; Generative precision; Generative recall; IS | Qualitative expert review | Classification: Accuracy; Precision / PPV; Recall / sensitivity; Specificity | Latent-space visualization or interpretation |
| Shi et al., 2020 [20] | Ultrasound | Thyroid nodule [Oncology] | Auxiliary Classifier GAN; KACGAN; KACGAN / knowledge-guided ACGAN | IS | Real-vs-synthetic discrimination / Turing-style task; Diagnostic interpretation task | Classification: Accuracy; AUC; Recall / sensitivity; Specificity | Not performed |
| Waheed et al., 2020 [21] | X-ray | Covid-19 [Pulmonology / respiratory disease] | ACGAN; Auxiliary Classifier GAN | Not performed | Not performed | Classification: Accuracy; F1-score; Precision / PPV; Recall / sensitivity; Specificity | Latent-space visualization or interpretation |
| Wang et al., 2020 [22] | CT | Subcentimeter Pulmonary Adenocarcinoma [Pulmonology / respiratory disease] | Progressive growing Wasserstein GAN; Progressive-growing wGAN; WGAN-GP + CNN; WGAN-GP and conditional GAN | Not performed | Real-vs-synthetic discrimination / Turing-style task | Classification: Accuracy | Latent-space visualization or interpretation |
| Yoo et al., 2020 [23] | Clinical photography | Severe pharyngitis (Tonsillitis) [Otorhinolaryngology / ENT] | CycleGAN | Not performed | Qualitative expert review | Classification; Detection / localisation: Accuracy; AUC; Recall / sensitivity; Specificity | Grad-CAM/CAM family; Saliency map or heatmap; Latent-space visualization or interpretation |
| Yu and Zhang, 2020 [24] | MRI | Prostate Cancer [Oncology] | CapGAN; comparators DCGAN and LSGAN; DCGAN and least-squares GAN; DCGAN-derived GAN with capsule discriminator and least-squares loss; CapGAN | KL divergence | Qualitative expert review | Classification: Accuracy; AUC | Latent-space visualization or interpretation |
| Zheng et al., 2020 [25] | OCT | Retinal diseases (Choroidal neovascularization, diabetic macular edema, drusen) [Ophthalmology] | PGGAN; Progressively grown GANs with sketch guidance modules; Progressively grown generative adversarial network with sketch guidance module | FID | Real-vs-synthetic discrimination / Turing-style task; Realism or image-quality rating; Diagnostic interpretation task | Classification: Accuracy; AUC; Recall / sensitivity; Specificity | Not performed |
| de Souza et al., 2020 [26] | Endoscopy | Barrett's esofhagus and adenocarcinoma [Oncology] | DCGAN | FID; MSE; SSIM | Not performed | Classification: Accuracy | Not performed |
| Acar et al., 2021 [27] | CT | COVID-19 [Pulmonology / respiratory disease] | GAN | Not performed | Not performed | Classification: Accuracy; F1-score; Precision / PPV; Recall / sensitivity; Specificity | Not performed |
| Ahmad et al., 2021 [28] | Dermoscopy | Skin cancer [Dermatology] | TED-GAN; VAE-GAN with auxiliary classifier and Student t-distribution noise | Not performed | Not performed | Classification: Accuracy; Recall / sensitivity | Other/unclear |
| Durán-Rosal et al., 2021 [29] | DaTscan | Parkinson’s disease [Neurology] | Statistical Distribution Modeling of Voxels; Statistical data augmentation / distribution-fitting synthetic pattern generation | Not performed | Not performed | Regression/measurement | SHAP/LIME/feature attribution |
| Gao et al., 2021 [30] | MRI | Sarcoma [Oncology] | ACGAN; Auxiliary Classifier GAN; Auxiliary classifier generative adversarial network (ACGAN) | Not performed | Qualitative expert review | Classification: Accuracy; Recall / sensitivity; Specificity | Other/unclear |
| Gómez et al., 2021 [31] | MRI | Cardiovascular diseases [Cardiology / cardiovascular disease] | PGGAN; Progressive GAN | MS-SSIM; SWD | Not performed | Classification: Accuracy | Latent-space visualization or interpretation |
| He et al., 2021 [32] | Endoscopy | Polyp detection [Gastroenterology / hepatology] | GAN / SinGAN plus adversarial attack; SinGAN; SinGAN + DAG () | Not performed | Not performed | Classification: Recall / sensitivity; Specificity | Not performed |
| Jiang et al., 2021 [33] | CT | COVID-19 diagnosis [Pulmonology / respiratory disease] | Conditional GAN / semantic-layout-conditional image-to-image translation; cGAN | FID; PSNR; RMSE; SSIM | Not performed | Segmentation; Classification: Dice; Recall / sensitivity; Specificity | Not performed |
| Karakanis and Leontidis, 2021 [34] | X-ray | Lung cancer [Oncology] | Conditional GAN; cGAN | Not performed | Not performed | Classification: Accuracy; Recall / sensitivity; Specificity | Grad-CAM/CAM family; Occlusion or perturbation analysis |
| Karbhari et al., 2021 [35] | X-ray | COVID-19 diagnosis [Pulmonology / respiratory disease] | ACGAN; Auxiliary Classifier GAN | FID; IS | Not performed | Classification: Accuracy; AUC; F1-score; Precision / PPV; Recall / sensitivity | Not performed |
| Kim et al., 2021 [36] | X-ray | Medical Imaging [Dental medicine] | PGGAN; Progressive growing generative adversarial network (PGGAN) | SNR | Real-vs-synthetic discrimination / Turing-style task; Feature identification or annotation | Classification: Accuracy | Not performed |
| Kossen et al., 2021 [37] | MRI | Angiography [Cardiology / cardiovascular disease] | DCGAN; Deep convolutional GAN; WGAN-GP; WGAN-GP-SN; Wasserstein GAN with gradient penalty; Wasserstein GAN with gradient penalty and spectral normalization | FID | Not performed | Segmentation: Dice | Not performed |
| Liu et al., 2021 [38] | CT | Liver tumor [Oncology] | MAGAN (Mask Attention Generative Adversarial Network) | PSNR | Real-vs-synthetic discrimination / Turing-style task | Segmentation: Dice | Not performed |
| Montero et al., 2021 [39] | Ultrasound | Fetal brain ultrasound plane classification [Obstetrics / pregnancy] | GAN; StyleGAN2-ADA | FID; Generative precision; Generative recall | Not performed | Classification: Accuracy; AUC; F1-score | Not performed |
| Morís et al., 2021 [40] | X-ray | COVID-19 diagnosis [Pulmonology / respiratory disease] | CycleGAN; CycleGAN - ResNet-9 | Not performed | Not performed | Classification: Accuracy; F1-score | Not performed |
| Mutepfe et al., 2021 [41] | Dermoscopy | Skin lesion cancer (2 stages) [Oncology] | DCGAN | FID | Not performed | Classification: Accuracy; AUC; F1-score; Precision / PPV; Recall / sensitivity | Not performed |
| Park et al., 2021b [42] | MRI | Isocitrate dehydrogenase (IDH)-mutant glioblastomas [Oncology] | GAN; StyleGAN2; StyleGAN2 synthetic data used in model 2 and model 3 training | Not performed | Real-vs-synthetic discrimination / Turing-style task; Diagnostic interpretation task; Feature identification or annotation | Classification; Regression/measurement: AUC; Recall / sensitivity; Specificity | SHAP/LIME/feature attribution; Latent-space visualization or interpretation |
| Rangarajan and Ramachandran, 2021 [43] | X-ray | Covid-19 [Pulmonology / respiratory disease] | DCGAN; GAN | Not performed | Not performed | Classification: Accuracy | Not performed |
| Sajjad et al., 2021 [44] | PET | Alzheimer's disease [Neurology] | DCGAN | PSNR; SSIM | Not performed | Classification: Accuracy | Not performed |
| Segal et al., 2021 [45] | X-ray | Chest diseases [Pulmonology / respiratory disease] | PGGAN; Progressively Growing GAN (PGGAN) | FID | Real-vs-synthetic discrimination / Turing-style task | Classification: AUC | Latent-space visualization or interpretation |
| Shen et al., 2021 [46] | X-ray | Breast mass [Oncology] | Conditional GAN / image infilling GAN; DCGAN; DCGAN + Infilling GAN | LPIPS | Not performed | Classification: Recall / sensitivity | Not performed |
| Toda et al., 2021 [47] | CT | Lung cancer [Oncology] | InfoGAN / semi-conditional GAN; WGAN | FID | Not performed | Classification: Accuracy; Specificity | Latent-space visualization or interpretation |
| V J and D, 2021 [48] | X-ray | COVID-19 diagnosis [Pulmonology / respiratory disease] | Deep convolutional GAN | Not performed | Not performed | Classification: Accuracy; AUC | Not performed |
| Wang et al., 2021 [49] | X-ray | lung cancer [Oncology] | Conditional GAN; pix2pix/U-Net-based MGGAN plus WGSLN; MTCGAM: pix2pix + Multi-Target Co-Guided Adversarial Mechanism | FID | Not performed | Classification: Accuracy; F1-score; Recall / sensitivity | Not performed |
| Zheng et al., 2021 [50] | OCT | Closed-Angle detection [Ophthalmology] | PGGAN; Progressively grown generative adversarial network; Progressively grown generative adversarial networks | Not performed | Realism or image-quality rating; Feature identification or annotation | Classification: AUC | Latent-space visualization or interpretation |
| Ahmad et al., 2022 [28] | MRI | Brain tumor [Oncology] | ED-GAN (VAE-GAN); VAE plus GAN | IS | Not performed | Classification: Accuracy; F1-score; Precision / PPV; Recall / sensitivity; Specificity | Not performed |
| Albahli and Yar, 2022 [51] | X-ray | Covid-19 and other chest diseases (14) [Pulmonology / respiratory disease] | DCGAN; Deep convolutional GAN | Not performed | Not performed | Classification: Accuracy | Not performed |
| Alrashedy et al., 2022 [52] | MRI | Brain tumor [Oncology] | DCGAN; Deep convolutional GAN; GAN; Vanilla GAN | Not performed | Not performed | Classification: Accuracy; AUC; Precision / PPV; Recall / sensitivity; Loss | Not performed |
| Amin et al., 2022 [53] | CT | Liver tumor [Oncology] | GAN / DCGAN-like adversarial neural network; GAN | Discriminator score; Generator score | Not performed | Classification; Segmentation: Accuracy; F1-score; Precision / PPV; Recall / sensitivity; Specificity; IoU / Jaccard | Not performed |
| Chai et al., 2022 [54] | Multiple imaging techniques | Medical imaging [Radiology / medical imaging] | DPGAN; Hierarchical GAN composed of three VAEGAN stages with an extra image-code discriminator | FID; IS | Not performed | Segmentation: Dice; IoU / Jaccard | Not performed |
| Coyner et al., 2022 [55] | Fundus photography | Macular degeneration and diabetic macular edema [Ophthalmology] | PGGAN; Progressively growing generative adversarial network (PGAN) | Euclidean distance in inceptionv3 feature space; FID | Not performed | Classification: Accuracy; AUC; Precision / PPV; Recall / sensitivity; Specificity; NPV | Latent-space visualization or interpretation |
| Gan and Wang, 2022 [56] | OCT | Esophageal lesions [Oncology] | Hybrid VAE/GAN-style adversarially trained variational autoencoder without extra discriminator; AL-VAE | MMD; MODE score; Wasserstein distance | Not performed | Segmentation: Dice | Latent-space visualization or interpretation |
| Gheorghiță et al., 2022 [57] | MRI | Cardiovascular disease [Cardiology / cardiovascular disease] | GAN / semantic image synthesis / GauGAN with SPADE; GauGAN | Not performed | Not performed | Classification; Regression/measurement: RMSE; Correlation | Not performed |
| Guan et al., 2022 [58] | X-ray | Pancreatic tumor [Oncology] | TMP-GAN; TMP-GAN ablation - without reconstruction loss; TMP-GAN ablation - without texture continuity adversarial loss; TMP-GAN ablation - without texture representation adversarial loss; Texture-constrained multichannel progressive GAN | Not performed | Not performed | Classification; Detection / localisation: AUC; F1-score; Precision / PPV; Recall / sensitivity; Loss | Not performed |
| Guo et al., 2022 [59] | X-ray | Pediatric pneuomina [Pulmonology / respiratory disease] | GAN; RS-FFGAN | FID; IS | Not performed | Classification: Accuracy; F1-score; Precision / PPV; Recall / sensitivity | Training-dynamics visualization; Qualitative realism and artefact comparison; Distributional image-realism validation |
| Heenaye-Mamode Khan et al., 2022 [60] | Clinical photography | 15 categories of skin pathologies [Dermatology] | DCGAN; Deep convolutional GAN | Not performed | Not performed | Classification: Accuracy | Not performed |
| Hussain et al., 2022 [61] | X-ray | Respiratory diseases [Pulmonology / respiratory disease] | Wasserstein GAN; Wasserstein GAN (WGAN) | Not performed | Not performed | Classification: Accuracy; AUC; Precision / PPV; Specificity | Not performed |
| Kim et al., 2022 [62] | Fundus photography | Epiretinal Membrane (ERM) [Ophthalmology] | StyleGAN; StyleGAN with transfer learning for ERM image synthesis | SNR; Snr difference; Vessel amount difference | Real-vs-synthetic discrimination / Turing-style task; Feature identification or annotation; Qualitative expert review | Classification: Accuracy; AUC; Recall / sensitivity; Specificity | Grad-CAM/CAM family |
| Liang et al., 2022 [63] | Ultrasound | Various (Covid, hip joint, ovary and follicle) [Multiple clinical settings] | Mixed: spGAN-sketch; spGAN; spGAN v2; spGAN; spGAN v2; spGAN-sketch; spGAN-sketch; spGAN; spGAN v2; Sketch-guided progressive growing conditional GAN | FID; KID; LPIPS; MS-SSIM | Real-vs-synthetic discrimination / Turing-style task | Segmentation: Dice | Not performed |
| Mukherkjee et al., 2022 [64] | MRI | Brain tumor [Oncology] | AGGrGAN; Aggregated GAN with style transfer | KL divergence; PSNR; SD; SSIM | Not performed | Classification: Accuracy | Not performed |
| Ovalle-Magallanes et al., 2022 [65] | X-ray | Coronary artery disease / coronary stenosis [Other] | Hierarchical Bezier-based Generative Model (HBGM); Procedural generative model | FID | Not performed | Classification: Accuracy; F1-score; Precision / PPV; Recall / sensitivity; Specificity | Grad-CAM/CAM family |
| Qiang et al., 2022 [66] | MRI | Neurology | Recurrent WGAN | FID; Minimum euclidean distance | Not performed | Classification; Segmentation; Regression/measurement: Accuracy; AUC; Recall / sensitivity; Specificity; Dice; IoU / Jaccard; Hausdorff distance; Normalized surface Dice; Correlation | Latent-space visualization or interpretation; Quantitative validation of explanation maps; Other/unclear |
| Qin et al., 2022 [67] | X-ray | Pneumonia [Pulmonology / respiratory disease] | DCGAN; PGGAN | FID; MS-SSIM | Not performed | Classification: Accuracy; AUC; AUPRC; F1-score; G-mean; Precision / PPV; Recall / sensitivity; Specificity | Not performed |
| Ramirez et al., 2022 [68] | MRI | Pelvic organ prolapse [Other] | Deformation Field GAN (Vanilla GAN); GAN-based deformation generator | Not performed | Not performed | Segmentation: IoU / Jaccard | Not performed |
| Shah et al., 2022 [69] | X-ray | Breast mass [Oncology] | DCGAN | K-means correctly clustered; K-means discarded | Not performed | Classification: AUC | Grad-CAM/CAM family; Saliency map or heatmap |
| Sreejith Kumar et al., 2022 [70] | OCT | Glaucoma (3 stages) / healthy [Ophthalmology] | PGGAN; PGGAN for synthetic images; PGGAN glaucoma model; PGGAN glaucoma model for synthetic images; PGGAN normal model; PGGAN normal model for synthetic images; Progressively growing generative adversarial network; separate models for normal and glaucoma OCT images | FID | Real-vs-synthetic discrimination / Turing-style task; Realism or image-quality rating | Classification; Detection / localisation: AUC; Recall / sensitivity; Specificity | Grad-CAM/CAM family |
| Sun et al., 2022 [71] | Multiple imaging techniques | Chronic Obstructive Pulmonary Disease (COPD) [Pulmonology / respiratory disease] | 3D GAN; HA-GAN | FID; IS; MMD | Not performed | Classification: Accuracy | Latent-space visualization or interpretation; Counterfactual or example-based explanation |
| Thambawita et al., 2022 [72] | Endoscopy | Polyps [Gastroenterology / hepatology] | SinGAN+Style Transfer; Single-image GAN segmentation-data generator | FID; SIFID | Not performed | Classification; Segmentation: Accuracy; Precision / PPV; Recall / sensitivity; Dice; IoU / Jaccard; Loss | Not performed |
| Veturi et al., 2022 [73] | Fundus photography | Retinal diseases [Ophthalmology] | StyleGAN; StyleGAN2; StyleGAN2-ADA / SynthEye | Not performed | Real-vs-synthetic discrimination / Turing-style task | Classification: Kappa | Latent-space visualization or interpretation |
| Yoon et al., 2022 [74] | Endoscopy | Colorectal cancer [Oncology] | DCGAN and CycleGAN; StyleGAN2 | FID | Real-vs-synthetic discrimination / Turing-style task; Realism or image-quality rating; Clinical plausibility or usefulness rating; Feature identification or annotation | Classification: Accuracy; F1-score; Precision / PPV; Recall / sensitivity; Specificity; NPV | Latent-space visualization or interpretation |
| Zhao et al., 2022 [75] | OCT | Retinal diseases (Macular degeneration, Diabetic macular edema) [Ophthalmology] | Unconditional GAN; GAN + additional classifier | FID; PSNR; SSIM | Not performed | Classification: Accuracy; Recall / sensitivity; Specificity | Not performed |
| Bajhaiya et al., 2023 [76] | Endoscopy | Gastrointestinal uclers [Gastroenterology / hepatology] | VAE-GAN; VAE-GAN (VAE-GAN hybrids); Variational autoencoder plus GAN | IS; Precision for generative-model quality proxy; Recall for generative-model diversity proxy | Real-vs-synthetic discrimination / Turing-style task | Classification: Accuracy | Latent-space visualization or interpretation |
| Behara et al., 2023 [77] | Dermoscopy | Skin cancer [Oncology] | DCGAN; Deep convolutional GAN | Not performed | Not performed | Classification: Accuracy; F1-score; Precision / PPV; Recall / sensitivity; Specificity | Not performed |
| Buoso et al., 2023 [78] | MRI | Cardiomyopathy (3 stages) [Cardiology / cardiovascular disease] | CMRGenNet (StyleGAN2-ADA); StyleGAN2 with Adaptive Discriminator Augmentation plus DatasetGAN-style semantic label branch; Hybrid numerical phantom / biophysical simulation / signal-model-based CMR synthesis; VAE / statistical shape model; UNet image-to-image network / tissue-property texturizer; MRXCAT 2.0 | Not performed | Not performed | Segmentation; Regression/measurement: Dice; Strain; Displacement error | Latent-space visualization or interpretation |
| Dash et al., 2023 [79] | X-ray | COVID-19 detection [Infectious diseases] | CoVaD-GAN (Vanilla GAN + DCGAN); Combined Vanilla GAN and DCGAN data augmentation approach | PSNR; SSIM | Not performed | Classification; Detection / localisation: Accuracy; F1-score; Precision / PPV; Recall / sensitivity; Specificity | Not performed |
| Guo et al., 2023 [80] | Clinical photography | Skin diseases [Dermatology] | Adaptive GAN with Wasserstein-loss convergence metric; MedGAN (WGAN) | Not performed | Not performed | Classification: Accuracy | Not performed |
| Hardy et al., 2023 [81] | Ultrasound | Liver [Gastroenterology / hepatology] | Latent diffusion model; Latent diffusion models: semantic synthesis LDM and class-to-image LDM; Semantic synthesis LDM and class-to-image LDM pooled; Semantic-map–conditioned LDM (concatenation conditioning)(Diffusion model.Latent Diffusion Model (conditional)) | FID; IS | Real-vs-synthetic discrimination / Turing-style task | Classification: AUC | SHAP/LIME/feature attribution |
| Hou and Navarro-Cía, 2023 [82] | CT | Covid-19 [Infectious diseases] | Registration-based image synthesis / Advanced Normalisation Tools; ANT (Scientific and physical simulation models) | Not performed | Not performed | Classification: Accuracy; AUC; Precision / PPV; Recall / sensitivity | Not performed |
| Jang et al., 2023 [83] | X-ray | Normal and abnormal chest x-rays [Pulmonology / respiratory disease] | ABN-PGGAN; NOR-PGGAN; PGGAN; Progressive growing GAN | Not performed | Real-vs-synthetic discrimination / Turing-style task | Classification: Accuracy; F1-score; Precision / PPV; Recall / sensitivity; Specificity | Grad-CAM/CAM family |
| Katakis et al., 2023 [84] | Ultrasound | Muscle thickness [Other] | Denoising Diffusion Probabilistic Model (Diffusion models); Denoising diffusion probabilistic model | FID; LPIPS; PSNR; SSIM | Not performed | Segmentation; Classification; Regression/measurement: Dice; IoU / Jaccard; Precision / PPV; Recall / sensitivity; RMSE | Latent-space visualization or interpretation |
| Khader et al., 2023 [85] | MRI | Radiology [Radiology / medical imaging] | 3D latent diffusion model using VQ-GAN latent space; VQ-GAN + Denoising diffusion probabilistic model; VQ-GAN latent representation plus 3D denoising diffusion probabilistic model | MS-SSIM | Realism or image-quality rating; Clinical plausibility or usefulness rating | Segmentation: Dice | Latent-space visualization or interpretation |
| Khosravi et al., 2023 [86] | X-ray | Hip reconstruction, total joint arthroplasty [Orthopedics / musculoskeletal / rheumatology] | GAN / StyleGANv3; StyleGAN3; StyleGANv3 GAN; StyleGANv3 GAN generating 512 x 512 synthetic AP pelvis images | FID | Real-vs-synthetic discrimination / Turing-style task; Qualitative expert review | Segmentation: Dice | Counterfactual or example-based explanation |
| Lyu et al., 2023 [87] | CT | Breast mass [Oncology] | SEAN (Semantic Region-Adaptive Normalization); Conditional image synthesis / semantic image synthesis | Not performed | Not performed | Classification: Accuracy; F1-score; Recall / sensitivity; Specificity | Not performed |
| Malihi et al., 2023 [88] | Clinical photography | Diabetic foot ulcers and venous leg ulcers [Other] | GAN / StyleGAN3; StyleGAN3; StyleGAN3 GAN | Not performed | Real-vs-synthetic discrimination / Turing-style task; Qualitative expert review | Classification: Accuracy; F1-score; Precision / PPV; Recall / sensitivity | Not performed |
| Menon et al., 2023 [89] | CT | Covid [Pulmonology / respiratory disease] | CycleGAN; Cycle-consistent segmentation GAN / style-transfer GAN | Not performed | Not performed | Classification: Accuracy | Not performed |
| Naga Srinivasu et al., 2023 [90] | MRI | Malignant tissues [Oncology] | VAE | Not performed | Not performed | Classification: Accuracy; F1-score; Recall / sensitivity; Specificity | Not performed |
| Naz et al., 2023 [91] | Fundus photography | Ophtalmology [Ophthalmology] | DCGAN | Not performed | Not performed | Classification: Accuracy; NPV; Precision / PPV; Recall / sensitivity; Specificity | Not performed |
| Nithya et al., 2023 [92] | X-ray | Pneumonia [Pulmonology / respiratory disease] | DCGAN; Multipiled DCGAN (DCGAN) | Not performed | Not performed | Classification: Accuracy; F1-score; Precision / PPV; Recall / sensitivity | Not performed |
| Niu et al., 2023 [93] | Fundus photography | Ophtalmology [Ophthalmology] | Bezier curve-based image synthesis; Procedural synthetic image generator | Not performed | Not performed | Classification: AUPRC; F1-score | Not performed |
| Packhäuser et al., 2023 [94] | X-ray | Pulmonology [Other] | LDM; Latent diffusion model; PGGAN | Not performed | Not performed | Classification: AUC | Not performed |
| Rhyou and Yoo, 2023 [95] | Ultrasound | Cirrhosis [Gastroenterology / hepatology] | Patch-based procedural synthesis; AMP image synthesis | Not performed | Not performed | Classification: Accuracy; Recall / sensitivity; Specificity | Not performed |
| Schaudt et al., 2023 [7] | X-ray | Pneumonia [Pulmonology / respiratory disease] | Unconditional DDPM (Diffusion model); DreamBooth; DreamBooth Stable Diffusion (Diffusion model); DreamBooth; Fine-tuning; GAN; LoRA; Unconditional DDPM; Latent diffusion fine-tuning methods; LoRA; LoRA Stable Diffusion (Diffusion model); Stable Diffusion U-Net (Diffusion model); Diffusion model; Fine-tuning; ... | FID; MS-SSIM | Realism or image-quality rating; Clinical plausibility or usefulness rating | Classification: Accuracy | Not performed |
| Shavlokhova et al., 2023 [96] | Dermoscopy | Skin cancer screening [Oncology] | Fine-tuned GLIDE text-conditional diffusion model with upsampler; GLIDE (fine-tuned diffusion model); Text-conditional diffusion model | FID; IS; MSE; PSNR; SSIM | Real-vs-synthetic discrimination / Turing-style task; Diagnostic interpretation task | Classification: Accuracy; F1-score; Precision / PPV; Recall / sensitivity | Not performed |
| Shen et al., 2023 [97] | X-ray | Lung cancer [Oncology] | DCGAN; GAN-based shape generator plus gated-convolution texture generator | FID; MAE; PSNR; SSIM | Not performed | Classification; Detection / localisation: AUC; Node21 score | Latent-space visualization or interpretation |
| Skandarani et al., 2023 [98] | Multiple imaging techniques | Various [Multiple clinical settings] | Conditional GAN; DCGAN; GAN; GAN with hinge loss; HingeGAN; LSGAN; Least-squares GAN; SPADEGAN; Style-based GAN; StyleGAN; ... | FID | Real-vs-synthetic discrimination / Turing-style task | Segmentation: Dice | Latent-space visualization or interpretation |
| Tsai et al., 2023 [99] | Dermoscopy | Skin cancer [Oncology] | StyleGAN; StyleGAN-derived generator | Not performed | Not performed | Classification: Accuracy; F1-score; Recall / sensitivity | Latent-space visualization or interpretation; Other/unclear |
| Xing et al., 2023 [100] | CT | Medical imaging [Pulmonology / respiratory disease] | V2M2I; V2UM2I; Unsupervised mask-guided conditional GAN; StyleGAN-based mask generator plus pix2pix-style image/segmentation generator | FID; JPEG file size; KL divergence; Standard deviation of extracted features | Not performed | Segmentation: Dice | Not performed |
| Xiong et al., 2023 [101] | CT | Gynecology | Denoising diffusion probabilistic model with adaptive blind denoising and classifier guidance; Class-Guided Diffusion Model; Noise Adaptive Diffusion Model (Post-Adaptive Blind De-noising Module); Noise Adaptive Diffusion Model (Pre-Adaptive Blind De-noising Module) | Not performed | Not performed | Classification: Accuracy; AUC; NPV; Precision / PPV; Recall / sensitivity; Specificity | Not performed |
| Xu et al., 2023 [102] | MRI | Prostate Cancer [Oncology] | SinGAN; Single Natural Image GAN | Not performed | Real-vs-synthetic discrimination / Turing-style task; Realism or image-quality rating | Classification; Segmentation: Accuracy; Dice | Not performed |
| Allan-Blitz et al., 2024 [103] | Clinical photography | Sexually transmitted infections; Human Papillo Virus (HPV) [Infectious diseases] | Text-to-image diffusion model / DDPM; Denoising diffusion probabilistic model using Stable Diffusion plus DreamBooth text-to-image generation; Denoising diffusion probabilistic model using Stable Diffusion, enhanced with DreamBooth personalized text-to-image generation; Stable Diffusion (DDPM-family/ latent diffusion) + DreamBooth (subject-driven fine-tuning, text-to-image personalization) (Diffusion models) | Not performed | Clinical plausibility or usefulness rating | Classification: AUC; F1-score; Precision / PPV; Recall / sensitivity | Not performed |
| Asadi et al., 2024 [104] | MRI | Neurology | GAN / StyleGAN2-ada; StyleGAN2-ada | FID | Not performed | Segmentation: Dice | Latent-space visualization or interpretation |
| Assaf et al., 2024 [105] | CT | Postoperative assessment [Otorhinolaryngology / ENT] | GAN; GAN plus algorithmic image editing | Not performed | Not performed | Regression/measurement: MAE; MAPE; Correlation; RMSE | Not performed |
| Balla et al., 2024 [106] | Ultrasound | Radiology [Radiology / medical imaging] | DDPM; DDPM plus traditional augmentation at runtime; Denoising diffusion probabilistic model | LPIPS; PSNR; SSIM | Not performed | Classification: Accuracy; Recall / sensitivity; Specificity | Grad-CAM/CAM family; Saliency map or heatmap |
| Bradbury et al., 2024 [107] | CT | Radiotherapy planning [Radiology / medical imaging] | Denoising diffusion probabilistic model; Paired multi-network DDPM (paired PET-CT-segmentation); Paired multi-network DDPM with conditional encoders and noise seeding | Not performed | Not performed | Segmentation: Dice | Not performed |
| Chaurasia et al., 2024 [108] | Fundus photography | Ophtalmology [Ophthalmology] | DCGAN; DCGANs; Deep convolutional generative adversarial networks (DCGANs) | Not performed | Qualitative expert review | Classification: Accuracy; AUC; F1-score; Precision / PPV; Recall / sensitivity; Specificity | Grad-CAM/CAM family; Saliency map or heatmap; Latent-space visualization or interpretation |
| Deng et al., 2024 [109] | Endoscopy | Gastric inspection [Other] | STE-StyleGAN; StyleGAN-based image-to-image generative model with Swin Transformer encoder and self-attention generator | FID; LPIPS; PSNR; SSIM | Not performed | Classification: Accuracy; F1-score; Precision / PPV; Recall / sensitivity | Not performed |
| Diamantis et al., 2024 [110] | Endoscopy | Colorectal cancer [Oncology] | CycleGAN; TS-GAN; StyleGANv2; EndoVAE; TIDE; Variational autoencoder (VAE) with multiscale residual architecture | Feature-relative-diversity; Pixel-relative-diversity; Texture-relative-diversity | Real-vs-synthetic discrimination / Turing-style task; Feature identification or annotation; Qualitative expert review | Classification: AUC | Grad-CAM/CAM family; Expert review of explanation maps |
| Gonzalez-Perez et al., 2024 [111] | Clinical photography | Kidney stone [Nephrology] | DDPM; SinDDM; Single Image Denoising Diffusion Model | Brightness drift; Mean blue relative intensity drift; Mean green relative intensity drift; Mean red relative intensity drift; Rms contrast drift; SIFID | Not performed | Classification: Accuracy | Not performed |
| Gurusubramani S. and Latha B., 2024 [112] | MRI | Heart disease diagnosis; Cardiac arrhythmia [Cardiology / cardiovascular disease] | DCGAN | MSE; PSNR; SSIM | Not performed | Classification; Detection / localisation; Regression/measurement: Accuracy; AUC; AUPRC; F1-score; False negatives; False positives; MSE; Precision / PPV; Recall / sensitivity; Specificity | Not performed |
| Hossain et al., 2024 [113] | MRI | Brain Tumor [Oncology] | ACGAN (BrainACGAN); Auxiliary Classifier GAN | Not performed | Not performed | Classification: Accuracy; F1-score | Not performed |
| Islam et al., 2024 [114] | Dermoscopy | Dermatology | GAN / StyleGAN2-ADA; StyleGAN2-ADA + cosine similarity-based image filtering (Style-based GANs) | Not performed | Not performed | Classification: Accuracy; F1-score; Recall / sensitivity; Specificity | Latent-space visualization or interpretation |
| Kebaili et al., 2024 [115] | OCT | Retinopathy of prematurity [Ophthalmology] | Discriminative Hamiltonian Variational Autoencoder (dHVAE); VAE | FID; LPIPS; PSNR | Not performed | Segmentation: Dice | Ablation/perturbation analysis; Qualitative realism and artefact inspection; Distributional validation of synthetic masks |
| Khosravi et al., 2024 [116] | X-ray | Radiology [Radiology / medical imaging] | Denoising Diffusion Probabilistic Model (DDPM); Denoising diffusion probabilistic model | FID | Not performed | Classification: AUC | Not performed |
| Kikuchi et al., 2024 [117] | X-ray | Respiratory diseases [Pulmonology / respiratory disease] | Auto-encoding GAN plus conditional tabular GAN; PCA, AE, and αGAN encode/decode reconstruction models; αGAN; αGAN + CTGAN (VAE-GAN hybrids) | MSE; PSNR; SSIM | Preference or ranking task | Classification: AUC | Not performed |
| Kumar et al., 2024 [118] | Fundus photography | Retinopathy [Ophthalmology] | Conditional GAN / DCGAN; DCGAN | FID | Not performed | Classification: Accuracy; F1-score; Precision / PPV; Recall / sensitivity | Not performed |
| Moon et al., 2024 [119] | MRI | Oncology | Diffusion model; Score-based diffusion model; Score-based diffusion model used for generative AI-based augmentation | FID | Real-vs-synthetic discrimination / Turing-style task; Diagnostic interpretation task; Feature identification or annotation | Classification: Accuracy; F1-score; Precision / PPV; Recall / sensitivity; Specificity | Grad-CAM/CAM family; Saliency map or heatmap |
| Oliveira et al., 2024 [120] | Fundus photography | Ophthalmology | GAN; StyleGAN2-ADA | FID | Real-vs-synthetic discrimination / Turing-style task; Diagnostic interpretation task | Classification: Accuracy | Grad-CAM/CAM family; Saliency map or heatmap |
| Park et al., 2024 [121] | CT | Bone marrow edema [Hematology] | W-DRAG (WGAN-GP); WGAN-GP with data random augmentation optimized for generative networks | FID | Not performed | Classification: Accuracy; AUC; F1-score; Recall / sensitivity; Specificity | Grad-CAM/CAM family; Latent-space visualization or interpretation |
| Patil et al., 2024a [122] | MRI | Alzheimer's disease [Neurology] | DCGAN; WGAN; PGGAN; PGGAN (PGGAN) | SSIM | Not performed | Classification: Accuracy | Not performed |
| Patil et al., 2024b [123] | Clinical photography | Skin cancer [Oncology] | DCGAN; WGAN; PGGAN; PGGAN (PGGAN) | SSIM | Not performed | Classification: Accuracy; F1-score; Precision / PPV; Recall / sensitivity | Not performed |
| Saihood et al., 2024 [124] | CT | Lung cancer [Oncology] | DDDG-GAN; Dual-discriminator dual-generator GAN | Predicted class confidence | Not performed | Classification: Accuracy; AUC; F1-score; Precision / PPV; Recall / sensitivity | Grad-CAM/CAM family; SHAP/LIME/feature attribution; Latent-space visualization or interpretation |
| Shakiruzzaman et al., 2024 [125] | X-ray | pneumonia [Pulmonology / respiratory disease] | ACGAN (Auxiliary Classifier GAN); Auxiliary Classifier GAN | Not performed | Not performed | Classification: Accuracy; F1-score; Precision / PPV; Recall / sensitivity | Not performed |
| Xu et al., 2024a [126] | Ultrasound | Breast imaging [Other] | CoLDiT; Conditional latent diffusion model with diffusion transformer backbone; Stable Diffusion fine-tuning / text-to-image latent diffusion; TDASD (Stable Diffusion, Ksample) (Diffusion models.text-to-image) | FID; IS; Pixel grayscale mean; Pixel grayscale standard deviation | Real-vs-synthetic discrimination / Turing-style task; Diagnostic interpretation task; Other structured reader endpoint | Classification: AUC; F1-score; Recall / sensitivity; Specificity | Not performed |
| Xu et al., 2024b [127] | CT | Lung adenocarcinoma [Oncology] | CoLDiT; Conditional latent diffusion model with diffusion transformer backbone; Stable Diffusion fine-tuning / text-to-image latent diffusion; TDASD (Stable Diffusion, Ksample) (Diffusion models.text-to-image) | FID; IS; Pixel grayscale mean; Pixel grayscale standard deviation | Not performed | Classification: Accuracy; AUC; F1-score; Recall / sensitivity | Not performed |
| Yalcin et al., 2024 [128] | CT | Stroke [Neurology] | Image deformation / lesion-variation synthesis; Synthetic CT image generation | Not performed | Not performed | Classification: Accuracy; F1-score; Recall / sensitivity; Specificity | Attention map or attention rollout |
| Zhang et al., 2024 [129] | Ultrasound | Breast cancer [Oncology] | Procedural synthetic image and mask generation / copy-paste with blending; Tumor-region embedding into normal BUS images + edge blending (Scientific and physical simulation model) | Not performed | Not performed | Classification; Segmentation: F1-score; IoU / Jaccard | Not performed |
| Zheng et al., 2024 [130] | CT | Hepatocellular carcinoma (HCC) [Oncology] | LF-GAN (Loss-based GAN); Light & Fast Generative Adversarial Network | FID; PSNR; SSIM | Not performed | Classification: Accuracy; AUC; Recall / sensitivity; Specificity | Not performed |
| Zhou et al., 2024 [131] | Clinical photography | Dermatology | Procedural multi-view image synthesis / 3D reconstruction and view interpolation; 3D reconstruction and view interpolation-based data augmentation | Not performed | Not performed | Segmentation: Dice; IoU / Jaccard; Recall / sensitivity | Saliency map or heatmap; Other/unclear |
| dos Reis Carvalho et al., 2024 [132] | Fundus photography | Other | Rule-based/geometric synthetic image generation using manually annotated vessel texture maps, Bézier curves, Delaunay triangulation, and piecewise affine transformations; Texture-map + Bézier vessel generator (Scientific/physical simulation model) | Not performed | Not performed | Segmentation: Dice | Not performed |
| A Hasib et al., 2025 [133] | X-ray | COVID-19 and pneumonia [Pulmonology / respiratory disease] | FILM + PCA (Feature Interpolation through Linear Mapping + Principal Component Analysis) (other); Frame interpolation plus PCA augmentation | Not performed | Not performed | Classification: Accuracy; F1-score; Precision / PPV; Recall / sensitivity | Grad-CAM/CAM family; Saliency map or heatmap |
| Almuhaimeed et al., 2025 [134] | MRI | Brain Tumor [Oncology] | cGAN (AE-cGAN augmentation); Autoencoder plus conditional GAN | Not performed | Not performed | Classification: Accuracy; F1-score; Recall / sensitivity | Not performed |
| Bharath et al., 2025 [135] | MRI | Alzheimer severity classification [Neurology] | DCGAN; GAN | SSIM | Not performed | Classification: Accuracy; F1-score; Precision / PPV; Recall / sensitivity | SHAP feature attribution; Pixel-wise attribution maps; ROI-level feature-importance aggregation; Synthetic-image anatomical validation |
| Brehmer et al., 2025 [136] | Clinical photography | Dermatology | Diffusion model / Stable Diffusion; Stable Diffusion (fine-tuned synthetic wound image generation) | Not performed | Not performed | Classification: F1-score | Not performed |
| Dahan et al., 2025 [137] | Ultrasound | Orthopedics / musculoskeletal / rheumatology | CSG; CSG (Context-Semantic Guidance); CSG (Context-Semantic Guidance) diffusion model; GAN | Contour F1 score; Contour IoU; Contour PPV; Contour TPR; FID; KL divergence; KST | Real-vs-synthetic discrimination / Turing-style task | Segmentation: Dice | Controllable semantic and context conditioning analysis; Latent-space visualization; Visual Turing Test; Clinical expert realism review; Distribution-overlap validation; Downstream segmentation validation |
| Dai et al., 2025 [138] | Ultrasound | Oncology | Tiger-F; Tiger-F (Feature-Guided); Text-guided diffusion model based on Stable Diffusion and ControlNet-style foreground/background encoders; Tiger-F; Stable Diffusion; Imagen; PG-GAN; Diffusion Transformers; Imagen; Stable Diffusion; Tiger-F; PG-GAN; Diffusion Transformers; Imagen; Stable Diffusion | CMMD; Density and coverage; GS; SSIM | Real-vs-synthetic discrimination / Turing-style task; Feature identification or annotation; Text-image matching | Classification: AUC; Recall / sensitivity; Specificity | Not performed |
| Ersarı et al., 2025 [139] | OCT | Cataracts [Ophthalmology] | DD-GAN; diffusion/GAN hybrid | FID; IS; SSIM | Not performed | Classification: Accuracy; F1-score; Precision / PPV; Recall / sensitivity | Attention-map visualization; Clinical expert realism review; Distributional realism validation; Task-based synthetic-data validation |
| Fukae et al., 2025 [140] | Ultrasound | Rheumatology [Orthopedics / musculoskeletal / rheumatology] | Artificial illustrated ultrasound images (manual digital illustration) | Not performed | Not performed | Classification: AUC | Not performed |
| Grabke et al., 2025 [141] | MRI | Prostate cancer [Oncology] | CCELLA; CCELLA and comparator latent diffusion models; progressive auxiliary-classifier GAN | FID | Not performed | Classification: Accuracy; AUC; Precision / PPV; Recall / sensitivity; Specificity; NPV | Conditioning-mechanism ablation; Qualitative anatomical and artefact comparison; Text-class alignment analysis; Downstream clinical-task validation |
| Guo et al., 2025 [142] | X-ray | Pulmonology / respiratory disease | CPDM; conditional attentional GAN | FID; Generative precision; Generative recall; IS | Not performed | Classification: AUC | Component ablation; Qualitative class-specific lesion comparison; Class-prototype and latent-interaction analysis; Downstream tail-class validation |
| Hosseini and Serag, 2025 [143] | X-ray | Other | DDPM (diffusion model with Swin-transformer); Denoising diffusion probabilistic model | FID; IS | Not performed | Classification: Accuracy; AUC; F1-score; Precision / PPV; Recall / sensitivity | Latent-space visualization or interpretation |
| Jeyasri et al., 2025 [144] | Fundus photography | Retinal disease classification [Ophthalmology] | latent diffusion transformer; ResNet-based generative adversarial network (RGAN); ResNet-based generative adversarial network (RGAN; ResNet-50 generator) | Not performed | Not performed | Classification: Accuracy; F1-score; Recall / sensitivity; Specificity | Not performed |
| Khazrak et al., 2025 [145] | Endoscopy | laryngology [Otorhinolaryngology / ENT] | DDPM; Denoising Diffusion Probabilistic Model (DDPM) with U-Net architecture; Denoising diffusion probabilistic model | FID | Realism or image-quality rating; Clinical plausibility or usefulness rating; Qualitative expert review | Classification: Accuracy; F1-score; Recall / sensitivity | Not performed |
| Kim et al., 2025 [146] | Dermoscopy | Dermatology | Latent diffusion model / Stable Diffusion-based generative model; Improved Diffusion Model (8ch VAE + multi-level embeddings) | FID; IS; LPIPS; MS-SSIM; MSE | Not performed | Classification: Accuracy | Latent-space visualization or interpretation |
| King et al., 2025 [147] | MRI | Schizophrenia [Neurology] | α-SN-GAN with auxiliary classifier; semi-supervised GAN/autoencoder | Not performed | Not performed | Classification: Accuracy | Systematic architecture comparison; Qualitative artefact inspection; PCA latent-distribution visualization; Conditional-generation validation; Downstream diagnostic validation |
| Lai et al., 2025 [148] | MRI | Alzheimer's disease [Neurology] | PACGAN; StyleGAN plus latent diffusion | FID; KID; SSIM | Not performed | Classification: AUC; AUC (cross-validation) | Distributional and mode-collapse validation; Conditional discriminator validation; Qualitative realism comparison; Cross-dataset downstream validation |
| Lajous et al., 2025 [149] | MRI | fetal neuroimaging [Neurology] | FaBiAN v1.2; FaBiAN v2.0; FaBiAN v2.0 numerical fetal brain MRI simulation framework; FaBiAN v2.0 numerical fetal brain MRI simulation framework; comparator FaBiAN v1.2; Physics-guided numerical phantom / MRI simulation | Not performed | Realism or image-quality rating; Preference or ranking task | Segmentation: Dice | Not performed |
| Lyu and Tian, 2025 [150] | MRI | Brain tumor [Oncology] | WGAN; Wasserstein GAN integrated with U-Net/Transformer segmentation framework | Not performed | Not performed | Segmentation: Dice | Not performed |
| Mizoguchi et al., 2025 [151] | Clinical photography | Clinical ENT [Otorhinolaryngology / ENT] | GAN; StyleGAN3 | FID | Clinical plausibility or usefulness rating; Qualitative expert review | Classification: Accuracy | Not performed |
| Parida et al., 2025 [152] | Multiple imaging techniques | Tumor segmentation [Oncology] | DCGAN; GAN | IS; PSNR; SSIM | Qualitative expert review | Classification; Segmentation: Accuracy; F1-score; Precision / PPV; Recall / sensitivity; Specificity; NPV; Dice | Expert realism review; Qualitative synthetic-versus-real comparison; Component ablation |
| Satapathy et al., 2025 [153] | CT | Tumor segmentation [Oncology] | GAN | FID | Not performed | Classification: Accuracy; AUC; F1-score; Precision / PPV; Recall / sensitivity | Gradient-based feature attribution; Class activation mapping; Clinical expert review of explanation maps |
| Shi et al., 2025 [154] | Ultrasound | Breast lesions, Thyroid nodules [Oncology] | 3D latent diffusion model; SIA-GAN; Semantic Information guided Attention GAN (SIA-GAN) | FID; LPIPS; PSNR; SSIM | Not performed | Segmentation: Dice; IoU / Jaccard | Architecture-component ablation; Qualitative artefact analysis; Controllable semantic synthesis; Hyperparameter perturbation analysis; Downstream segmentation validation |
| Shin et al., 2025 [155] | CT | Gastrointestinal [Gastroenterology / hepatology] | GAN / image translation model; Proposed size-controllable tumor synthesis method based on ACL-GAN; modified ACL-GAN with explicit tumor size control | Chi-square distance; FID; KID | Real-vs-synthetic discrimination / Turing-style task | Detection / localisation: AUC; Recall / sensitivity | Not performed |
| Sujini et al., 2025 [156] | Ultrasound | Tyroid nodules classification [Oncology] | GAN; WGAN-GP; Wasserstein GAN with Gradient Penalty (WGAN-GP) | Not performed | Qualitative expert review | Classification: Accuracy; F1-score; Precision / PPV; Recall / sensitivity; Specificity | Training-dynamics visualization; Clinical review of synthetic-image realism |
| Sukegawa et al., 2025 [157] | X-ray | Odontology [Dental medicine] | Artificial X-ray implant image generation from 3D surface models; Artificial X-ray implant image generation from 3D surface models with background processing; Physics/procedural image simulation | Not performed | Not performed | Classification: Accuracy; AUC; F1-score; Precision / PPV; Recall / sensitivity | Latent-space visualization or interpretation |
| Tan et al., 2025 [158] | Clinical photography | Ophthalmology [Ophthalmology] | StyleGAN2; StyleGAN2 (FFHQ pretrained) | FID | Not performed | Classification: Accuracy; AUC; NPV; Precision / PPV; Recall / sensitivity; Specificity | Not performed |
| Wang et al., 2025a [159] | X-ray | Other | Latent stable diffusion / medical image-text generative foundation model; MINIM; MINIM after reinforcement learning; MINIM with reinforcement learning from human/clinician feedback; MINIM; StyleGAN-T; GigaGAN; DALLE; Imagen; StyleGAN-T; Comparator text-to-image generative models; Generative adversarial network (GAN); StyleGAN2-ADA | FID; IS; MS-SSIM; KID | Realism or image-quality rating; Clinical plausibility or usefulness rating | Classification: Accuracy | Not performed |
| Wang et al., 2025b [160] | Clinical photography | Microbial keratitis [Ophthalmology] | Latent stable diffusion / medical image-text generative foundation model; MINIM; MINIM after reinforcement learning; MINIM with reinforcement learning from human/clinician feedback; MINIM; StyleGAN-T; GigaGAN; DALLE; Imagen; StyleGAN-T; Comparator text-to-image generative models; Generative adversarial network (GAN); StyleGAN2-ADA | FID; IS; MS-SSIM; KID | Real-vs-synthetic discrimination / Turing-style task; Realism or image-quality rating; Clinical plausibility or usefulness rating; Diagnostic interpretation task | Classification: Accuracy; Recall / sensitivity; Specificity | Not performed |
| Xu et al., 2025 [161] | MRI | Brain tumor [Oncology] | LF-SynthSeg tumor synthesis; Procedural / tissue-prior-based synthetic tumor generation with segmentation network training | Entropy | Not performed | Segmentation: Dice | Not performed |
| Ye et al., 2025 [162] | X-ray | Pneumonia, Skin lesion, Polyp detection [Pulmonology / respiratory disease] | Latent Diffusion Model (LDM); Latent diffusion model / Stable Diffusion-like model | Not performed | Not performed | Classification; Segmentation: Accuracy; Recall / sensitivity; Dice; IoU / Jaccard | Not performed |
| Yu et al., 2025 [163] | X-ray | Odontology [Dental medicine] | ToothMaker; Stable Diffusion-based dental radiograph generator | FID; IS; MS-SSIM | Realism or image-quality rating; Clinical plausibility or usefulness rating; Feature identification or annotation | Segmentation: Dice; IoU / Jaccard | Not performed |
| Zafar et al., 2025 [164] | MRI | Neuro-oncology diagnostics [Oncology] | Dual-Stream Contrastive Latent Projection GAN (Conditional and guided GANs); Dual-stream contrastive latent projection GAN | FID; PSNR; SSIM | Not performed | Classification: Accuracy | Latent-space visualization or interpretation |
| Zeng et al., 2025 [165] | CT | Other | Auxiliary classifier GAN / ReACGAN; ReACGAN; StyleGAN2; StyleGAN2 (expert-guided via GANSpace); StyleGAN2; ReACGAN | MMD; SSIM | Realism or image-quality rating; Clinical plausibility or usefulness rating | Classification: AUC; AUPRC; F1-score | Grad-CAM/CAM family; Latent-space visualization or interpretation |
| Zhang et al., 2025 [166] | MRI | Brain tumour; Ischemic stroke [Neurology] | SynthTumour | FID; MMD; t-SNE mean distance | Not performed | Classification; Segmentation: Precision / PPV; Recall / sensitivity; Dice | Latent-space visualization or interpretation |
| Zhou et al., 2025 [167] | MRI | Oncology | 3D-Med-DDPM; 3D-VQGAN-lat8; Vector-quantized GAN plus transformer; 3D-WGAN-GP; 3D-αWGAN; VQGAN + Diffusion | 3D-MMD; 3D-SSIM; FID; MS-SSIM; PSNR | Not performed | Classification: Accuracy; AUC; F1-score | Not performed |

## Table S9. Frequency of canonical generation model families and subtypes across the 160 included studies.

| Generation model family | Canonical model subtype | Reported studies, n (% of all included studies) |
| --- | --- | --- |
| **GAN-based** |  | **119 (74.4%)** |
|  | **Other GAN architecture** | 53 (33.1%) |
|  | **DCGAN family** | 30 (18.8%) |
|  | **StyleGAN family** | 23 (14.4%) |
|  | **Conditional / auxiliary-classifier GAN** | 18 (11.2%) |
|  | **Progressive-growing GAN** | 16 (10.0%) |
|  | **Wasserstein GAN family** | 12 (7.5%) |
|  | **CycleGAN family** | 5 (3.1%) |
|  | **Pix2Pix family** | 3 (1.9%) |
| **Diffusion models** |  | **28 (17.5%)** |
|  | **DDPM and task-specific diffusion** | 16 (10.0%) |
|  | **Latent / Stable Diffusion** | 15 (9.4%) |
|  | **Other diffusion architecture** | 6 (3.8%) |
|  | **Score-based diffusion** | 1 (0.6%) |
| **Hybrid models** |  | **15 (9.4%)** |
|  | **VAE-GAN hybrid** | 8 (5.0%) |
|  | **Diffusion-GAN / VQGAN hybrid** | 5 (3.1%) |
|  | **Other hybrid architecture** | 2 (1.2%) |
| **Procedural / statistical / geometric synthesis** |  | **10 (6.2%)** |
| **Scientific / physical simulation** |  | **7 (4.4%)** |
| **Registration / deformation / interpolation** |  | **5 (3.1%)** |
| **VAE / autoencoder** |  | **4 (2.5%)** |
| **Other neural synthesis** |  | **2 (1.2%)** |

## Table S10. Frequency of canonical human-reader endpoint/task categories.

Studies were grouped using the canonical human-reader endpoint/task taxonomy in Table S2. Categories are not mutually exclusive. Percentages are reported among the 50 reader studies and among all 160 included studies.

| Canonical human-reader endpoint/task category | Studies, n (% of 50 reader studies) | Studies, n (% of 160 included studies) |
| --- | --- | --- |
| **Real-vs-synthetic discrimination / Turing-style task** | 31 (62.0%) | 31 (19.4%) |
| **Realism or image-quality rating** | 14 (28.0%) | 14 (8.8%) |
| **Clinical plausibility or usefulness rating** | 11 (22.0%) | 11 (6.9%) |
| **Preference or ranking task** | 3 (6.0%) | 3 (1.9%) |
| **Diagnostic interpretation task** | 9 (18.0%) | 9 (5.6%) |
| **Feature identification or annotation** | 12 (24.0%) | 12 (7.5%) |
| **Text-image matching** | 1 (2.0%) | 1 (0.6%) |
| **Qualitative expert review** | 14 (28.0%) | 14 (8.8%) |
| **Other structured reader endpoint** | 1 (2.0%) | 1 (0.6%) |

## Table S11. Frequency and quantitative summary of canonical human-reader metrics.

Only prespecified canonical metrics are included in the main summary. Percentages are reported among 50 reader studies and all 160 included studies. Study-specific narrative labels remain available in the audit sheet of the source workbook and are not pooled across incompatible definitions or scales.

| Endpoint/task category | Canonical metric | Reported studies, n (% reader studies) | Reported studies, n (% total studies) | Reported records, n | Numeric studies, n (% reader studies) | Median [Q1, Q3] | Range |
| --- | --- | --- | --- | --- | --- | --- | --- |
| **Real-vs-synthetic discrimination / Turing-style task** | **Turing accuracy** | 27 (54.0%) | 27 (16.9%) | 74 | 27 (54.0%) | 55.1 [50.0, 61.9] | 24.8–100.0 |
|  | **Turing sensitivity** | 13 (26.0%) | 13 (8.1%) | 30 | 13 (26.0%) | 59.5 [50.4, 70.4] | 1.00–98.3 |
|  | **Turing specificity** | 13 (26.0%) | 13 (8.1%) | 30 | 13 (26.0%) | 48.5 [37.1, 59.7] | 1.00–86.7 |
|  | **Real authenticity rate** | 9 (18.0%) | 9 (5.6%) | 19 | 9 (18.0%) | 74.0 [69.0, 82.5] | 56.4–98.0 |
|  | **Synthetic authenticity rate** | 7 (14.0%) | 7 (4.4%) | 12 | 7 (14.0%) | 57.0 [40.5, 63.0] | 31.0–92.2 |
|  | **Turing AUC** | 3 (6.0%) | 3 (1.9%) | 5 | 3 (6.0%) | 0.597 [0.592, 0.960] | 0.020–1.00 |
|  | **Turing sensitivity / Turing specificity / Turing AUC** | 1 (2.0%) | 1 (0.6%) | 1 | 1 (2.0%) | 0.297 [0.297, 0.297] | 0.297–0.297 |
| **Realism or image-quality rating** | **Quality Likert score** | 4 (8.0%) | 4 (2.5%) | 12 | 4 (8.0%) | 2.47 [1.56, 4.09] | 1.00–96.0 |
|  | **Realism Likert score** | 3 (6.0%) | 3 (1.9%) | 5 | 3 (6.0%) | 4.55 [3.53, 27.5] | 1.20–95.5 |
|  | **Artefact Likert score** | 1 (2.0%) | 1 (0.6%) | 4 | 1 (2.0%) | 98.0 [96.5, 98.5] | 92.0–100.0 |
| **Interrater agreement** | **Interrater agreement** | 12 (24.0%) | 12 (7.5%) | 24 | 11 (22.0%) | 0.460 [0.090, 0.670] | -0.247–100.0 |
| **Diagnostic interpretation task** | **Reader diagnostic accuracy** | 6 (12.0%) | 6 (3.8%) | 24 | 6 (12.0%) | 82.1 [79.9, 93.7] | 40.0–98.0 |
|  | **Reader diagnostic AUC** | 4 (8.0%) | 4 (2.5%) | 12 | 4 (8.0%) | 0.760 [0.736, 0.797] | 0.020–0.864 |
|  | **Reader diagnostic sensitivity** | 4 (8.0%) | 4 (2.5%) | 8 | 4 (8.0%) | 97.5 [95.6, 98.0] | 80.0–100.0 |
|  | **Reader diagnostic specificity** | 4 (8.0%) | 4 (2.5%) | 8 | 4 (8.0%) | 90.0 [74.2, 96.8] | 50.0–98.0 |
| **Feature identification or annotation** | **Feature identification accuracy** | 4 (8.0%) | 4 (2.5%) | 11 | 3 (6.0%) | 75.8 [16.6, 83.8] | -0.310–94.0 |
| **Clinical plausibility or usefulness rating** | **Clinical plausibility Likert score** | 2 (4.0%) | 2 (1.2%) | 6 | 2 (4.0%) | 2.01 [1.45, 2.19] | 1.06–90.8 |
|  | **Pathology realism Likert score** | 1 (2.0%) | 1 (0.6%) | 12 | 1 (2.0%) | 4.06 [3.89, 4.28] | 1.28–4.41 |
| **Preference or ranking task** | **Ranking score** | 2 (4.0%) | 2 (1.2%) | 2 | 1 (2.0%) | 25.0 [25.0, 25.0] | 25.0–25.0 |
|  | **Preference rate** | 1 (2.0%) | 1 (0.6%) | 4 | 1 (2.0%) | 50.0 [2.55, 97.4] | 0.000–100.0 |
| **Text-image matching** | **Text-image matching accuracy** | 1 (2.0%) | 1 (0.6%) | 2 | 1 (2.0%) | 89.1 [88.2, 90.0] | 87.3–91.0 |

## Table S12. Frequency of canonical automated image-quality metric families.

Only studies reporting canonical quantitative automated image-quality metrics were included in this summary.

| Automated metric family | Reported studies, n (% of 93 automated-IQM studies) | Reported records, n | Boxplot-representable studies, n (%) | Boxplot-representable records, n |
| --- | --- | --- | --- | --- |
| **Distributional similarity or fidelity** | 68 (73.1%) | 160 | 66 (71.0%) | 150 |
| **Pixel/structural similarity** | 34 (36.6%) | 66 | 34 (36.6%) | 66 |
| **Automated realism/no-reference quality** | 23 (24.7%) | 47 | 23 (24.7%) | 41 |
| **Reconstruction error** | 22 (23.7%) | 43 | 22 (23.7%) | 43 |
| **Diversity or coverage** | 10 (10.8%) | 26 | 11 (11.8%) | 27 |
| **Perceptual similarity** | 8 (8.6%) | 17 | 8 (8.6%) | 17 |
| **Other automated image-quality metric** | 8 (8.6%) | 17 | 7 (7.5%) | 14 |
| **Intensity, texture, and image-statistical descriptors** | 4 (4.3%) | 14 | 4 (4.3%) | 14 |
| **Privacy/memorization similarity** | 1 (1.1%) | 1 | 0 (0.0%) | 0 |

## Table S13. Frequency and quantitative summary of canonical automated image-quality metrics.

| Automated metric family | Canonical metric | Direction | Reported studies, n (%) | Reported records, n | Boxplot-representable studies, n (%) | Boxplot-representable records, n | Median [Q1, Q3] | Minimum | Maximum |
| --- | --- | --- | --- | --- | --- | --- | --- | --- | --- |
| **Automated realism/no-reference quality** | **IS** | Higher is better | 19 (20.4%) | 39 | 19 (20.4%) | 35 | 2.09 [1.81, 2.85] | 1.23 | 9.50 |
|  | **SNR** | Higher is better | 2 (2.2%) | 3 | 2 (2.2%) | 2 | 14.1 [7.89, 20.3] | 1.70 | 26.5 |
|  | **Predicted class confidence** | Higher is better | 1 (1.1%) | 2 | 1 (1.1%) | 2 | 92.5 [91.2, 93.8] | 90.0 | 95.0 |
|  | **SNR difference** | Lower is better | 1 (1.1%) | 1 | 1 (1.1%) | 1 | 1.50 [1.50, 1.50] | 1.50 | 1.50 |
|  | **Discriminator score** | Unclear / target-dependent | 1 (1.1%) | 1 | 0 (0.0%) | 0 | - | - | - |
|  | **Generator score** | Unclear / target-dependent | 1 (1.1%) | 1 | 0 (0.0%) | 0 | - | - | - |
|  | **CNN conventional-vs-synthetic classification accuracy** | Lower is better | 0 (0.0%) | 0 | 1 (1.1%) | 1 | 76.4 [76.4, 76.4] | 76.4 | 76.4 |
| **Distributional similarity or fidelity** | **FID** | Lower is better | 60 (64.5%) | 124 | 59 (63.4%) | 116 | 46.9 [7.97, 96.6] | 0.001 | 227.3 |
|  | **KL divergence** | Lower is better | 4 (4.3%) | 9 | 4 (4.3%) | 9 | 1.01 [0.733, 1.11] | 0.065 | 2.21 |
|  | **MMD** | Lower is better | 4 (4.3%) | 9 | 4 (4.3%) | 8 | 0.035 [0.020, 0.097] | 0.001 | 0.246 |
|  | **KID** | Lower is better | 4 (4.3%) | 7 | 3 (3.2%) | 6 | 0.126 [0.013, 2.05] | 0.000 | 8.04 |
|  | **SIFID** | Lower is better | 2 (2.2%) | 3 | 2 (2.2%) | 3 | 3.14 [1.68, 3.46] | 0.220 | 3.79 |
|  | **CMMD** | Lower is better | 1 (1.1%) | 3 | 1 (1.1%) | 3 | 0.320 [0.270, 0.365] | 0.220 | 0.410 |
|  | **3D-MMD** | Lower is better | 1 (1.1%) | 1 | 1 (1.1%) | 1 | 14,982 [14,982, 14,982] | 14,982 | 14,982 |
|  | **Jensen-Shannon divergence** | Lower is better | 1 (1.1%) | 1 | 1 (1.1%) | 1 | 3.30 [3.30, 3.30] | 3.30 | 3.30 |
|  | **KST** | Lower is better | 1 (1.1%) | 1 | 1 (1.1%) | 1 | 0.025 [0.025, 0.025] | 0.025 | 0.025 |
|  | **SWD** | Lower is better | 1 (1.1%) | 1 | 1 (1.1%) | 1 | 25.0 [25.0, 25.0] | 25.0 | 25.0 |
|  | **Wasserstein distance** | Lower is better | 1 (1.1%) | 1 | 1 (1.1%) | 1 | 0.123 [0.123, 0.123] | 0.123 | 0.123 |
| **Diversity or coverage** | **Generative precision** | Higher is better | 3 (3.2%) | 5 | 3 (3.2%) | 5 | 0.661 [0.525, 0.662] | 0.523 | 0.717 |
|  | **Generative recall** | Higher is better | 3 (3.2%) | 5 | 3 (3.2%) | 5 | 0.334 [0.285, 0.433] | 0.220 | 0.701 |
|  | **Entropy** | Higher is better | 2 (2.2%) | 3 | 2 (2.2%) | 3 | 2.49 [2.46, 4.76] | 2.43 | 7.03 |
|  | **Density and coverage** | Unclear / target-dependent | 1 (1.1%) | 3 | 1 (1.1%) | 3 | 0.170 [0.125, 0.195] | 0.080 | 0.220 |
|  | **JPEG file size** | Lower is better | 1 (1.1%) | 2 | 1 (1.1%) | 2 | 313.0 [313.0, 313.0] | 313.0 | 313.0 |
|  | **Recall for generative-model diversity proxy** | Higher is better | 1 (1.1%) | 2 | 1 (1.1%) | 2 | 95.5 [93.8, 97.2] | 92.0 | 99.0 |
|  | **Standard deviation of extracted features** | Higher is better | 1 (1.1%) | 2 | 1 (1.1%) | 2 | 0.085 [0.082, 0.088] | 0.080 | 0.090 |
|  | **Feature-relative diversity** | Unclear / target-dependent | 1 (1.1%) | 1 | 1 (1.1%) | 1 | 0.515 [0.515, 0.515] | 0.515 | 0.515 |
|  | **MODE score** | Higher is better | 1 (1.1%) | 1 | 1 (1.1%) | 1 | 1.15 [1.15, 1.15] | 1.15 | 1.15 |
|  | **Pixel-relative diversity** | Unclear / target-dependent | 1 (1.1%) | 1 | 1 (1.1%) | 1 | 0.765 [0.765, 0.765] | 0.765 | 0.765 |
|  | **Texture-relative diversity** | Unclear / target-dependent | 1 (1.1%) | 1 | 1 (1.1%) | 1 | 0.985 [0.985, 0.985] | 0.985 | 0.985 |
|  | **PRD-AUC** | Higher is better | 0 (0.0%) | 0 | 1 (1.1%) | 1 | 0.838 [0.838, 0.838] | 0.838 | 0.838 |
| **Intensity, texture, and image-statistical descriptors** | **Brightness drift** | Lower is better | 1 (1.1%) | 2 | 1 (1.1%) | 2 | 0.140 [0.138, 0.141] | 0.137 | 0.142 |
|  | **Mean blue relative-intensity drift** | Lower is better | 1 (1.1%) | 2 | 1 (1.1%) | 2 | 0.093 [0.092, 0.093] | 0.092 | 0.094 |
|  | **Mean green relative-intensity drift** | Lower is better | 1 (1.1%) | 2 | 1 (1.1%) | 2 | 0.084 [0.069, 0.098] | 0.055 | 0.112 |
|  | **Mean red relative-intensity drift** | Lower is better | 1 (1.1%) | 2 | 1 (1.1%) | 2 | 0.068 [0.064, 0.073] | 0.060 | 0.077 |
|  | **RMS contrast drift** | Lower is better | 1 (1.1%) | 2 | 1 (1.1%) | 2 | 0.135 [0.129, 0.140] | 0.124 | 0.145 |
|  | **Pixel grayscale mean** | Unclear / target-dependent | 1 (1.1%) | 1 | 1 (1.1%) | 1 | 118.8 [118.8, 118.8] | 118.8 | 118.8 |
|  | **Pixel grayscale standard deviation** | Unclear / target-dependent | 1 (1.1%) | 1 | 1 (1.1%) | 1 | 70.8 [70.8, 70.8] | 70.8 | 70.8 |
|  | **Skewness** | Unclear / target-dependent | 1 (1.1%) | 1 | 1 (1.1%) | 1 | 4.70 [4.70, 4.70] | 4.70 | 4.70 |
|  | **Vessel amount difference** | Lower is better | 1 (1.1%) | 1 | 1 (1.1%) | 1 | 0.430 [0.430, 0.430] | 0.430 | 0.430 |
| **Other automated image-quality metric** | **GS** | Unclear / target-dependent | 1 (1.1%) | 3 | 1 (1.1%) | 3 | 0.001 [0.001, 0.001] | 0.001 | 0.001 |
|  | **Precision for generative-model quality proxy** | Higher is better | 1 (1.1%) | 2 | 1 (1.1%) | 2 | 96.0 [94.5, 97.5] | 93.0 | 99.0 |
|  | **Chi-square distance** | Higher is better | 1 (1.1%) | 1 | 1 (1.1%) | 1 | 83.2 [83.2, 83.2] | 83.2 | 83.2 |
|  | **Contour F1 score** | Higher is better | 1 (1.1%) | 1 | 1 (1.1%) | 1 | 0.480 [0.480, 0.480] | 0.480 | 0.480 |
|  | **Contour IoU** | Higher is better | 1 (1.1%) | 1 | 1 (1.1%) | 1 | 0.316 [0.316, 0.316] | 0.316 | 0.316 |
|  | **Contour PPV** | Higher is better | 1 (1.1%) | 1 | 1 (1.1%) | 1 | 0.376 [0.376, 0.376] | 0.376 | 0.376 |
|  | **Contour TPR** | Higher is better | 1 (1.1%) | 1 | 1 (1.1%) | 1 | 0.665 [0.665, 0.665] | 0.665 | 0.665 |
|  | **K-means correctly clustered** | Higher is better | 1 (1.1%) | 1 | 1 (1.1%) | 1 | 92.0 [92.0, 92.0] | 92.0 | 92.0 |
|  | **K-means discarded** | Lower is better | 1 (1.1%) | 1 | 1 (1.1%) | 1 | 8.00 [8.00, 8.00] | 8.00 | 8.00 |
|  | **Minimum Euclidean distance** | Unclear / target-dependent | 1 (1.1%) | 1 | 1 (1.1%) | 1 | 6.15 [6.15, 6.15] | 6.15 | 6.15 |
|  | **SD** | Unclear / target-dependent | 1 (1.1%) | 2 | 0 (0.0%) | 0 | - | - | - |
|  | **t-SNE mean distance** | Unclear / target-dependent | 1 (1.1%) | 2 | 0 (0.0%) | 0 | - | - | - |
|  | **Automated structure-detection success rate** | Higher is better | 0 (0.0%) | 0 | 1 (1.1%) | 1 | 90.0 [90.0, 90.0] | 90.0 | 90.0 |
| **Perceptual similarity** | **LPIPS** | Lower is better | 8 (8.6%) | 17 | 8 (8.6%) | 17 | 0.309 [0.020, 0.429] | 0.005 | 0.940 |
| **Pixel/structural similarity** | **SSIM** | Higher is better | 25 (26.9%) | 48 | 25 (26.9%) | 48 | 0.765 [0.600, 0.987] | 0.106 | 88.0 |
|  | **MS-SSIM** | Higher is better | 9 (9.7%) | 17 | 9 (9.7%) | 17 | 0.400 [0.330, 0.701] | 0.110 | 0.970 |
|  | **3D-SSIM** | Higher is better | 1 (1.1%) | 1 | 1 (1.1%) | 1 | 0.975 [0.975, 0.975] | 0.975 | 0.975 |
| **Privacy/memorisation similarity** | **Euclidean distance in InceptionV3 feature space** | Lower is better | 1 (1.1%) | 1 | 0 (0.0%) | 0 | - | - | - |
| **Reconstruction error** | **PSNR** | Higher is better | 20 (21.5%) | 35 | 20 (21.5%) | 35 | 26.7 [19.8, 40.6] | 6.48 | 64.7 |
|  | **MSE** | Lower is better | 5 (5.4%) | 5 | 5 (5.4%) | 5 | 4.65 [0.210, 199.4] | 0.052 | 204.3 |
|  | **MAE** | Lower is better | 1 (1.1%) | 2 | 1 (1.1%) | 2 | 0.015 [0.008, 0.022] | 0.001 | 0.030 |
|  | **RMSE** | Lower is better | 1 (1.1%) | 1 | 1 (1.1%) | 1 | 0.081 [0.081, 0.081] | 0.081 | 0.081 |

## Figure S1. Study-level composition of primary downstream real-to-synthetic ratios.

Bars show the number of studies contributing at least one primary ratio within each downstream analytical strategy. Colors indicate the magnitude of the primary ratio, including fewer synthetic than real images, approximately 1:1 composition, more synthetic than real images, or synthetic-only composition. Internal labels show the number and percentage of studies within each strategy when a category includes more than five studies; labels outside the bars show the total number and percentage of studies in that strategy among all studies included in the figure. Studies could contribute to more than one analytical strategy.

## Figure S2. Downstream-relevant real-to-synthetic ratios by study and analytical use.

## Figure S3. Explainability and model-interpretability evidence.

## Table S14. Inter-rater reliability statistics before risk-of-bias consensus.

| Domain | Domain label | Kappa type | n | Kappa | Observed agreement | Expected agreement | Paired (all, n) | Paired (non-N/A, n) | Percent agreement (all) | Disagreements, n |
| --- | --- | --- | --- | --- | --- | --- | --- | --- | --- | --- |
| **D1** | **Sampling strategy** | Cohen kappa, unweighted | 159 | 0.124 | 0.635 | 0.584 | 159 | 159 | 63.5% | 58 |
|  |  | Weighted kappa, linear | 159 | 0.122 | 0.792 | 0.764 | 159 | 159 | 63.5% | 58 |
|  |  | Weighted kappa, quadratic | 159 | 0.119 | 0.871 | 0.854 | 159 | 159 | 63.5% | 58 |
| **D2** | **Source population description** | Cohen kappa, unweighted | 159 | 0.018 | 0.459 | 0.449 | 159 | 159 | 45.9% | 86 |
|  |  | Weighted kappa, linear | 159 | 0.020 | 0.708 | 0.702 | 159 | 159 | 45.9% | 86 |
|  |  | Weighted kappa, quadratic | 159 | 0.023 | 0.832 | 0.828 | 159 | 159 | 45.9% | 86 |
| **D3** | **Image quality and preprocessing control** | Cohen kappa, unweighted | 158 | -0.107 | 0.411 | 0.468 | 158 | 158 | 41.1% | 93 |
|  |  | Weighted kappa, linear | 158 | -0.070 | 0.652 | 0.675 | 158 | 158 | 41.1% | 93 |
|  |  | Weighted kappa, quadratic | 158 | -0.026 | 0.772 | 0.778 | 158 | 158 | 41.1% | 93 |
| **D4** | **Generative architecture reporting** | Cohen kappa, unweighted | 159 | -0.043 | 0.730 | 0.741 | 159 | 159 | 73.0% | 43 |
|  |  | Weighted kappa, linear | 159 | -0.027 | 0.858 | 0.862 | 159 | 159 | 73.0% | 43 |
|  |  | Weighted kappa, quadratic | 159 | -0.001 | 0.923 | 0.923 | 159 | 159 | 73.0% | 43 |
| **D5** | **Generation-validation data separation** | Cohen kappa, unweighted | 159 | 0.116 | 0.516 | 0.452 | 159 | 159 | 51.6% | 77 |
|  |  | Weighted kappa, linear | 159 | 0.126 | 0.720 | 0.680 | 159 | 159 | 51.6% | 77 |
|  |  | Weighted kappa, quadratic | 159 | 0.140 | 0.822 | 0.793 | 159 | 159 | 51.6% | 77 |
| **D6** | **Outcome definition and reporting** | Cohen kappa, unweighted | 159 | -0.065 | 0.679 | 0.699 | 159 | 159 | 67.9% | 51 |
|  |  | Weighted kappa, linear | 159 | -0.074 | 0.830 | 0.842 | 159 | 159 | 67.9% | 51 |
|  |  | Weighted kappa, quadratic | 159 | -0.088 | 0.906 | 0.913 | 159 | 159 | 67.9% | 51 |
| **D7** | **Data completeness and statistical reporting** | Cohen kappa, unweighted | 159 | 0.072 | 0.428 | 0.383 | 159 | 159 | 42.8% | 91 |
|  |  | Weighted kappa, linear | 159 | 0.094 | 0.651 | 0.615 | 159 | 159 | 42.8% | 91 |
|  |  | Weighted kappa, quadratic | 159 | 0.119 | 0.763 | 0.730 | 159 | 159 | 42.8% | 91 |
| **Pooled D1-D7** |  | Cohen kappa, unweighted | 1112 | 0.172 | 0.551 | 0.458 | 1112 | 1112 | 55.1% | 499 |
|  |  | Weighted kappa, linear | 1112 | 0.242 | 0.745 | 0.663 | 1112 | 1112 | 55.1% | 499 |
|  |  | Weighted kappa, quadratic | 1112 | 0.322 | 0.841 | 0.766 | 1112 | 1112 | 55.1% | 499 |

## Figure S4. Final study-level risk-of-bias assessment across included studies.

## Figure S5. Reproducibility and openness indicators across included studies.

Bars show the distribution of reporting or availability status for key reproducibility and openness dimensions, including code and data availability, generative-model artefacts, training reproducibility details, evaluation protocols, and independent/community validation.

## Figure S6. Most frequently used public or community datasets across included studies.

Bar plot showing the most frequently reported canonical datasets, grouped from reproducibility metadata and summarized by dataset provenance and benchmark use.

# References

1.	Borji A. Pros and cons of GAN evaluation measures. Computer Vision and Image Understanding Academic Press Inc.; 2019 Feb 9;179:41–65. doi: 10.1016/j.cviu.2018.10.009

2.	Naeem MF, Oh SJ, Uh Y, Choi Y, Yoo J. Reliable Fidelity and Diversity Metrics for Generative Models. 37th International Conference on Machine Learning, ICML 2020 International Machine Learning Society (IMLS); 2020 Feb 23;PartF168147-10:7133–7142.

3.	Borji A. Pros and cons of GAN evaluation measures: New developments. Computer Vision and Image Understanding Academic Press; 2022 Jan 1;215:103329. doi: 10.1016/j.cviu.2021.103329

4.	Alaa A, Van Breugel B, Saveliev ES, van der Schaar M. How Faithful is your Synthetic Data? {S}ample-level Metrics for Evaluating and Auditing Generative Models. In: Chaudhuri K, Jegelka S, Song L, Szepesvari C, Niu G, Sabato S, editors. Proceedings of the 39th International Conference on Machine Learning PMLR; 2022. p. 290–306. Available from: https://proceedings.mlr.press/v162/alaa22a.html

5.	Betzalel E, Penso C, Fetaya E. Evaluation Metrics for Generative Models: An Empirical Study. Machine Learning and Knowledge Extraction Multidisciplinary Digital Publishing Institute; 2024 July 7;6(3):1531–1544. doi: 10.3390/make6030073

6.	Skandarani Y, Jodoin PM, Lalande A. GANs for Medical Image Synthesis: An Empirical Study. Journal of Imaging MDPI; 2023 Mar 1;9(3):69. doi: 10.3390/JIMAGING9030069/S1

7.	Schaudt D, Späte C, von Schwerin R, Reichert M, von Schwerin M, Beer M, Kloth C. A Critical Assessment of Generative Models for Synthetic Data Augmentation on Limited Pneumonia X-ray Data. Bioengineering Multidisciplinary Digital Publishing Institute (MDPI); 2023 Dec 1;10(12):1421. PMID:38136012

8.	Deo Y, Jia Y, Lassila T, Smith WAP, Lawton T, Kang S, Frangi AF, Habli I. Metrics that matter: Evaluating image quality metrics for medical image generation. 2025 May 12; doi: 10.48550/ARXIV.2505.07175

9.	Dohmen M, Klemens MA, Baltruschat IM, Truong T, Lenga M. Similarity and quality metrics for MR image-to-image translation. Scientific Reports 2025 15:1 Nature Publishing Group; 2025 Jan 31;15(1):3853-. PMID:39890963

10.	Bargsten L, Schlaefer A. SpeckleGAN: a generative adversarial network with an adaptive speckle layer to augment limited training data for ultrasound image processing. Int J CARS 2020 Sept;15(9):1427–1436. doi: 10.1007/s11548-020-02203-1

11.	Cha KH, Petrick N, Pezeshk A, Graff CG, Sharma D, Badal A, Sahiner B. Evaluation of data augmentation via synthetic images for improved breast mass detection on mammograms using deep learning. J Med Imag 2019 Nov 22;7(01):1. doi: 10.1117/1.JMI.7.1.012703

12.	Chedid N, Sadda P, Gonchigar A, Langdon J, Porrino J, Haims A, Taylor RA. Synthesis of fracture radiographs with deep neural networks. Health Inf Sci Syst 2020 Dec;8(1):21. doi: 10.1007/s13755-020-00111-x

13.	Cronin NJ, Finni T, Seynnes O. Using deep learning to generate synthetic B-mode musculoskeletal ultrasound images. Computer Methods and Programs in Biomedicine 2020 Nov;196:105583. doi: 10.1016/j.cmpb.2020.105583

14.	Diller G-P, Vahle J, Radke R, Vidal MLB, Fischer AJ, Bauer UMM, Sarikouch S, Berger F, Beerbaum P, Baumgartner H, Orwat S, for the German Competence Network for Congenital Heart Defects Investigators. Utility of deep learning networks for the generation of artificial cardiac magnetic resonance images in congenital heart disease. BMC Med Imaging 2020 Dec;20(1):113. doi: 10.1186/s12880-020-00511-1

15.	He X, Fang L, Rabbani H, Chen X, Liu Z. Retinal optical coherence tomography image classification with label smoothing generative adversarial network. Neurocomputing 2020 Sept;405:37–47. doi: 10.1016/j.neucom.2020.04.044

16.	Islam J, Zhang Y. GAN-based synthetic brain PET image generation. Brain Inf 2020 Dec;7(1):3. doi: 10.1186/s40708-020-00104-2

17.	Miyoshi T, Higaki A, Kawakami H, Yamaguchi O. Automated interpretation of the coronary angioscopy with deep convolutional neural networks. Open Heart 2020 May;7(1):e001177. doi: 10.1136/openhrt-2019-001177

18.	Nishio M, Muramatsu C, Noguchi S, Nakai H, Fujimoto K, Sakamoto R, Fujita H. Attribute-guided image generation of three-dimensional computed tomography images of lung nodules using a generative adversarial network. Computers in Biology and Medicine 2020 Nov;126:104032. doi: 10.1016/j.compbiomed.2020.104032

19.	Qin Z, Liu Z, Zhu P, Xue Y. A GAN-based image synthesis method for skin lesion classification. Computer Methods and Programs in Biomedicine 2020 Oct;195:105568. doi: 10.1016/j.cmpb.2020.105568

20.	Shi G, Wang J, Qiang Y, Yang X, Zhao J, Hao R, Yang W, Du Q, Kazihise NGF. Knowledge-guided synthetic medical image adversarial augmentation for ultrasonography thyroid nodule classification. Computer Methods and Programs in Biomedicine Elsevier; 2020 Nov 1;196:105611. PMID:32650266

21.	Waheed A, Goyal M, Gupta D, Khanna A, Al-Turjman F, Pinheiro PR. CovidGAN: Data Augmentation Using Auxiliary Classifier GAN for Improved Covid-19 Detection. IEEE Access 2020;8:91916–91923. doi: 10.1109/ACCESS.2020.2994762

22.	Wang Y, Zhou L, Wang M, Shao C, Shi L, Yang S, Zhang Z, Feng M, Shan F, Liu L. Combination of generative adversarial network and convolutional neural network for automatic subcentimeter pulmonary adenocarcinoma classification. Quant Imaging Med Surg 2020 June;10(6):1249–1264. doi: 10.21037/qims-19-982

23.	Yoo TK, Choi JY, Jang Y, Oh E, Ryu IH. Toward automated severe pharyngitis detection with smartphone camera using deep learning networks. Computers in Biology and Medicine 2020 Oct;125:103980. doi: 10.1016/j.compbiomed.2020.103980

24.	Yu H, Zhang X. Synthesis of Prostate MR Images for Classification Using Capsule Network-Based GAN Model. Sensors 2020 Oct 9;20(20):5736. doi: 10.3390/s20205736

25.	Zheng C, Xie X, Zhou K, Chen B, Chen J, Ye H, Li W, Qiao T, Gao S, Yang J, Liu J. Assessment of Generative Adversarial Networks Model for Synthetic Optical Coherence Tomography Images of Retinal Disorders. Trans Vis Sci Tech 2020 May 27;9(2):29. doi: 10.1167/tvst.9.2.29

26.	De Souza LA, Passos LA, Mendel R, Ebigbo A, Probst A, Messmann H, Palm C, Papa JP. Assisting Barrett’s esophagus identification using endoscopic data augmentation based on Generative Adversarial Networks. Computers in Biology and Medicine 2020 Nov;126:104029. doi: 10.1016/j.compbiomed.2020.104029

27.	Acar E, Şahin E, Yılmaz İ. Improving effectiveness of different deep learning-based models for detecting COVID-19 from computed tomography (CT) images. Neural computing & applications Neural Comput Appl; 2021 Dec;33(24):17589–17609. PMID:34345118

28.	Ahmad B, Jun S, Palade V, You Q, Mao L, Zhongjie M. Improving Skin Cancer Classification Using Heavy-Tailed Student T-Distribution in Generative Adversarial Networks (TED-GAN). Diagnostics 2021 Nov 19;11(11):2147. doi: 10.3390/diagnostics11112147

29.	Durán-Rosal AM, Camacho-Cañamón J, Gutiérrez PA, Guiote Moreno MV, Rodríguez-Cáceres E, Vallejo Casas JA, Hervás-Martínez C. Ordinal classification of the affectation level of 3D-images in Parkinson diseases. Sci Rep 2021 Mar 29;11(1):7067. doi: 10.1038/s41598-021-86538-y

30.	Gao Y, Ghodrati V, Kalbasi A, Fu J, Ruan D, Cao M, Wang C, Eilber FC, Bernthal N, Bukata S, Dry SM, Nelson SD, Kamrava M, Lewis J, Low DA, Steinberg M, Hu P, Yang Y. Prediction of soft tissue sarcoma response to radiotherapy using longitudinal diffusion MRI and a deep neural network with generative adversarial network‐based data augmentation. Medical Physics 2021 June;48(6):3262–3372. doi: 10.1002/mp.14897

31.	Gómez S, Romo-Bucheli D, Martínez F. A digital cardiac disease biomarker from a generative progressive cardiac cine-MRI representation. Biomedical engineering letters Biomed Eng Lett; 2021 Feb;12(1):75–84. PMID:35186361

32.	He F, Chen S, Li S, Zhou L, Zhang H, Peng H, Huang X. Colonoscopic Image Synthesis For Polyp Detector Enhancement Via Gan And Adversarial Training. 2021 IEEE 18th International Symposium on Biomedical Imaging (ISBI) Nice, France: IEEE; 2021. p. 1887–1891. doi: 10.1109/ISBI48211.2021.9434050

33.	Jiang Y, Chen H, Loew M, Ko H. COVID-19 CT Image Synthesis With a Conditional Generative Adversarial Network. IEEE J Biomed Health Inform 2021 Feb;25(2):441–452. doi: 10.1109/JBHI.2020.3042523

34.	Karakanis S, Leontidis G. Lightweight deep learning models for detecting COVID-19 from chest X-ray images. Computers in Biology and Medicine 2021 Mar;130:104181. doi: 10.1016/j.compbiomed.2020.104181

35.	Karbhari Y, Basu A, Geem ZW, Han G-T, Sarkar R. Generation of Synthetic Chest X-ray Images and Detection of COVID-19: A Deep Learning Based Approach. Diagnostics 2021 May 18;11(5):895. doi: 10.3390/diagnostics11050895

36.	Kim M, Kim S, Kim M, Bae H-J, Park J-W, Kim N. Realistic high-resolution lateral cephalometric radiography generated by progressive growing generative adversarial network and quality evaluations. Sci Rep 2021 June 15;11(1):12563. doi: 10.1038/s41598-021-91965-y

37.	Kossen T, Subramaniam P, Madai VI, Hennemuth A, Hildebrand K, Hilbert A, Sobesky J, Livne M, Galinovic I, Khalil AA, Fiebach JB, Frey D. Synthesizing anonymized and labeled TOF-MRA patches for brain vessel segmentation using generative adversarial networks. Computers in Biology and Medicine 2021 Apr;131:104254. doi: 10.1016/j.compbiomed.2021.104254

38.	Liu Y, Meng L, Zhong J. MAGAN: Mask Attention Generative Adversarial Network for Liver Tumor CT Image Synthesis. Peng J, editor. Journal of Healthcare Engineering 2021 Jan 30;2021:1–11. doi: 10.1155/2021/6675259

39.	Montero A, Bonet-Carne E, Burgos-Artizzu XP. Generative Adversarial Networks to Improve Fetal Brain Fine-Grained Plane Classification. Sensors 2021 Nov 29;21(23):7975. doi: 10.3390/s21237975

40.	Morís DI, De Moura Ramos JJ, Buján JN, Hortas MO. Data augmentation approaches using cycle-consistent adversarial networks for improving COVID-19 screening in portable chest X-ray images. Expert Systems with Applications 2021 Dec;185:115681. doi: 10.1016/j.eswa.2021.115681

41.	Mutepfe F, Kalejahi BK, Meshgini S, Danishvar S. Generative Adversarial Network Image Synthesis Method for Skin Lesion Generation and Classification. Journal of Medical Signals & Sensors 2021 Oct;11(4):237–252. doi: 10.4103/jmss.JMSS_53_20

42.	Park JE, Eun D, Kim HS, Lee DH, Jang RW, Kim N. Generative adversarial network for glioblastoma ensures morphologic variations and improves diagnostic model for isocitrate dehydrogenase mutant type. Sci Rep 2021 May 10;11(1):9912. doi: 10.1038/s41598-021-89477-w

43.	Rangarajan AK, Ramachandran HK. A preliminary analysis of AI based smartphone application for diagnosis of COVID-19 using chest X-ray images. Expert Systems with Applications 2021 Nov;183:115401. doi: 10.1016/j.eswa.2021.115401

44.	Sajjad M, Ramzan F, Khan MUG, Rehman A, Kolivand M, Fati SM, Bahaj SA. Deep convolutional generative adversarial network for Alzheimer’s disease classification using positron emission tomography ( PET ) and synthetic data augmentation. Microscopy Res & Technique 2021 Dec;84(12):3023–3034. doi: 10.1002/jemt.23861

45.	Segal B, Rubin DM, Rubin G, Pantanowitz A. Evaluating the Clinical Realism of Synthetic Chest X-Rays Generated Using Progressively Growing GANs. SN COMPUT SCI 2021 July;2(4):321. doi: 10.1007/s42979-021-00720-7

46.	Shen T, Hao K, Gou C, Wang F-Y. Mass Image Synthesis in Mammogram with Contextual Information Based on GANs. Computer Methods and Programs in Biomedicine 2021 Apr;202:106019. doi: 10.1016/j.cmpb.2021.106019

47.	Toda R, Teramoto A, Tsujimoto M, Toyama H, Imaizumi K, Saito K, Fujita H. Synthetic CT image generation of shape-controlled lung cancer using semi-conditional InfoGAN and its applicability for type classification. International journal of computer assisted radiology and surgery Int J Comput Assist Radiol Surg; 2021 Feb;16(2):241–251. PMID:33428062

48.	V J S, D JF. Deep Learning Algorithm for COVID-19 Classification Using Chest X-Ray Images. Moraru L, editor. Computational and Mathematical Methods in Medicine 2021 Nov 9;2021:1–10. doi: 10.1155/2021/9269173

49.	Wang Q, Zhang X, Zhang W, Gao M, Huang S, Wang J, Zhang J, Yang D, Liu C. Realistic Lung Nodule Synthesis With Multi-Target Co-Guided Adversarial Mechanism. IEEE transactions on medical imaging IEEE Trans Med Imaging; 2021 Sept;40(9):2343–2353. PMID:33939610

50.	Zheng C, Bian F, Li L, Xie X, Liu H, Liang J, Chen X, Wang Z, Qiao T, Yang J, Zhang M. Assessment of Generative Adversarial Networks for Synthetic Anterior Segment Optical Coherence Tomography Images in Closed-Angle Detection. Trans Vis Sci Tech 2021 Apr 30;10(4):34. doi: 10.1167/tvst.10.4.34

51.	Albahli S, Ahmad Hassan Yar GN. AI-driven deep convolutional neural networks for chest X-ray pathology identification. XST 2022 Mar 15;30(2):365–376. doi: 10.3233/XST-211082

52.	Alrashedy HHN, Almansour AF, Ibrahim DM, Hammoudeh MAA. BrainGAN: Brain MRI Image Generation and Classification Framework Using GAN Architectures and CNN Models. Sensors 2022 June 6;22(11):4297. doi: 10.3390/s22114297

53.	Amin J, Anjum MA, Sharif M, Kadry S, Nadeem A, Ahmad SF. Liver Tumor Localization Based on YOLOv3 and 3D-Semantic Segmentation Using Deep Neural Networks. Diagnostics 2022 Mar 27;12(4):823. doi: 10.3390/diagnostics12040823

54.	Chai L, Wang Z, Chen J, Zhang G, Alsaadi FE, Alsaadi FE, Liu Q. Synthetic augmentation for semantic segmentation of class imbalanced biomedical images: A data pair generative adversarial network approach. Computers in Biology and Medicine 2022 Nov;150:105985. doi: 10.1016/j.compbiomed.2022.105985

55.	Coyner AS, Chen JS, Chang K, Singh P, Ostmo S, Chan RVP, Chiang MF, Kalpathy-Cramer J, Campbell JP. Synthetic Medical Images for Robust, Privacy-Preserving Training of Artificial Intelligence. Ophthalmology Science 2022 June;2(2):100126. doi: 10.1016/j.xops.2022.100126

56.	Gan M, Wang C. Esophageal optical coherence tomography image synthesis using an adversarially learned variational autoencoder. Biomed Opt Express 2022 Mar 1;13(3):1188. doi: 10.1364/BOE.449796

57.	Gheorghiță BA, Itu LM, Sharma P, Suciu C, Wetzl J, Geppert C, Ali MAA, Lee AM, Piechnik SK, Neubauer S, Petersen SE, Schulz-Menger J, Chițiboi T. Improving robustness of automatic cardiac function quantification from cine magnetic resonance imaging using synthetic image data. Sci Rep 2022 Feb 14;12(1):2391. doi: 10.1038/s41598-022-06315-3

58.	Guan Q, Chen Y, Wei Z, Heidari AA, Hu H, Yang X-H, Zheng J, Zhou Q, Chen H, Chen F. Medical image augmentation for lesion detection using a texture-constrained multichannel progressive GAN. Computers in Biology and Medicine 2022 June;145:105444. doi: 10.1016/j.compbiomed.2022.105444

59.	Guo Z-Z, Zheng L-X, Huang D-T, Yan T, Su Q-L. RS-FFGAN:Generative adversarial network based on real sample feature fusion for pediatric CXR image data enhancement. Journal of Radiation Research and Applied Sciences 2022 Dec;15(4):100461. doi: 10.1016/j.jrras.2022.100461

60.	Heenaye-Mamode Khan M, Gooda Sahib-Kaudeer N, Dayalen M, Mahomedaly F, Sinha GR, Nagwanshi KK, Taylor A. Multi-Class Skin Problem Classification Using Deep Generative Adversarial Network (DGAN). Loddo A, editor. Computational Intelligence and Neuroscience 2022 Mar 24;2022:1–13. doi: 10.1155/2022/1797471

61.	Hussain BZ, Andleeb I, Ansari MS, Joshi AM, Kanwal N. Wasserstein GAN based Chest X-Ray Dataset Augmentation for Deep Learning Models: COVID-19 Detection Use-Case. 2022 44th Annual International Conference of the IEEE Engineering in Medicine & Biology Society (EMBC) Glasgow, Scotland, United Kingdom: IEEE; 2022. p. 2058–2061. doi: 10.1109/EMBC48229.2022.9871519

62.	Kim M, Kim YN, Jang M, Hwang J, Kim H-K, Yoon SC, Kim YJ, Kim N. Synthesizing realistic high-resolution retina image by style-based generative adversarial network and its utilization. Sci Rep 2022 Oct 15;12(1):17307. doi: 10.1038/s41598-022-20698-3

63.	Liang J, Yang X, Huang Y, Li H, He S, Hu X, Chen Z, Xue W, Cheng J, Ni D. Sketch guided and progressive growing GAN for realistic and editable ultrasound image synthesis. Medical Image Analysis 2022 July;79:102461. doi: 10.1016/j.media.2022.102461

64.	Mukherkjee D, Saha P, Kaplun D, Sinitca A, Sarkar R. Brain tumor image generation using an aggregation of GAN models with style transfer. Sci Rep 2022 June 1;12(1):9141. doi: 10.1038/s41598-022-12646-y

65.	Ovalle-Magallanes E, Avina-Cervantes JG, Cruz-Aceves I, Ruiz-Pinales J. Improving convolutional neural network learning based on a hierarchical bezier generative model for stenosis detection in X-ray images. Computer Methods and Programs in Biomedicine 2022 June;219:106767. doi: 10.1016/j.cmpb.2022.106767

66.	Qiang N, Dong Q, Liang H, Li J, Zhang S, Zhang C, Ge B, Sun Y, Gao J, Liu T, Yue H, Zhao S. Learning brain representation using recurrent Wasserstein generative adversarial net. Computer Methods and Programs in Biomedicine 2022 Aug;223:106979. doi: 10.1016/j.cmpb.2022.106979

67.	Qin X, Bui FM, Nguyen HH, Han Z. Learning From Limited and Imbalanced Medical Images With Finer Synthetic Images From GANs. IEEE Access 2022;10:91663–91677. doi: 10.1109/ACCESS.2022.3202560

68.	Ramirez S, Brieu M, Forouzesh N. Low Data Image Analysis with a Generative Adversarial Network: A Case Study on Women Pelvic MRI Scans. 2022 IEEE International Conference on Bioinformatics and Biomedicine (BIBM) Las Vegas, NV, USA: IEEE; 2022. p. 2690–2695. doi: 10.1109/BIBM55620.2022.9995445

69.	Shah PM, Ullah H, Ullah R, Shah D, Wang Y, Islam SU, Gani A, Rodrigues JJPC. DC‐GAN ‐based synthetic X‐ray images augmentation for increasing the performance of EfficientNet for COVID ‐19 detection. Expert Systems 2022 Mar;39(3):e12823. doi: 10.1111/exsy.12823

70.	Sreejith Kumar AJ, Chong RS, Crowston JG, Chua J, Bujor I, Husain R, Vithana EN, Girard MJA, Ting DSW, Cheng C-Y, Aung T, Popa-Cherecheanu A, Schmetterer L, Wong D. Evaluation of Generative Adversarial Networks for High-Resolution Synthetic Image Generation of Circumpapillary Optical Coherence Tomography Images for Glaucoma. JAMA Ophthalmol 2022 Oct 1;140(10):974. doi: 10.1001/jamaophthalmol.2022.3375

71.	Sun L, Chen J, Xu Y, Gong M, Yu K, Batmanghelich K. Hierarchical Amortized GAN for 3D High Resolution Medical Image Synthesis. IEEE J Biomed Health Inform 2022 Aug;26(8):3966–3975. doi: 10.1109/JBHI.2022.3172976

72.	Thambawita V, Salehi P, Sheshkal SA, Hicks SA, Hammer HL, Parasa S, Lange TD, Halvorsen P, Riegler MA. SinGAN-Seg: Synthetic training data generation for medical image segmentation. Stoean R, editor. PLoS ONE 2022 May 2;17(5):e0267976. doi: 10.1371/journal.pone.0267976

73.	Veturi YA, Woof W, Lazebnik T, Moghul I, Woodward-Court P, Wagner SK, Cabral De Guimarães TA, Daich Varela M, Liefers B, Patel PJ, Beck S, Webster AR, Mahroo O, Keane PA, Michaelides M, Balaskas K, Pontikos N. SynthEye: Investigating the Impact of Synthetic Data on Artificial Intelligence-assisted Gene Diagnosis of Inherited Retinal Disease. Ophthalmology Science 2023 June;3(2):100258. doi: 10.1016/j.xops.2022.100258

74.	Yoon D, Kong H-J, Kim BS, Cho WS, Lee JC, Cho M, Lim MH, Yang SY, Lim SH, Lee J, Song JH, Chung GE, Choi JM, Kang HY, Bae JH, Kim S. Colonoscopic image synthesis with generative adversarial network for enhanced detection of sessile serrated lesions using convolutional neural network. Sci Rep 2022 Jan 7;12(1):261. doi: 10.1038/s41598-021-04247-y

75.	Zhao M, Lu Z, Zhu S, Wang X, Feng J. Automatic generation of retinal optical coherence tomography images based on generative adversarial networks. Medical Physics 2022 Nov;49(11):7357–7367. doi: 10.1002/mp.15988

76.	Bajhaiya D, Unni SN, Koushik AK. Deep learning–powered generation of artificial endoscopic images of GI tract ulcers. iGIE 2023 Dec;2(4):452-463.e2. doi: 10.1016/j.igie.2023.08.002

77.	Behara K, Bhero E, Agee JT. Skin Lesion Synthesis and Classification Using an Improved DCGAN Classifier. Diagnostics 2023 Aug 9;13(16):2635. doi: 10.3390/diagnostics13162635

78.	Buoso S, Joyce T, Schulthess N, Kozerke S. MRXCAT2.0: Synthesis of realistic numerical phantoms by combining left-ventricular shape learning, biophysical simulations and tissue texture generation. Journal of Cardiovascular Magnetic Resonance 2023 Feb;25(1):25. doi: 10.1186/s12968-023-00934-z

79.	Dash A, Swarnkar T. CoVaD-GAN:An efficient Data Augmentation technique for COVID CXR Image Classification. 2023 2nd International Conference on Ambient Intelligence in Health Care (ICAIHC) Bhubaneswar, India: IEEE; 2023. p. 1–7. doi: 10.1109/ICAIHC59020.2023.10431455

80.	Guo K, Chen J, Qiu T, Guo S, Luo T, Chen T, Ren S. MedGAN: An adaptive GAN approach for medical image generation. Computers in Biology and Medicine 2023 Sept;163:107119. doi: 10.1016/j.compbiomed.2023.107119

81.	Hardy R, Klepich J, Mitchell R, Hall S, Villareal J, Ilin C. Improving nonalcoholic fatty liver disease classification performance with latent diffusion models. Sci Rep 2023 Dec 7;13(1):21619. doi: 10.1038/s41598-023-48062-z

82.	Hou Y, Navarro-Cía M. A computationally-inexpensive strategy in CT image data augmentation for robust deep learning classification in the early stages of an outbreak. Biomed Phys Eng Express 2023 Sept 1;9(5):055003. doi: 10.1088/2057-1976/ace4cf

83.	Jang M, Bae H, Kim M, Park SY, Son A, Choi SJ, Choe J, Choi HY, Hwang HJ, Noh HN, Seo JB, Lee SM, Kim N. Image Turing test and its applications on synthetic chest radiographs by using the progressive growing generative adversarial network. Sci Rep 2023 Feb 9;13(1):2356. doi: 10.1038/s41598-023-28175-1

84.	Katakis S, Barotsis N, Kakotaritis A, Tsiganos P, Economou G, Panagiotopoulos E, Panayiotakis G. Generation of Musculoskeletal Ultrasound Images with Diffusion Models. BioMedInformatics 2023 May 23;3(2):405–421. doi: 10.3390/biomedinformatics3020027

85.	Khader F, Müller-Franzes G, Tayebi Arasteh S, Han T, Haarburger C, Schulze-Hagen M, Schad P, Engelhardt S, Baeßler B, Foersch S, Stegmaier J, Kuhl C, Nebelung S, Kather JN, Truhn D. Denoising diffusion probabilistic models for 3D medical image generation. Sci Rep 2023 May 5;13(1):7303. doi: 10.1038/s41598-023-34341-2

86.	Khosravi B, Rouzrokh P, Mickley JP, Faghani S, Larson AN, Garner HW, Howe BM, Erickson BJ, Taunton MJ, Wyles CC. Creating High Fidelity Synthetic Pelvis Radiographs Using Generative Adversarial Networks: Unlocking the Potential of Deep Learning Models Without Patient Privacy Concerns. The Journal of Arthroplasty 2023 Oct;38(10):2037-2043.e1. doi: 10.1016/j.arth.2022.12.013

87.	Lyu F, Ye M, Carlsen JF, Erleben K, Darkner S, Yuen PC. Pseudo-Label Guided Image Synthesis for Semi-Supervised COVID-19 Pneumonia Infection Segmentation. IEEE Trans Med Imaging 2023 Mar;42(3):797–809. doi: 10.1109/TMI.2022.3217501

88.	Malihi L, Hübner U, Richter ML, Moelleken M, Przysucha M, Busch D, Heggemann J, Hafer G, Wiemeyer S, Heidemann G, Dissemond J, Erfurt-Berge C, Barkhau C, Hendriks A, Hüsers J. Can Synthetic Images Improve CNN Performance in Wound Image Classification? In: Hägglund M, Blusi M, Bonacina S, Nilsson L, Cort Madsen I, Pelayo S, Moen A, Benis A, Lindsköld L, Gallos P, editors. Studies in Health Technology and Informatics IOS Press; 2023. doi: 10.3233/SHTI230311ISBN:978-1-64368-388-1

89.	Menon S, Mangalagiri J, Galita J, Morris M, Saboury B, Yesha Y, Yesha Y, Nguyen P, Gangopadhyay A, Chapman D. CCS-GAN: COVID-19 CT Scan Generation and Classification with Very Few Positive Training Images. J Digit Imaging 2023 Apr 17;36(4):1376–1389. doi: 10.1007/s10278-023-00811-2

90.	Naga Srinivasu P, Krishna TB, Ahmed S, Almusallam N, Khaled Alarfaj F, Allheeib N. Variational Autoencoders‐BasedSelf‐Learning Model for Tumor Identification and Impact Analysis from 2‐D MRI Images. Dogra A, editor. Journal of Healthcare Engineering 2023 Jan;2023(1):1566123. doi: 10.1155/2023/1566123

91.	Naz H, Nijhawan R, Ahuja NJ, Al-Otaibi S, Saba T, Bahaj SA, Rehman A. Ensembled Deep Convolutional Generative Adversarial Network for Grading Imbalanced Diabetic Retinopathy Recognition. IEEE Access 2023;11:120554–120568. doi: 10.1109/ACCESS.2023.3327900

92.	Nithya TM, Rajesh Kanna P, Vanithamani S, Santhi P. An Efficient PM - Multisampling Image Filtering with Enhanced CNN Architecture for Pneumonia Classfication. Biomedical Signal Processing and Control 2023 Sept;86:105296. doi: 10.1016/j.bspc.2023.105296

93.	Niu L, Chen X, Guo S. Bézier Curves Meet Deep Learning: A Novel Pretraining Method for Improved and Generalized Retinal Vessel Segmentation. 2023 IEEE International Conference on Bioinformatics and Biomedicine (BIBM) Istanbul, Turkiye: IEEE; 2023. p. 2159–2164. doi: 10.1109/BIBM58861.2023.10385674

94.	Packhäuser K, Folle L, Thamm F, Maier A. Generation of Anonymous Chest Radiographs Using Latent Diffusion Models for Training Thoracic Abnormality Classification Systems. 2023 IEEE 20th International Symposium on Biomedical Imaging (ISBI) Cartagena, Colombia: IEEE; 2023. p. 1–5. doi: 10.1109/ISBI53787.2023.10230346

95.	Rhyou S-Y, Yoo J-C. Aggregated micropatch-based deep learning neural network for ultrasonic diagnosis of cirrhosis. Artificial Intelligence in Medicine 2023 May;139:102541. doi: 10.1016/j.artmed.2023.102541

96.	Shavlokhova V, Vollmer A, Zouboulis CC, Vollmer M, Wollborn J, Lang G, Kübler A, Hartmann S, Stoll C, Roider E, Saravi B. Finetuning of GLIDE stable diffusion model for AI-based text-conditional image synthesis of dermoscopic images. Front Med 2023 Oct 20;10:1231436. doi: 10.3389/fmed.2023.1231436

97.	Shen Z, Ouyang X, Xiao B, Cheng J-Z, Shen D, Wang Q. Image synthesis with disentangled attributes for chest X-ray nodule augmentation and detection. Medical Image Analysis 2023 Feb;84:102708. doi: 10.1016/j.media.2022.102708

98.	Skandarani Y, Jodoin P-M, Lalande A. GANs for Medical Image Synthesis: An Empirical Study. J Imaging 2023 Mar 16;9(3):69. doi: 10.3390/jimaging9030069

99.	Tsai W-X, Li Y-C, Lin CH. Skin lesion classification based on multi-model ensemble with generated levels-of-detail images. Biomedical Signal Processing and Control 2023 Aug;85:105068. doi: 10.1016/j.bspc.2023.105068

100.	Xing X, Papanastasiou G, Walsh S, Yang G. Less Is More: Unsupervised Mask-Guided Annotated CT Image Synthesis With Minimum Manual Segmentations. IEEE Trans Med Imaging 2023 Sept;42(9):2566–2576. doi: 10.1109/TMI.2023.3260169

101.	Xiong Y, Luo Y, Tang Q, Zhang Y, Du B. Cervical OCT Image Synthesis via Noise Adaptation Diffusion Models. 2023 IEEE International Conference on Bioinformatics and Biomedicine (BIBM) Istanbul, Turkiye: IEEE; 2023. p. 2310–2313. doi: 10.1109/BIBM58861.2023.10385491

102.	Xu IRL, Van Booven DJ, Goberdhan S, Breto A, Porto J, Alhusseini M, Algohary A, Stoyanova R, Punnen S, Mahne A, Arora H. Generative Adversarial Networks Can Create High Quality Artificial Prostate Cancer Magnetic Resonance Images. JPM 2023 Mar 18;13(3):547. doi: 10.3390/jpm13030547

103.	Allan-Blitz L-T, Ambepitiya S, Prathapa J, Rietmeijer CA, Kularathne Y, Klausner JD. Synergistic pairing of synthetic image generation with disease classification modeling permits rapid digital classification tool development. Sci Rep 2024 Oct 27;14(1):25632. doi: 10.1038/s41598-024-77565-6

104.	Asadi F, Angsuwatanakul T, O’Reilly JA. Evaluating synthetic neuroimaging data augmentation for automatic brain tumour segmentation with a deep fully-convolutional network. IBRO Neuroscience Reports 2024 June;16:57–66. doi: 10.1016/j.ibneur.2023.12.002

105.	Assaf JF, Yazbeck H, Reinstein DZ, Archer TJ, Arbelaez J, Bteich Y, Arbelaez MC, Abou Mrad A, Awwad ST. Enhancing the Automated Detection of Implantable Collamer Lens Vault Using Generative Adversarial Networks and Synthetic Data on Optical Coherence Tomography. J Refract Surg 2024 Apr;40(4). doi: 10.3928/1081597X-20240214-01

106.	Balla B, Hibi A, Tyrrell PN. Diffusion-Based Image Synthesis or Traditional Augmentation for Enriching Musculoskeletal Ultrasound Datasets. BioMedInformatics 2024 Aug 29;4(3):1934–1948. doi: 10.3390/biomedinformatics4030106

107.	Bradbury R, Vallis KA, Papież BW. Paired Diffusion: Generation of Related, Synthetic PET-CT-Segmentation Scans Using Linked Denoising Diffusion Probabilistic Models. 2024 IEEE International Symposium on Biomedical Imaging (ISBI) Athens, Greece: IEEE; 2024. p. 1–5. doi: 10.1109/ISBI56570.2024.10635593

108.	Chaurasia AK, MacGregor S, Craig JE, Mackey DA, Hewitt AW. Assessing the Efficacy of Synthetic Optic Disc Images for Detecting Glaucomatous Optic Neuropathy Using Deep Learning. Trans Vis Sci Tech 2024 June 3;13(6):1. doi: 10.1167/tvst.13.6.1

109.	Deng B, Zheng X, Chen X, Zhang M. A Swin transformer encoder-based StyleGAN for unbalanced endoscopic image enhancement. Computers in Biology and Medicine 2024 June;175:108472. doi: 10.1016/j.compbiomed.2024.108472

110.	Diamantis DE, Gatoula P, Koulaouzidis A, Iakovidis DK. This Intestine Does Not Exist: Multiscale Residual Variational Autoencoder for Realistic Wireless Capsule Endoscopy Image Generation. IEEE Access 2024;12:25668–25683. doi: 10.1109/ACCESS.2024.3366801

111.	Gonzalez-Perez R, Lopez-Tiro F, Reyes-Amezcua I, Falcon-Morales E, Rodríguez-Guéant R-M, Hubert J, Daudon M, Ochoa-Ruiz G, Daul C. Evaluating the plausibility of synthetic images for improving automated endoscopic stone recognition. 2024 IEEE 37th International Symposium on Computer-Based Medical Systems (CBMS) Guadalajara, Mexico: IEEE; 2024. p. 126–133. doi: 10.1109/CBMS61543.2024.00029

112.	S G, B L. Deep Convolutional Generative Adversarial Network for Improved Cardiac Image Classification in Heart Disease Diagnosis. J Digit Imaging Inform med 2024 Dec 9;38(4):2146–2169. doi: 10.1007/s10278-024-01343-z

113.	Hossain MdR, Shakiruzzaman, Ferdous GJ, Hossain MdA. BrainACGAN: Auxiliary Classifier Generative Adversarial Network for Brain Tumor Images. 2024 International Conference on Innovations in Science, Engineering and Technology (ICISET) Chittagong, Bangladesh: IEEE; 2024. p. 1–6. doi: 10.1109/ICISET62123.2024.10939632

114.	Islam M, Zunair H, Mohammed N. CosSIF: Cosine similarity-based image filtering to overcome low inter-class variation in synthetic medical image datasets. Computers in Biology and Medicine 2024 Apr;172:108317. doi: 10.1016/j.compbiomed.2024.108317

115.	Kebaili A, Lapuyade-Lahorgue J, Vera P, Ruan S. Discriminative Hamiltonian variational autoencoder for accurate tumor segmentation in data-scarce regimes. Neurocomputing 2024 Nov;606:128360. doi: 10.1016/j.neucom.2024.128360

116.	Khosravi B, Li F, Dapamede T, Rouzrokh P, Gamble CU, Trivedi HM, Wyles CC, Sellergren AB, Purkayastha S, Erickson BJ, Gichoya JW. Synthetically enhanced: unveiling synthetic data’s potential in medical imaging research. eBioMedicine 2024 June;104:105174. doi: 10.1016/j.ebiom.2024.105174

117.	Kikuchi T, Hanaoka S, Nakao T, Takenaga T, Nomura Y, Mori H, Yoshikawa T. Synthesis of Hybrid Data Consisting of Chest Radiographs and Tabular Clinical Records Using Dual Generative Models for COVID-19 Positive Cases. J Digit Imaging Inform med 2024 Feb 13;37(3):1217–1227. doi: 10.1007/s10278-024-01015-y

118.	Kumar A, Singh RP. Application of Generative Adversarial Network for Data Augmentation in Diabetic Retinopathy. 2024 15th International Conference on Computing Communication and Networking Technologies (ICCCNT) Kamand, India: IEEE; 2024. p. 1–7. doi: 10.1109/ICCCNT61001.2024.10726154

119.	Moon HH, Jeong J, Park JE, Kim N, Choi C, Kim Y, Song SW, Hong C-K, Kim JH, Kim HS. Generative AI in glioma: Ensuring diversity in training image phenotypes to improve diagnostic performance for IDH mutation prediction. Neuro-Oncology 2024 June 3;26(6):1124–1135. doi: 10.1093/neuonc/noae012

120.	Oliveira GC, Rosa GH, Pedronette DCG, Papa JP, Kumar H, Passos LA, Kumar D. Robust deep learning for eye fundus images: Bridging real and synthetic data for enhancing generalization. Biomedical Signal Processing and Control 2024 Aug;94:106263. doi: 10.1016/j.bspc.2024.106263

121.	Park C, Kang J-W, Lee D-E, Son W, Lee S-M, Park C, Kim M. W-DRAG: A joint framework of WGAN with data random augmentation optimized for generative networks for bone marrow edema detection in dual energy CT. Computerized Medical Imaging and Graphics 2024 July;115:102387. doi: 10.1016/j.compmedimag.2024.102387

122.	Patil V, Arepally DR, Shah H. Performance Analysis of DCGAN and WGAN in Synthesizing Brain MRI Scans for Alzheimer’s Disease Detection Models. 2024 15th International Conference on Computing Communication and Networking Technologies (ICCCNT) Kamand, India: IEEE; 2024. p. 1–7. doi: 10.1109/ICCCNT61001.2024.10725987

123.	Patil A, Kour N, Vora P, Joshi AD, Ghotkar SN. Skin cancer classification using Progressive Growing of Generative Adversarial Network. 2024 International Conference on Emerging Technologies in Computer Science for Interdisciplinary Applications (ICETCS) Bengaluru, India: IEEE; 2024. p. 1–6. doi: 10.1109/ICETCS61022.2024.10544308

124.	Saihood A, Abdulhussien WR, Alzubaid L, Manoufali M, Gu Y. Fusion-driven semi-supervised learning-based lung nodules classification with dual-discriminator and dual-generator generative adversarial network. BMC Med Inform Decis Mak 2024 Dec 24;24(1):403. doi: 10.1186/s12911-024-02820-9

125.	Shakiruzzaman, Hossain MdR, Ferdous GJ, Hossain MdA. ChestACGAN: Auxiliary Classifier Generative Adversarial Network for Chest X-ray Images. 2024 International Conference on Innovations in Science, Engineering and Technology (ICISET) Chittagong, Bangladesh: IEEE; 2024. p. 1–6. doi: 10.1109/ICISET62123.2024.10941299

126.	Xu J, Hua Q, Jia X, Zheng Y, Hu Q, Bai B, Miao J, Zhu L, Zhang M, Tao R, Li Y, Luo T, Xie J, Zheng X, Gu P, Xing F, He C, Song Y, Dong Y, Xia S, Zhou J. Synthetic Breast Ultrasound Images: A Study to Overcome Medical Data Sharing Barriers. Research 2024 Jan;7:0532. doi: 10.34133/research.0532

127.	Xu Y, Liang J, Zhuo Y, Liu L, Xiao Y, Zhou L. TDASD: Generating medically significant fine-grained lung adenocarcinoma nodule CT images based on stable diffusion models with limited sample size. Computer Methods and Programs in Biomedicine 2024 May;248:108103. doi: 10.1016/j.cmpb.2024.108103

128.	Yalcin C, Abramova V, Terceño M, Oliver A, Silva Y, Lladó X. Hematoma expansion prediction in intracerebral hemorrhage patients by using synthesized CT images in an end-to-end deep learning framework. Computerized Medical Imaging and Graphics 2024 Oct;117:102430. doi: 10.1016/j.compmedimag.2024.102430

129.	Zhang C, Mei M, Mei Z, Wu B, Chen S, Lu M, Lu C. On efficient expanding training datasets of breast tumor ultrasound segmentation model. Computers in Biology and Medicine 2024 Dec;183:109274. doi: 10.1016/j.compbiomed.2024.109274

130.	Zheng Z, Wang M, Fan C, Wang C, He X, He X. Light&fast generative adversarial network for high-fidelity CT image synthesis of liver tumor. Computer Methods and Programs in Biomedicine 2024 Sept;254:108252. doi: 10.1016/j.cmpb.2024.108252

131.	Zhou J, Dai Y, Liu D, Zhu W, Xiang D, Chen X, Shi F, Xia W. Improving deep learning based segmentation of scars using multi-view images. Biomedical Signal Processing and Control 2024 Aug;94:106254. doi: 10.1016/j.bspc.2024.106254

132.	Dos Reis Carvalho A, Da Silva MV, Comin CH. Artificial vascular image generation using blood vessel texture maps. Computers in Biology and Medicine 2024 Dec;183:109226. doi: 10.1016/j.compbiomed.2024.109226

133.	A Hasib U, Md Abu R, Yang J, Bhatti UA, Ku CS, Por LY. YOLOv8 framework for COVID-19 and pneumonia detection using synthetic image augmentation. DIGITAL HEALTH 2025 May;11:20552076251341092. doi: 10.1177/20552076251341092

134.	Almuhaimeed A, Bilal A, Alzahrani A, Alrashidi M, Alghamdi M, Sarwar R. Brain tumor classification using GAN-augmented data with autoencoders and Swin Transformers. Front Med 2025 Aug 22;12:1635796. doi: 10.3389/fmed.2025.1635796

135.	Bharath HC, Pradeep N, Shashidhar R, Nanjappa Y. Synergistic medical genetic evolutionary optimization and deep convolutional generative augmentation with SHAP-driven interpretability for precise Alzheimer’s disease severity grading. Brain Inf 2025 Dec;12(1):31. doi: 10.1186/s40708-025-00280-z

136.	Brehmer A, Seibold C, Egger J, Majjouti K, Tapp-Herrenbrück M, Pinnekamp H, Priester V, Aleithe M, Fischer U, Hosters B, Kleesiek J. Fine-Grained Classification of Pressure Ulcers and Incontinence-Associated Dermatitis Using Multimodal Deep Learning: Algorithm Development and Validation Study. JMIR AI 2025 May 1;4:e67356–e67356. doi: 10.2196/67356

137.	Dahan E, Indelman HC, Perez-Agosto AM, Shiran C, Avinash G, Shaked D, Daniel N. CSG: A Context-Semantic Guided Diffusion Approach in De Novo Musculoskeletal Ultrasound Image Generation. 2025 47th Annual International Conference of the IEEE Engineering in Medicine and Biology Society (EMBC) Copenhagen, Denmark: IEEE; 2025. p. 1–7. doi: 10.1109/EMBC58623.2025.11252664

138.	Dai F, Yao S, Wang M, Zhu Y, Qiu X, Sun P, Qiu C, Yin J, Shen G, Sun J, Wang M, Wang Y, Yang Z, Sang J, Wang X, Sun F, Cai W, Zhang X, Lu H. Improving AI models for rare thyroid cancer subtype by text guided diffusion models. Nat Commun 2025 May 13;16(1):4449. doi: 10.1038/s41467-025-59478-8

139.	Ersarı B, Kola MG, Karaca EE, Işık FD, Kemer ÖE, Keçeli AS, Kaya A, Erdoğan TG, Uçan A. Denoising diffusion-based anterior segment optical coherence tomography (AS-OCT) image generation. Int Ophthalmol 2025 Oct 23;45(1):431. doi: 10.1007/s10792-025-03821-x

140.	Fukae J, Amasaki Y, Fujieda Y, Sone Y, Katagishi K, Horie T, Kamishima T, Atsumi T. Pre-trained convolutional neural network with transfer learning by artificial illustrated images classify power Doppler ultrasound images of rheumatoid arthritis joints. J Int Med Res 2025 Feb;53(2):03000605251318195. doi: 10.1177/03000605251318195

141.	Grabke EP, Taati B, Haider MA. Leveraging Clinical Text and Class Conditioning for 3D Prostate MRI Generation. IEEE Trans Biomed Eng 2025;1–10. doi: 10.1109/TBME.2025.3648426

142.	Guo B, Huang P, Wang Y, Guo Y. Prototype-Driven Class-Conditional Synthesis for High-Quality Chest X-ray Image Generation. 2025 47th Annual International Conference of the IEEE Engineering in Medicine and Biology Society (EMBC) Copenhagen, Denmark: IEEE; 2025. p. 1–5. doi: 10.1109/EMBC58623.2025.11254294

143.	Hosseini A, Serag A. Is synthetic data generation effective in maintaining clinical biomarkers? Investigating diffusion models across diverse imaging modalities. Front Artif Intell 2025 Jan 31;7:1454441. doi: 10.3389/frai.2024.1454441

144.	Jeyasri G, Karthiyayini R. Deep learning based retinal disease classification using an autoencoder and generative adversarial network. Biomedical Signal Processing and Control 2025 Oct;108:107852. doi: 10.1016/j.bspc.2025.107852

145.	Khazrak I, Zainaee S, M. Rezaee M, Ghasemi M, C. Green R. Feasibility of improving vocal fold pathology image classification with synthetic images generated by DDPM-based GenAI: a pilot study. Eur Arch Otorhinolaryngol 2025 Aug;282(8):4139–4153. doi: 10.1007/s00405-025-09443-4

146.	Kim M, Yoo J, Kwon S, Kim BJ, Pak CJ, Won CH, Moon S-H, Song WJ, Cha HG, Park KH. Diffusion-based skin disease data augmentation with fine-grained detail preservation and interpolation for data diversity. Wang Z, editor. PLoS One 2025 Oct 3;20(10):e0331404. doi: 10.1371/journal.pone.0331404

147.	King S, Hollenbenders Y, Reichenbach A. Efficient synthesis of 3D MR images for schizophrenia diagnosis classification with generative adversarial networks. Computer Methods and Programs in Biomedicine Update 2025 Jan 1;8:100197. doi: 10.1016/j.cmpbup.2025.100197

148.	Lai M, Marzi C, Citi L, Diciotti S. High-resolution conditional MR image synthesis through the PACGAN framework. Sci Rep 2025 Oct 1;15(1):34241. doi: 10.1038/s41598-025-16257-1

149.	Lajous H, Le Boeuf Fló A, Gordaliza PM, Esteban O, Marques F, Dunet V, Koob M, Bach Cuadra M. A dataset of synthetic, maturation-informed magnetic resonance images of the human fetal brain. Sci Data 2025 Apr 10;12(1):602. doi: 10.1038/s41597-025-04926-9

150.	Lyu Y, Tian X. MWG-UNet++: Hybrid Transformer U-Net Model for Brain Tumor Segmentation in MRI Scans. Bioengineering 2025 Jan 31;12(2):140. doi: 10.3390/bioengineering12020140

151.	Mizoguchi Y, Ito T, Yamada M, Tsutsumi T. Deep learning multi-classification of middle ear diseases using synthetic tympanic images. Acta Oto-Laryngologica 2025 Feb;145(2):134–139. doi: 10.1080/00016489.2024.2448829

152.	Parida C, Bilgaiyan S, Sagnika S, Roy S, Pandey A. 3D FusionNet for synthetic CT based lung cancer segmentation. Sci Rep 2025 Dec 3;15(1):43730. doi: 10.1038/s41598-025-29703-x

153.	Satapathy SM, Paul MG, Garg A, Bhatnagar S. GAN-Enhanced Hybrid Deep Learning with Explainable AI for Automated Cataract Diagnosis. J Med Syst 2025 Oct 2;49(1):123. doi: 10.1007/s10916-025-02249-1

154.	Shi S, Li H, Zhang Y, Wang X. Semantic information-guided attentional GAN-based ultrasound image synthesis method. Biomedical Signal Processing and Control 2025 Apr;102:107273. doi: 10.1016/j.bspc.2024.107273

155.	Shin SY, Wank SA, Summers RM. Size-Controllable Tumor Synthesis for Improved Detection of Small Bowel Carcinoid Tumors in CT Scans. IEEE Access 2025;13:151304–151315. doi: 10.1109/ACCESS.2025.3600672

156.	Sujini GN, Balakrishna S. Automated thyroid nodule classification in ultrasound imaging using a hybrid vision transformer and Wasserstein GAN with gradient penalty. Sci Rep 2025 Nov 19;15(1):40786. doi: 10.1038/s41598-025-24651-y

157.	Sukegawa S, Yoshii K, Hara T, Tanaka F, Taki Y, Inoue Y, Yamashita K, Nakai F, Nakai Y, Miyazaki R, Ishihama T, Miyake M. Optimizing dental implant identification using deep learning leveraging artificial data. Sci Rep 2025 Jan 29;15(1):3724. doi: 10.1038/s41598-025-87579-3

158.	Tan L, Lim G, Leong YY, Elangovan K, Ting D, Chan AS, Shen S. Functional blepharoptosis screening with generative augmented deep learning from external ocular photography. Orbit 2025 Nov 2;44(6):698–704. doi: 10.1080/01676830.2025.2497460

159.	Wang J, Wang K, Yu Y, Lu Y, Xiao W, Sun Z, Liu F, Zou Z, Gao Y, Yang L, Zhou H-Y, Miao H, Zhao W, Huang L, Zeng L, Guo R, Chong I, Deng B, Cheng L, Chen X, Luo J, Zhu M-H, Baptista-Hon D, Monteiro O, Li M, Ke Y, Li J, Zeng S, Guan T, Zeng J, Xue K, Oermann E, Luo H, Yin Y, Zhang K, Qu J. Self-improving generative foundation model for synthetic medical image generation and clinical applications. Nat Med 2025 Feb;31(2):609–617. doi: 10.1038/s41591-024-03359-y

160.	Wang D, Sklar B, Tian J, Gabriel R, Engelhard M, McNabb RP, Kuo AN. Improving Artificial Intelligence–based Microbial Keratitis Screening Tools Constrained by Limited Data Using Synthetic Generation of Slit-Lamp Photos. Ophthalmology Science 2025 May;5(3):100676. doi: 10.1016/j.xops.2024.100676

161.	Xu P, Lyu J, Lin L, Cheng P, Tang X. LF-SynthSeg: Label-Free Brain Tissue-Assisted Tumor Synthesis and Segmentation. IEEE J Biomed Health Inform 2025 Feb;29(2):1101–1112. doi: 10.1109/JBHI.2024.3489721

162.	Ye W, Zhang L, Jiang X, Yang D, Zhu Y. Dual-Stream Contrastive Learning for Medical Visual Representations Using Synthetic Images Generated by Latent Diffusion Model. IEEE Access 2025;13:129648–129658. doi: 10.1109/ACCESS.2025.3591544

163.	Yu W, Guo X, Li W, Liu X, Chen H, Yuan Y. ToothMaker: Realistic Panoramic Dental Radiograph Generation via Disentangled Control. IEEE Trans Med Imaging 2025 Dec;44(12):5233–5244. doi: 10.1109/TMI.2025.3588466

164.	Zafar J, Koc V, Zafar H. Dual-Stream Contrastive Latent Learning Generative Adversarial Network for Brain Image Synthesis and Tumor Classification. J Imaging 2025 Mar 28;11(4):101. doi: 10.3390/jimaging11040101

165.	Zeng P, Song R, Chen S, Li X, Li H, Chen Y, Gong Z, Cai G, Lin Y, Shi M, Huang K, Chen Z. Expert-guided StyleGAN2 image generation elevates AI diagnostic accuracy for maxillary sinus lesions. Commun Med 2025 May 20;5(1):185. doi: 10.1038/s43856-025-00907-6

166.	Zhang X, Ou N, Liu C, Zhuo Z, Matthews PM, Liu Y, Ye C, Bai W. Unsupervised brain MRI tumour segmentation via two-stage image synthesis. Medical Image Analysis 2025 May;102:103568. doi: 10.1016/j.media.2025.103568

167.	Zhou M, Wagner MW, Tabori U, Hawkins C, Ertl-Wagner BB, Khalvati F. Generating 3D brain tumor regions in MRI using vector-quantization Generative Adversarial Networks. Computers in Biology and Medicine 2025 Feb;185:109502. doi: 10.1016/j.compbiomed.2024.109502
