# Supplementary material for: Quality Assessment and Model Training Impact of Synthetic Images in Medical Diagnostic Imaging: Systematic Review
Source: JMIR AI. 2026 Oct 15;5:e81472. doi: 10.2196/81472 (PMC13671675; doi:10.2196/81472)
Supplement: Supplementary file 2 [file ai-v5-e81472-s003.docx]

Synthetic images in medical diagnostic imaging: a systematic review of quality assessment and model training impact

***Multimedia appendix 2 - PRISMA 2020, PRISMA-S and SWiM Checklists***

*Locations refer to the final manuscript dated 20 July 2026 and to the accompanying supplementary files. Page numbers correspond to the rendered Word files supplied for this revision.*

# PRISMA 2020 Checklist

| **Section and Topic** | **Item #** | **Checklist item** | **Location where item is reported** |
| --- | --- | --- | --- |
| **TITLE** | | | |
| **Title** | 1 | Identify the report as a systematic review. | Main manuscript, p. 1 |
| **ABSTRACT** | | | |
| **Abstract** | 2 | See the PRISMA 2020 for Abstracts checklist. | Main manuscript, p. 2 |
| **INTRODUCTION** | | | |
| **Rationale** | 3 | Describe the rationale for the review in the context of existing knowledge. | Main manuscript, p. 4 |
| **Objectives** | 4 | Provide an explicit statement of the objective(s) or question(s) the review addresses. | Main manuscript, p. 4, last paragraph |
| **METHODS** | | | |
| **Eligibility criteria** | 5 | Specify the inclusion and exclusion criteria for the review and how studies were grouped for the syntheses. | Main manuscript, pp. 5-9 (Information sources, search strategy and eligibility criteria; Data extraction and outcomes; Synthesis without meta-analysis); Multimedia Appendix 1, Tables S1-S7 |
| **Information sources** | 6 | Specify all databases, registers, websites, organisations, reference lists and other sources searched or consulted to identify studies. Specify the date when each source was last searched or consulted. | Main manuscript, p. 5; Multimedia Appendix 1, pp. 3-5 (PRISMA-S; search results by source) |
| **Search strategy** | 7 | Present the full search strategies for all databases, registers and websites, including any filters and limits used. | Multimedia Appendix 1, pp. 3-5 (full PubMed/MEDLINE, Scopus, IEEE Xplore, and Embase search strategies; limits, restrictions, and filters) |
| **Selection process** | 8 | Specify the methods used to decide whether a study met the inclusion criteria of the review, including how many reviewers screened each record and each report retrieved, whether they worked independently, and if applicable, details of automation tools used in the process. | Main manuscript, pp. 5-6 (two independent reviewers; discrepancies resolved by group discussion) |
| **Data collection process** | 9 | Specify the methods used to collect data from reports, including how many reviewers collected data from each report, whether they worked independently, any processes for obtaining or confirming data from study investigators, and if applicable, details of automation tools used in the process. | Main manuscript, Data Availability section; Multimedia Appendix 3 extraction spreadsheet; public GitHub repository containing study-level data and analysis scripts |
| **Data items** | 10a | List and define all outcomes for which data were sought. Specify whether all results that were compatible with each outcome domain in each study were sought and, if not, the methods used to decide which results to collect. | Main manuscript, pp. 6-8 (image quality, downstream performance, explainability); Multimedia Appendix 1, Tables S2-S6 |
| **Data items** | 10b | List and define all other variables for which data were sought. Describe any assumptions made about any missing or unclear information. | Main manuscript, pp. 6-8 (study characteristics, imaging modality, clinical domain, architecture, ratios, data source); Multimedia Appendix 1, Tables S1-S8 |
| **Study risk of bias assessment** | 11 | Specify the methods used to assess risk of bias in the included studies, including details of the tool(s) used, how many reviewers assessed each study and whether they worked independently, and if applicable, details of automation tools used in the process. | Main manuscript, pp. 8-9 (Risk of bias assessment); Multimedia Appendix 1, Table S7 and Figure S4 |
| **Effect measures** | 12 | Specify for each outcome the effect measure(s) used in the synthesis or presentation of results. | Main manuscript, p. 9 (descriptive synthesis; percentage change in downstream performance); pp. 14-18 (reported image-quality and downstream metrics) |
| **Synthesis methods** | 13a | Describe the processes used to decide which studies were eligible for each synthesis. | Main manuscript, pp. 5-9 (eligibility criteria, extracted outcomes, synthesis eligibility for image-quality and downstream analyses) |
| **Synthesis methods** | 13b | Describe any methods required to prepare the data for presentation or synthesis, such as handling of missing summary statistics, or data conversions. | Main manuscript, p. 9 (missing variance/sample-size information; percentage-change calculation); pp. 14-18; Multimedia Appendix 1, Tables S10-S13 |
| **Synthesis methods** | 13c | Describe any methods used to tabulate or visually display results of individual studies and syntheses. | Main manuscript, p. 9 (boxplots, UpSet plots, heatmaps, bar plots); Figures 1-7; Multimedia Appendix 1, supplementary figures and tables |
| **Synthesis methods** | 13d | Describe any methods used to synthesize results and provide a rationale for the choice(s). If meta-analysis was performed, describe the model(s), method(s) to identify statistical heterogeneity, and software package(s) used. | Main manuscript, p. 9 (structured narrative/descriptive synthesis; meta-analysis infeasible because variance/sample-size information was missing or incomplete in >80% of eligible studies; R version 4.4.0) |
| **Synthesis methods** | 13e | Describe any methods used to explore possible causes of heterogeneity among study results. | Main manuscript, p. 9 (stratification by imaging modality and dataset type); Results pp. 10-20; Discussion pp. 20-24 |
| **Synthesis methods** | 13f | Describe any sensitivity analyses conducted to assess robustness of the synthesized results. | Main manuscript, p. 8 (unweighted and linearly weighted kappa as sensitivity analyses for risk-of-bias agreement). No formal outcome sensitivity analysis was conducted because quantitative meta-analysis was infeasible. |
| **Reporting bias assessment** | 14 | Describe any methods used to assess risk of bias due to missing results in a synthesis. | No formal reporting-bias assessment was conducted because no meta-analysis was performed. Missing/incomplete statistical reporting is addressed in Methods p. 9, Risk of bias pp. 19-20, and Discussion pp. 22-24. |
| **Certainty assessment** | 15 | Describe any methods used to assess certainty (or confidence) in the body of evidence for an outcome. | No formal GRADE/certainty assessment was performed. Confidence in the evidence is interpreted through risk of bias, heterogeneity, and reproducibility limitations (Methods pp. 8-9; Results pp. 19-20; Discussion pp. 20-24). |
| **RESULTS** | | | |
| **Study selection** | 16a | Describe the results of the search and selection process, from the number of records identified in the search to the number of studies included in the review, ideally using a flow diagram. | Main manuscript, pp. 10-11 (Study selection and characteristics; Figure 1 PRISMA flow diagram); Multimedia Appendix 1, pp. 4-5 |
| **Study selection** | 16b | Cite studies that might appear to meet the inclusion criteria, but which were excluded, and explain why they were excluded. | Exclusion categories are described in the main manuscript, p. 5, and summarized in the PRISMA flow diagram, pp. 10-11. Individual excluded studies are not cited. |
| **Study characteristics** | 17 | Cite each included study and present its characteristics. | Main manuscript, Data Availability section; Multimedia Appendix 3 extraction spreadsheet; public GitHub repository containing study-level data and analysis scripts |
| **Risk of bias in studies** | 18 | Present assessments of risk of bias for each included study. | Main manuscript, pp. 19-20 (Risk of bias; Figure 7); Multimedia Appendix 1, Table S14 and Figure S4 |
| **Results of individual studies** | 19 | For all outcomes, present, for each study: (a) summary statistics for each group, where appropriate, and (b) an effect estimate and its precision, ideally using structured tables or plots. | Main manuscript, Data Availability section; Multimedia Appendix 3 extraction spreadsheet; public GitHub repository containing study-level data and analysis scripts |
| **Results of syntheses** | 20a | For each synthesis, briefly summarise the characteristics and risk of bias among contributing studies. | Main manuscript, pp. 10-20 (study characteristics; image quality; downstream performance; explainability; risk of bias); Multimedia Appendix 1, Tables S8-S14 |
| **Results of syntheses** | 20b | Present results of all statistical syntheses conducted. If meta-analysis was done, present the summary estimate and its precision and measures of statistical heterogeneity. If comparing groups, describe the direction of the effect. | Main manuscript, pp. 14-18 (Figures 4-6; descriptive and distributional syntheses; percentage-change analyses). No meta-analysis was performed. |
| **Results of syntheses** | 20c | Present results of all investigations of possible causes of heterogeneity among study results. | Main manuscript, pp. 10-20 (stratified descriptive results by modality, model family, metric type, and dataset type); Discussion pp. 20-24 |
| **Results of syntheses** | 20d | Present results of all sensitivity analyses conducted to assess the robustness of the synthesized results. | Main manuscript, pp. 19-20; Multimedia Appendix 1, Table S14 (risk-of-bias inter-rater reliability sensitivity analyses). Outcome sensitivity analyses were not conducted because no meta-analysis was performed. |
| **Reporting biases** | 21 | Present assessments of risk of bias due to missing results for each synthesis assessed. | No formal reporting-bias assessment was performed. Incomplete statistical reporting is summarized in the risk-of-bias results and discussion (main manuscript, pp. 19-20 and 22-24). |
| **Certainty of evidence** | 22 | Present assessments of certainty (or confidence) in the body of evidence for each outcome assessed. | No formal certainty assessment was performed; certainty is discussed narratively in relation to heterogeneity, risk of bias, and reproducibility (main manuscript, pp. 20-24). |
| **DISCUSSION** | | | |
| **Discussion** | 23a | Provide a general interpretation of the results in the context of other evidence. | Main manuscript, pp. 20-24 |
| **Discussion** | 23b | Discuss any limitations of the evidence included in the review. | Main manuscript, pp. 20-24 (heterogeneity, incomplete reporting, risk of bias, reproducibility) |
| **Discussion** | 23c | Discuss any limitations of the review processes used. | Main manuscript, pp. 23-24 |
| **Discussion** | 23d | Discuss implications of the results for practice, policy, and future research. | Main manuscript, pp. 23-24; Conclusion p. 24 |
| **OTHER INFORMATION** | | | |
| **Registration and protocol** | 24a | Provide registration information for the review, including register name and registration number, or state that the review was not registered. | Main manuscript, p. 2 (Study registration) and p. 6 (PROSPERO CRD42023369125) |
| **Registration and protocol** | 24b | Indicate where the review protocol can be accessed, or state that a protocol was not prepared. | Main manuscript, p. 6; References, p. 29 (PROSPERO record) |
| **Registration and protocol** | 24c | Describe and explain any amendments to information provided at registration or in the protocol. | Amendments included the addition of Embase and refinement of the detailed descriptive synthesis groups in response to the observed outcome heterogeneity. The eligibility criteria and broad review objectives remained unchanged. These amendments are described in the Methods and Multimedia Appendix 1. |
| **Support** | 25 | Describe sources of financial or non-financial support for the review, and the role of the funders or sponsors in the review. | Main manuscript, Funding Statement: “The authors declared no financial support was received for this work.” |
| **Competing interests** | 26 | Declare any competing interests of review authors. | Main manuscript, pp. 24-25 (Conflicts of Interest: none declared) |
| **Availability of data, code and other materials** | 27 | Report which data, code, and other materials are publicly available and where they can be found. | Main manuscript, Data Availability section; Multimedia Appendix 3 extraction spreadsheet; public GitHub repository containing study-level data and analysis scripts |

# PRISMA-S Checklist

PRISMA-S checklist for reporting literature searches in systematic reviews.

| **Section and Topic** | **Item #** | **Checklist item** | **Location where item is reported** |
| --- | --- | --- | --- |
| **Database name** | **1** | Name each individual database searched, stating the platform or provider used for each database. | Main manuscript, p. 5; Multimedia Appendix 1, pp. 3-4 (PubMed/MEDLINE, Scopus, IEEE Xplore, and Embase) |
| **Multi-database searching** | **2** | If databases were searched simultaneously on a single platform, state the name of the platform and list all databases searched. | Not applicable: databases were searched separately. Search strings and results by source are reported in Multimedia Appendix 1, pp. 3-4. |
| **Study registries** | **3** | List any study registries searched. | Multimedia Appendix 1, p. 3 (no study registries searched; rationale provided). |
| **Online resources and browsing** | **4** | Describe any online resources, websites, or browsing activities used to identify studies. | No online resources, websites, or browsing activities were used as additional sources for study identification. Only the four bibliographic databases were searched. |
| **Citation searching** | **5** | Indicate whether citation searching or reference-list checking was conducted, and describe the methods if used. | No backward or forward citation searching or reference-list checking was conducted. |
| **Contacts** | **6** | Indicate whether study authors, experts, manufacturers, or other contacts were approached to identify studies. | Study authors, experts, manufacturers, and other contacts were not approached to identify additional studies. |
| **Other methods** | **7** | Describe any additional methods used to identify studies. | No additional methods were used to identify studies. |
| **Full search strategies** | **8** | Include the search strategies for each database and information source, copied exactly as run. | Multimedia Appendix 1, pp. 3-5 (complete PubMed/MEDLINE, Scopus, IEEE Xplore, and Embase search strategies, copied as run). |
| **Limits and restrictions** | **9** | Specify any limits or restrictions applied to the search, and provide the rationale. | Main manuscript, p. 5; Multimedia Appendix 1, pp. 4-5 (full-text availability, English language, publication years, subject-area and content-type restrictions, including the Embase publication-type limits, with rationales). |
| **Search filters** | **10** | Indicate whether any published/validated search filters were used; if used, cite them. If filters were adapted or not validated, describe them. | Multimedia Appendix 1, pp. 4-5 (limits and filters; no validated methodological search filter applied unless specified). |
| **Prior work** | **11** | Indicate whether search strategies from prior reviews or other sources were used or adapted. | The search strategy was developed specifically for this review and was not copied or adapted from a previous systematic review. Search terms were organized around the main review concepts and adapted to each database. |
| **Updates** | **12** | Report any search updates, including dates and methods, or state that no update was conducted. | PubMed/MEDLINE and Scopus were searched on November 28, 2025; IEEE Xplore was searched on December 8, 2025; and Embase was added and searched on July 9, 2026. No further search update was conducted after these final database searches. |
| **Dates of searches** | **13** | Provide the date when each database or source was last searched. | Main manuscript, p. 5; Multimedia Appendix 1, pp. 3-4: PubMed/MEDLINE and Scopus, November 28, 2025; IEEE Xplore, December 8, 2025; Embase, July 9, 2026. |
| **Peer review** | **14** | Describe whether the search strategy was peer reviewed, including who reviewed it and which checklist or method was used. | The search strategy was not formally peer reviewed using PRESS or another structured peer-review method. |
| **Total records** | **15** | Document the total number of records identified from each source and overall. | Main manuscript, Study selection and characteristics; Multimedia Appendix 1, Search results by source: 7,302 records in total—3,218 from PubMed/MEDLINE, 1,805 from Scopus, 333 from IEEE Xplore, and 1,946 from Embase. |
| **Deduplication** | **16** | Describe deduplication methods and report the number of records removed. | Main manuscript, Study selection and characteristics; Multimedia Appendix 1, Record management and deduplication: records were deduplicated using DOI, followed by manual checking based on title, authors, journal, and publication year. A total of 3,621 duplicates were removed, leaving 3,681 records for screening. |

# Synthesis Without Meta-analysis (SWiM) Checklist

SWiM is used as an extension to PRISMA 2020. It is applied here to the quantitative synthesis of synthetic-image quality and downstream model performance where meta-analysis was not feasible. Page ranges refer to the current revised-manuscript layout; section and figure/table identifiers are provided to keep locations unambiguous if pagination changes.

| **Item** | **SWiM reporting requirement** | **Location in revised report** | **Completed-review details** |
| --- | --- | --- | --- |
| **1a** | Grouping studies for synthesis: describe and justify the population, intervention/exposure, outcome, design, or other groups used in each synthesis. | Methods—Data extraction and outcomes; Synthesis without meta-analysis (pp 7–11). Multimedia appendix 1, Tables S1–S8. | Evidence was grouped by review objective; human-reader vs automated quality assessment; task, metric family, metric, and modality; and downstream comparison, task, metric, and modality. |
| **1b** | Report and justify changes from the protocol to the groups used for synthesis. | Methods—Synthesis without meta-analysis (pp 8–11); Discussion—limitations (pp 24–28). | The protocol specified the two broad objectives but not every detailed analytic subgroup. Detailed groups were refined during extraction/analysis in response to outcome heterogeneity; eligibility criteria and objectives were unchanged. |
| **2** | Describe the standardized metric for each outcome, why it was chosen, and any transformations applied to reported effects. | Methods—Synthesis without meta-analysis (pp 9–11); Results—Image quality and downstream performance (pp 16–22). Multimedia appendix 1, Tables S3–S6 and S10–S13. | Rates/proportions were put on a 0–100% scale; AUC remained 0–1; scale scores retained native units; SSIM/MS-SSIM percentages were divided by 100; downstream change used 100 × (target−baseline)/\|baseline\| with sign reversal for lower-is-better metrics. |
| **3** | Describe and justify the method used to synthesize each outcome when meta-analysis was not possible. | Abstract—Methods (p 2); Methods—Synthesis without meta-analysis (pp 8–11); Results (pp 16–23). | Missing variance/sample-size information in >80% and clinical/methodological heterogeneity precluded inverse-variance meta-analysis. The review used structured tabulation, frequencies, unweighted distributions, and strict within-study paired changes; statistical-significance vote counting was not used. |
| **4** | State and justify criteria used to prioritize particular studies or results for the main synthesis or conclusions. | Methods—Impact on model performance (p 7); Synthesis without meta-analysis (pp 9–11); Discussion—limitations (pp 24–28). | Only strict matched within-study comparisons were used for downstream change. One primary best-performing ratio per study and analytical use was retained for compact ratio-frequency summaries, while all ratios remained in study-level data. Main-figure minimum-count rules were display criteria only. |
| **5** | State how heterogeneity in reported effects was examined when meta-analysis and its statistical extensions were unavailable. | Methods—Synthesis without meta-analysis (pp 9–11); Results—Figures 4–7 (pp 16–23); Discussion (pp 23–28). | Direction, magnitude, and dispersion were compared descriptively across modality, setting, generative-model family, assessment task, downstream task, dataset arm, metric, reporting completeness, and risk of bias. No subgroup meta-analysis or meta-regression was undertaken. |
| **6** | Describe how certainty in the synthesis findings was assessed. | Methods—Synthesis without meta-analysis (pp 10–11); Results—Risk of bias and reproducibility (pp 22–23); Discussion—limitations (pp 24–28). | No GRADE or numerical certainty rating was assigned because the evidence comprised heterogeneous AI development/validation studies rather than a common intervention-effect question. Interpretation considered study number/directness, consistency, statistical completeness, strict paired evidence, and risk of bias; comparative-performance certainty was judged limited. |
| **7** | Describe graphical and tabular presentation methods and the study characteristics used to order or structure the evidence. | Methods—Synthesis without meta-analysis (pp 9–11); Results—Figures 2–7 (pp 12–23). Multimedia appendix 1, Tables S8–S14; Multimedia appendix 3. | Study-characteristic and frequency tables, bar plots, UpSet plots, heatmaps, and boxplots with individual datapoints, medians, and IQRs were used. Evidence was structured by objective, modality, assessment/task, metric, comparison arm, and direction of benefit. |
| **8** | For each comparison and outcome, report the synthesized finding, its certainty, and the studies contributing to it, using language consistent with the review question. | Results—Image quality, downstream performance, risk of bias, and reproducibility (pp 16–23); Figures 4–7. Multimedia appendix 1, Tables S8–S14; Multimedia appendix 3 and public source data. | The revised Results distinguish descriptive distributions from pooled effects, report strict-pair medians as non-causal summaries, qualify comparative findings as limited-certainty evidence, and direct readers to study-level tables/source data identifying contributors. |
| **9** | Report limitations of the synthesis method or study groupings and explain their implications for the conclusions. | Discussion—risk of bias, reporting limitations, and synthesis limitations (pp 23–28). | Limitations include missing variance/sample-size data, unweighted medians, potential non-independence of multiple study-level datapoints, post-extraction grouping refinements, best-ratio selection, display thresholds, lack of formal certainty grades, and inability to estimate a pooled causal effect. |

# Checklist sources

Page MJ, McKenzie JE, Bossuyt PM, Boutron I, Hoffmann TC, Mulrow CD, et al. The PRISMA 2020 statement: an updated guideline for reporting systematic reviews. BMJ. 2021;372:n71. doi: 10.1136/bmj.n71.

Rethlefsen ML, Kirtley S, Waffenschmidt S, Ayala AP, Moher D, Page MJ, et al. PRISMA-S: an extension to the PRISMA Statement for Reporting Literature Searches in Systematic Reviews. Systematic Reviews. 2021;10:39. doi: 10.1186/s13643-020-01542-z.

Campbell M, et al. Synthesis without meta-analysis (SWiM) in systematic reviews: reporting guideline. BMJ. 2020;368:l6890. https://doi.org/10.1136/bmj.l6890
